# Supplementary material for: Extent of carbon nitride photocharging controls energetics of hydrogen transfer in photochemical cascade processes
Source: Nat Commun. 2023 Nov 24;14:7684. doi: 10.1038/s41467-023-43328-6 (PMC10674013; doi:10.1038/s41467-023-43328-6)
Supplement: Supplementary file 1 — Supplementary Information [file 41467_2023_43328_MOESM1_ESM.pdf]

Extent of Carbon Nitride Photocharging Controls Energetics of Hydrogen Transfer in Photochemical Cascade Processes

Supporting information

Oleksandr Savateev,*[,1,§] Karlo Nolkemper,[1,2] Thomas D. Kühne,[2] Vitaliy Shvalagin,[1] Yevheniia Markushyna,[1] Markus Antonietti[1]

[1] Department of Colloid Chemistry, Max Planck Institute of Colloids and Interfaces, Am Mühlenberg 1, 14476 Potsdam, Germany

[2] Dynamics of Condensed Matter and Center for Sustainable System Design, Chair of Theoretical Chemistry, University of Paderborn, Warburger Str. 100, D-33098 Paderborn, Germany

Corresponding author's email: oleksandr.savatieiev@mpikg.mpg.de

[§] Current affiliation: Department of Chemistry, The Chinese University of Hong Kong, Shatin, New Territories, Hong Kong, China. Email: oleksandrsavatieiev@cuhk.edu.hk

Contents

Supplementary Methods 6

1.1. Preparation of reagents and materials 6

Synthesis of methyl viologen bis(hexachlorophosphate), MV_2^+ $2PF_6^-$ 6

1.2. N_2 physisorption 7

1.3. NMR spectroscopy 7

1.4. High-resolution mass spectra (HR-MS) 7

1.5. Irradiance measurements 7

1.6. Light source 8

1.7. Measurement of the number of electrons stored in semiconductors 8

1.8. X-Ray diffraction 8

1.9. Cyclic voltammetry 9

1.10. Conversion of δ to *N* 9

1.11. Calculation of the number of heptazine units per iminium cation (N_{im}) 9

1.12. Kinetic studies 10

1.13. Screening of reaction conditions of benzylamine tetramerization 11

1.14. A general procedure of screening the reaction conditions of imine **1a** *aza*-pinacol coupling by mpg-CN-6nm-204, mpg-CN-8nm-193, mpg-CN-17nm-171 and CdS 11

1.15. A general procedure of screening the reaction conditions of imine **1a** *aza*-pinacol coupling by Na-PHI, H-PHI and $Ir(ppy)_2(dtbbpy)PF_6$ 11

1.16. An attempt to enable *aza*-pinacol coupling with photocharged mpg-CN-6nm-204(e^-/H^+) in dark 12

1.17. Synthesis of imines **1** 12

1.18. Synthesis of *R,S*-**2a-f** and *R,R*-**2a-f** using mpg-CN-8nm-193 13

1.19. Synthesis of *R,S*-**2a**-$^{15}N_2$ and *R,R*-**2a** -$^{15}N_2$ using K-PHI 17
1.20. Synthesis of *R,S*-**2a** and *R,R*-**2a** from **1a** by reduction with Zn dust 18
1.21. A general procedure trialkylamines conversion under anaerobic conditions........ 19
Supplementary Discussion 1........ 20
Supplementary Discussion 2........ 21
Supplementary Discussion 3........ 22
Supplementary Discussion 4........ 23
Supplementary Discussion 5........ 25
Supplementary Discussion 6........ 26
Supplementary Discussion 7........ 27
Supplementary Discussion 8........ 28
Supplementary Discussion 9........ 29
Supplementary Discussion 10........ 30
Supplementary Tables 31
Supplementary Table 1. Screening of conditions of benzylamine tetramerization by semiconductors and $Ir(ppy)_2(dtbbpy)PF_6$. 31
Supplementary Table 2. Control experiments of benzylamine tetramerization by mpg-CN-8nm-193. 33
Supplementary Table 3. Half-peak reduction and oxidation potentials........ 34
Supplementary Table 4. Summary of *δ* values for different semiconductors. 35
Supplementary Table 5. *Aza*-pinacol coupling of imine **1a** by mpg-CN-6nm-204. Screening of SEDs. 37
Supplementary Table 6. *Aza*-pinacol coupling of imine **1a** by mpg-CN-8nm-193. Screening of SEDs. 38
Supplementary Table 7. *Aza*-pinacol coupling of imine **1a** by mpg-CN-17nm-171. Screening of SEDs. 39
Supplementary Table 8. *Aza*-pinacol coupling of imine **1a** by CdS. Screening of SEDs. 40
Supplementary Table 9. *Aza*-pinacol coupling of imine **1a** by $Ir(ppy)_2(dtbbpy)PF_6$. Screening of SEDs. 41
Supplementary Table 10. *Aza*-pinacol coupling of imine **1a** by Na-PHI. Screening of SEDs. 42
Supplementary Table 11. *Aza*-pinacol coupling of imine **1a** by H-PHI. Screening of SEDs. 44
Supplementary Table 12. Control experiments in *aza*-pinacol coupling of imine **1a** by mpg-CN-8nm-193. 45
Supplementary Table 13. Study of Et_3N oxidation in *aza*-pinacol coupling. 46
Supplementary Table 14. Study of Et_3N and $^nBu_3N$ oxidation in *aza*-pinacol coupling. 47
Supplementary Table 15. Attempt to enable *aza*-pinacol coupling of imine **1a** by mpg-CN-6nm-204(e^-/H^+) in dark. 50
Supplementary Table 16. Summary of *N* values. 51
Supplementary Table 17. Summary of N_{im} values. 52
Supplementary Table 18. Stability of *R,S*-**2a** and *R,R*-**2a** under the conditions of photocatalysis. 53
Supplementary Table 19. Adsorption energies in solvated Na-PHI........ 54
Supplementary Table 20. Screening of reaction conditions of *aza*-pinacol coupling of imine **1a** using benzylamine as electron donor. 55
Supplementary Table 21. Summary of semiconductors textural properties. 56

Supplementary Table 22. Structure data for Na-PHI in .xyz format. 57

Supplementary Table 23. Structure data for H-PHI in .xyz format. 58

Supplementary Table 24. Variety of charges while hydrogen is adsorbed on Na-PHI, calculated via DDEC6. 59

Supplementary Table 25. Variety of charges while ammonium is adsorbed on Na-PHI, calculated via DDEC6. ..60

Supplementary Table 26. Charges of atoms in Na-PHI. 61

Supplementary Table 27. Charges of atoms in H-PHI. 62

Supplementary Table 28. Charges of atoms in Na-PHI solvated with 5-H_2O per Na atom. 63

Supplementary Table 29. Distance Na-Backbone in Na-PHI. 64

Supplementary Table 30. Results of kinetics study. 65

Supplementary Table 31. Correlation of benzylamine conversion with the surface area of the semiconductor.66

Supplementary Table 32. Fitting parameters. 67

Supplementary Table 33. Correlation of the number of electrons stored in g-CN materials with specific surface area. 68

Supplementary Table 34. Correlation of the number of electrons accumulated in Na-PHI with the number of Na^+ extracted from Na-PHI. 69

Supplementary Figures 70

Supplementary Fig. 1. **Overview of photocatalytic cascade processes mediated by molecular sensitizers.** 70

Supplementary Fig. 2. **Visualization of mpg-CN (melon-type g-CN)[18] and PHI unit cells.**[6] 71

Supplementary Fig. 3. **Structure of imidazolidine that is formed upon 2c condensation with acetaldehyde.** ...72

Supplementary Fig. 4. **Geometrically optimized structures of PHIs.** 73

Supplementary Fig. 5. **Proposed mechanism of benzylamine tetramerization mediated by graphitic carbon nitride semiconductor.** 74

Supplementary Fig. 6. **CV curves of MV^{2+} $2PF_6$, Et_3N, $^nBu_3N$ and $PhCH_2NH_2$ in 0.1 M solution of $^nBu_4N^+$ ClO_4^- in MeCN.** 75

Supplementary Fig. 7. **DRUV-vis spectra of Na-PHI photocharged using NH_4COOH.** 76

Supplementary Fig. 8. **Dependence of δ on concentration of benzylamine**. 77

Supplementary Fig. 9. **Benzylamine tetramerization mediated by Na-PHI**. 78

Supplementary Fig. 10. **Dependence of benzylamine conversion on the surface area of the semiconductor.** ..79

Supplementary Fig. 11. **Oxidation of Na-PHI photocharged using NH_4COOH**. 80

Supplementary Fig. 12. **Schematic representation of the setup and photographs of its elements that was used to perform kinetic measurements studies.** 81

Supplementary Fig. 13. **Results of kinetics study.** 82

Supplementary Fig. 14. **Dependence of δ on 1a conversion.** 83

Supplementary Fig. 15. **Characterization of Na-PHI recovered after benzylamine tetramerization.** 84

Supplementary Fig. 16. **Correlation of the number of Na^+ extracted from Na-PHI with the number of electrons stored in Na-PHI.** 85

Supplementary Fig. 17. **Charges of the poly(heptazine imide) backbone**. 86

Supplementary Fig. 18. **Structure of Na-PHI with water molecules in the micropore**. 87

Supplementary Fig. 19. **Imine 1a ^{1}H NMR spectrum in CD_3CN.** 88

Supplementary Fig. 20. **Imine 1a ^{13}C NMR spectrum in CD_3CN.**..........88
Supplementary Fig. 21. **Imine 1b ^{1}H NMR spectrum in $CDCl_3$.**..........89
Supplementary Fig. 22. **Imine 1b ^{1}H NMR spectrum in $CDCl_3$.**..........89
Supplementary Fig. 23. **Imine 1c ^{1}H NMR spectrum in CD_3CN.**..........90
Supplementary Fig. 24. **Imine 1c ^{13}C NMR spectrum in CD_3CN.**..........90
Supplementary Fig. 25. **Imine 1d ^{1}H NMR spectrum in CD_3CN.**..........91
Supplementary Fig. 26. **Imine 1d ^{13}C NMR spectrum in CD_3CN.**..........91
Supplementary Fig. 27. **Imine 1e ^{1}H NMR spectrum in CD_3CN.**..........92
Supplementary Fig. 28. **Imine 1e ^{13}C NMR spectrum in CD_3CN.**..........92
Supplementary Fig. 29. **Imine 1f ^{1}H NMR spectrum in CD_3CN.**..........93
Supplementary Fig. 30. **Imine 1f ^{13}C NMR spectrum in CD_3CN.**..........93
Supplementary Fig. 31. ***R*,*S*-2a ^{1}H NMR spectrum in $CDCl_3$.**..........94
Supplementary Fig. 32. ***R*,*S*-2a ^{13}C NMR spectrum in $CDCl_3$.**..........94
Supplementary Fig. 33. ***R*,*S*-2a-$^{15}N_2$ ^{1}H NMR spectrum in CD_3CN.**..........95
Supplementary Fig. 34. ***R*,*S*-2a-$^{15}N_2$ ^{13}C NMR spectrum in CD_3CN.**..........95
Supplementary Fig. 35. ***R*,*S*-2a-$^{15}N_2$ ^{15}N NMR spectrum in CD_3CN.**..........96
Supplementary Fig. 36. ***R*,*R*-2a ^{1}H NMR spectrum in $CDCl_3$.**..........96
Supplementary Fig. 37. ***R*,*R*-2a ^{13}C NMR spectrum in $CDCl_3$.**..........97
Supplementary Fig. 38. ***R*,*R*-2a-$^{15}N_2$ ^{1}H NMR spectrum in CD_3CN.**..........97
Supplementary Fig. 39. ***R*,*R*-2a-$^{15}N_2$ ^{13}C NMR spectrum in CD_3CN.**..........98
Supplementary Fig. 40. ***R*,*R*-2a-$^{15}N_2$ ^{15}N NMR spectrum in CD_3CN.**..........98
Supplementary Fig. 41. ***R*,*S*-2b ^{1}H NMR spectrum in $CDCl_3$.**..........99
Supplementary Fig. 42. ***R*,*S*-2b ^{13}C NMR spectrum in $CDCl_3$.**..........99
Supplementary Fig. 43. ***R*,*R*-2b ^{1}H NMR spectrum in $CDCl_3$.**..........100
Supplementary Fig. 44. ***R*,*R*-2b ^{13}C NMR spectrum in $CDCl_3$.**..........100
Supplementary Fig. 45. ***R*,*S*-2c ^{1}H NMR spectrum in $CDCl_3$.**..........101
Supplementary Fig. 46. ***R*,*S*-2c ^{13}C NMR spectrum in $CDCl_3$.**..........101
Supplementary Fig. 47. ***R*,*R*-2c ^{1}H NMR spectrum in $CDCl_3$.**..........102
Supplementary Fig. 48. ***R*,*R*-2c ^{13}C NMR spectrum in $CDCl_3$.**..........102
Supplementary Fig. 49. ***R*,*S*-2d ^{1}H NMR spectrum in $CDCl_3$.**..........103
Supplementary Fig. 50. ***R*,*S*-2d ^{13}C NMR spectrum in $CDCl_3$.**..........103
Supplementary Fig. 51. ***R*,*S*-2e ^{1}H NMR spectrum in $CDCl_3$.**..........104
Supplementary Fig. 52. ***R*,*S*-2e ^{13}C NMR spectrum in $CDCl_3$.**..........104
Supplementary Fig. 53. ***R*,*R*-2e ^{1}H NMR spectrum in $CDCl_3$.**..........105
Supplementary Fig. 54. ***R*,*R*-2e ^{1}H NMR spectrum in $CDCl_3$.**..........105
Supplementary Fig. 55. ***R*,*S*-2f ^{1}H NMR spectrum in $CDCl_3$.**..........106
Supplementary Fig. 56. ***R*,*S*-2f ^{13}C NMR spectrum in $CDCl_3$.**..........106

Supplementary Fig. 57. ***R*,*R*-2f ^{13}C NMR spectrum in $CDCl_3$.** 107
Supplementary Fig. 58. ***R*,*R*-2f ^{13}C NMR spectrum in $CDCl_3$.** 107
Supplementary Fig. 59. **MV^{2+} $2PF_6^-$ ^{1}H NMR spectrum in CD_3CN.** 108
Supplementary Fig. 60. **MV^{2+} $2PF_6^-$ ^{13}C NMR spectrum in CD_3CN.** 108
Supplementary Fig. 61. **^{1}H NMR spectrum of imidazolidine 3.** 109
Supplementary References 110

Supplementary Methods

1.1. Preparation of reagents and materials

Benzylamine (99%); ^{15}N-benzylamine (98 atom % ^{15}N); CdS (98+%); WO_3 (99.9%); $Ir(ppy)_3$ (99%); $Ir(ppy)_2(dtbbpy)PF_6$ (CAS 676525-77-2); TiO_2 (anatase particles <25 nm, ≥99%); melamine (99%) were purchased from Sigma-Aldrich. 4-(trifluoromethyl)benzylamine (98%) was purchased from Alfa-Aesar. 4-fluorobenzylamine (>98%), 4-tolualdehyde (>98%), 3-fluorobenzaldehyde (>97%) were purchased from TCI. 4-methoxybenzylamine (98%); 4-methylbenzylamine (98%) were purchased from Acros Organics. Acetonitrile (≥99.9%) was purchased from Honeywell. NaCl (99+%) was purchased from Thermo Scientific. These chemicals have been used as received without purification.

Synthesis of methyl viologen bis(hexachlorophosphate), $MV_2^{+}\ 2PF_6^{-}$

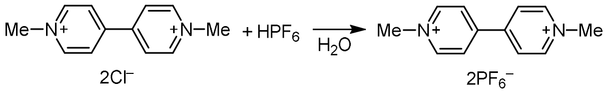

A solution of HPF_6 (15.3 g, 60 wt. % in water) was added dropwise to a stirred solution of methyl viologen dichloride hydrate (5.41 g) in water (40 mL). The white solid was filtered and washed with water. The solid was first dried at +60°C and residual pressure 10 mbar overnight and then at +60°C and residual pressure 0.01 mbar for 1 h. The solid was stored in the Ar-filled glove box. Yield: 8.2 g, white solid. ^{1}H NMR (400 MHz, CD_3CN) δ 8.85 (d, J = 7.0 Hz, 4H), 8.38 (d, J = 7.0 Hz, 4H), 4.40 (s, 6H). ^{13}C NMR (101 MHz, CD_3CN) δ 150.6, 147.4, 127.7, 49.5.

K-PHI has been prepared according to the reported procedure.[1] A mixture of 5-aminotetrazole (0.99 g) and eutectic mixture of LiCl/KCl (4.97 g, 9:11 wt. ratio) was placed in a steel ball mill cup. The steel ball was inserted and the cup was closed. The mixture of precursors was ground for 5 min at the operational frequency 25 Hz. The white powder was transferred into a porcelain crucible. Six crucibles with the identical composition of the precursors mixture was prepared. The crucibles were covered with porcelain lids, and placed in the chamber furnace. The temperature inside the furnace was increased from 20 to 600 °C within 4 h (ramp 2.4 K min^{-1}) under a flow of nitrogen (15 L min^{-1}), after which it was maintained at 600 °C for another 4 h. After that, the oven was allowed to cool slowly to room temperature overnight. The solids from the crucibles were transferred into a beaker, and deionized water (300 mL) and a stir bar were added. The suspension was stirred at room temperature for 4 h until it became homogeneous and no agglomerated particles were observed. The solid was separated by centrifugation (6 500 min^{-1}, 12 min). The aqueous solution was carefully removed and the residue transferred into a 2 mL safe-lock tube. The solid was washed with water (3 × 1.5 mL) by centrifugation (13 500 min^{-1}, 3 min) and dried under vacuum. Yield: 1.8 g.

Na-PHI (S_{SA} = 35 $m^2\ g^{-1}$) has been prepared according to the reported procedure.[2] Melamine (1 g) was thoroughly grinded with NaCl (10 g) in a mortar. The reaction mixture was transferred into a porcelain crucible which was covered with a lid. The crucible was placed in a chamber furnace and heated under a constant nitrogen flow (15 L min^{-1}) to 600 °C with a heating rate of 2.3 °C min^{-1}, held at 600 °C for 4 hours, then allowed to cool spontaneously to room temperature overnights. The crude product was removed from the crucible, washed with deionized water (1 L), isolated by filtration, then thoroughly washed with deionized water on the filter (1 L) and dried in a vacuum oven at 50 °C for 15 h.

Na-PHI (S_{SA} = 1 m^2 g^{-1}) has been prepared according to the procedure. A mixture of melamine (1.0 g) and NaCl (10 g) was ball milled (25 Hz, 5 min). Powder was transferred into a porcelain crucible, covered with a lid and placed in the muffle oven under N_2 flow (5 L min^{-1}). The temperature inside the oved was increased from 25°C to 600°C within 4 h and maintained at 600°C for 4 h. The oven was allowed to cool to room temperature spontaneously overnight. The content from the crucible was transferred into a beaker. Deionized water (40 mL) was added, and the slurry was stirred at 25°C for 1 h. The yellow solid was separated by centrifugation (4000 rpm, 10 min) followed by washing with water (3 x 10 mL) and drying in vacuum (60°C, 8 mbar) overnight. Yield: 0.42 g.

mpg-CN-6nm-204, mpg-CN-8nm-193 and mpg-CN-17nm-171 have been prepared according to the reported procedure.[3] LUDOX SM (30 wt. % solution in water, particle diameter 7 nm) was used for preparation of mpg-CN-6nm-204, LUDOX HS-40 (40 wt.% solution in water, particle diameter 12 nm) for mpg-CN-8nm-193, LUDOX AS-40 (40 wt. % solution in water, particle diameter 22 nm) – for mpg-CN-17nm-171. A mixture of cyanamide (3.0 g) and LUDOX (7.5 g) was stirred at room temperature for 30 min, followed by stirring at +60°C for 16h on the oil bath until water has completely evaporated. The white solid was transferred into a crucible and placed into the oven. The temperature was increased from +25°C to +550°C within 4 h and maintained at +550°C for 4 h followed be spontaneous cooling to room temperature. The solid was briefly ground in a mortar and transferred into a polypropylene bottle followed by addition of $(NH_4)_2HF_2$ (12 g, 210 mmol) and water (50 mL). The mixture was stirred at room temperature for 24 h. The solid was separated by centrifugation (4000 rpm, 10 min), washed repeatedly with water (3x45 mL) and ethanol (45 mL), and dried in vacuum (+50°C, 20 mbar) overnight.

1.2. N_2 physisorption

Measurements have been conducted on Quantachrome® Quadrasorb SI physisorption station and the data have been processed in QuadraWin™ v.5.11 software. Semiconductors have been degassed in vacuum (0.03 mbar) at +150°C for 15 h prior the measurements. N_2 sorption isotherms have been acquired at 77K. Pore size distribution has been determined by applying QSDFT equilibrium model to N_2 sorption isotherm. Surface area has been determined from the absorption branch of N_2 sorption isotherms in the range of P/P_0 values $0.05 \leq P/P_0 \leq 0.3$.

1.3. NMR spectroscopy

^1H and ^{13}C NMR spectra were recorded on Agilent 400 MHz (at 400 MHz for Protons, 100.6 MHz for Carbon-13 and 40.6 MHz for Nitrogen-15). Chemical shifts are reported in ppm versus solvent residual peak: $CDCl_3$ 7.26 ppm (^1H NMR), 77.16 ppm (^{13}C NMR); CD_3CN 1.94 ppm (^1H NMR), 118.26 ppm (^{13}C NMR) 244.6 ppm (^{15}N NMR).

1.4. High-resolution mass spectra (HR-MS)

HR-MS spectra were acquired using Waters XEVO G2-XS QTOF with Aquity H-Class (HPLC).

1.5. Irradiance measurements

Irradiance of the LED modules was measured using PM400 Optical Power and Energy Meter equipped with the integrating sphere S142C.

1.6. Light source

Unless explicitly specified, the following light sources with emission maximum and photon flux on the surface of photoreactors were used in screening experiments performed on 0.1-0.4 mmol scale of reagents: 465 nm, 10±2 mW cm^{-2}, 0.039±0.008 μmol s^{-1} cm^{-1}; 410 nm, 39±8 mW cm^{-2}, 0.13±0.03 μmol s^{-1} cm^{-1}; 365 nm, 16±4 mW cm^{-2}, 0.049±0.012 μmol s^{-1} cm^{-1}. In *aza*-pinacol coupling performed on 3.5 mmol scale of imines, LED with emission maximum at 465 nm and photon flux 4.3 μmol s^{-1} was used.

1.7. Measurement of the number of electrons stored in semiconductors

The measurements have been conducted according to the described procedure.[4] A glass vial was loaded with the semiconductor powder and transferred into the Ar-filled glove box (residual O_2 and H_2O concentration < 0.1 ppm). An electron donor (0.2 mmol), MeCN (V_1 = 2 mL) and a magnetic stirring bar were added and the vial was sealed with a PTFE-lined cap. The vial was removed from the glove box. The mixture was stirred for 24 h under illumination at 465 nm (10±2 mW cm^{-2}, in case of mpg-CN) or 410 nm (39±8 mW cm^{-2}, in case of Na-PHI). The temperature of the reaction mixture was maintained at 25±5°C by cooling with air fan or at 80°C by immersing the bottom of the vial into the oil bath. The vial was transferred into the glove box. A solution of MV^{2+} $2PF_6^-$, in MeCN (V_2 = 2 mL, 0.1 M, 0.2 mmol) was added to the reaction mixture. The reaction mixture was stirred for 5 min and allowed to stand for additional 5 min to let semiconductor particles precipitate. An aliquot (V_3) was transferred into the gas-tight quartz cuvette with the optical path L = 1 cm followed by addition of MeCN (V_4 = 2 mL). In the case of tertiary amines as electron donors, typically V_3 = 0.04 mL, while for benzylamine V_3 = 0.3 mL. The cuvette was sealed with PTFE-lined cap and removed from the glove box. Absorption of the solution at 605 nm and 900 nm (absorption due to light scattering by the incompletely precipitated particles) was measured. The amount of $MV^{\bullet+}$ PF_6^- formed upon MV^{2+} $2PF_6^-$ reduction, which is equal to the amount of electrons (n, μmol) stored in the semiconductor was calculated according to the equation (1):

$$n = \frac{(A_{605} - A_{900}) \cdot (V_1 + V_2) \cdot (V_3 + V_4) \cdot 10^6}{\varepsilon \cdot L \cdot V_3 \cdot 10^3} \quad (1)$$

where A_{605} – absorption at 605 nm. A_{900} – absorption at 900 nm. V_1 – volume of the reaction mixture in the photocharging experiment, mL. V_2 – volume of $MV^{\bullet+}$ PF_6^- solution, mL. V_3 – aliquot volume, mL. V_4 – MeCN volume, mL. ε – exctinction coefficient of methyl viologen radical at 605 nm, 13 900 M^{-1} cm^{-1}.[5] L – optical path, cm.

It was found that amines react slowly with MV^{2+} $2PF_6^-$. Thus, under the investigated concentrations of reagents, time of the reaction mixture quenching and the absorption measurements, Et_3N reduces spontaneously (n_0) up to ~$1.5 \cdot 10^{-6}$ mol of MV^{2+} $2PF_6^-$, while benzylamine – up to ~$0.5 \cdot 10^{-6}$ mol of MV^{2+} $2PF_6^-$. Therefore, specific concentration of electrons (mol g^{-1}) stored in the semiconductor was calculated taking into account this process using equation (2):

$$\delta = \frac{n - n_0}{m} \quad (2)$$

where n_0 – amount of $MV^{\bullet+}$ formed spontaneously by direct reduction of MV^{2+} $2PF_6^-$ with amine, mol. m – mass of a semiconductor, g.

Under the studied conditions, spontaneous interaction of amines with MV^{2+} $2PF_6^-$ sets the limit for semiconductor mass at ≥ 1 mg. Below this mass, the measurements are not reliable.

1.8. X-Ray diffraction

X-Ray diffraction patterns were recorded on Bruker D8 Advance diffractometer equipped with a scintillation counter detector with CuKα radiation (λ = 0.15418 nm) applying 2θ step size of 0.05° and counting time of 3 s per step.

1.9. Cyclic voltammetry

The measurements were performed according to the procedure reported earlier.[6] The electrochemical measurements were performed in the three electrode cell equipped with the Ar inlet and magnetic stir bar. Biologic potentiostat was used to control the potential of the working electrode (WE) and EC-Lab v. 11.46 software for data logging. Glassy carbon (diameter 3 mm) was used as a WE, Ag wire in $AgNO_3$ (0.01 M) with tetrabutylammonium perchlorate (0.1 M) in MeCN as a reference electrode (RE), Pt wire as the counter electrode. The measurements were performed at room temperature (20-25°C). A solution of tetrabutylammonium perchlorate (0.1 M) in MeCN was used as the electrolyte. The electrochemical cell was placed in the grounded Faraday's cage to reduce noise. The electrochemical cell was filled with the electrolyte (10 mL). The solution was stirred while bubbling dry argon for approx. 10 min at the rate approx. 5 mL min^{-1}. Cycling voltammetry (CV) curves were acquired every 2-3 min to confirm absence of the redox peak related to the $O_2/O_2^{\bullet-}$ redox couple centred at approx. −1.19 V vs. RE. The CV measurements were performed without stirring, while flow of argon was by-passed above the solution. When no apparent redox peaks at approx. −1.19 V vs. RE were observed, a substrate (20 μmol in the case of MV^{2+} $2PF_6^-$, Et_3N or $^nBu_3N$; or 100 μmol in case of $PhCH_2NH_2$) was added to the electrolyte. The mixture was briefly stirred to dissolve the compound. The CV curves were acquired scanning continuously the region from 0 V to positive potential, to negative and then back to 0 V. Namely: MV^{2+} $2PF_6^-$: 0 ⟶ +1 ⟶ –1.1 ⟶ 0 V, Et_3N: 0 ⟶ +1 ⟶ –1.05 ⟶ 0 V, $^nBu_3N$: 0 ⟶ +0.8 ⟶ –1.05 ⟶ 0 V, $PhCH_2NH_2$: 0 ⟶ +2 ⟶ –1.05 ⟶ 0 V. Typically 3 scans were acquired. The scanning rate was 50 mV s^{-1}. After the measurement of redox potentials were finished, ferrocene (3.72 mg, 20 μmol) was added to the electrolyte. The solution was stirred for approx. 2 tor to completely dissolve ferrocene. The CV measurements were repeated scanning the region from +0.5 V to −0.5 V at the scanning rate 50 mV s^{-1}. The potential of the WE (*E*, V vs. Fc^+/Fc redox potential) was then recalculated using the equation:

$$E = E_{WE} - E_{Fc^+/Fc} \tag{3}$$

where E_{WE} – potential of the WE vs. RE, V. $E_{Fc^+/Fc}$ – the redox potential of the Fc^+/Fc couple vs. the RE, determined as the average between reduction and oxidation, V.

1.10. Conversion of δ to *N*

N denotes the number of heptazine units per electron stored in g-CN materials. The specific concentration of electrons (δ, mol g^{-1}) stored in mpg-CN was converted into *N* according to the equation:

$$N = \frac{k \cdot S_{SA} \cdot 10^{18}}{S_{cf} \cdot \delta \cdot N_A} \tag{4}$$

where *k* – number of heptazine units per unit cell projected onto the specific cell facet. S_{SA} – specific surface area of the material, m^2 g^{-1}. S_{cf} – area of the single unit cell facet, nm^2. N_A – Avogadro's number, $6.02 \cdot 10^{23}$ mol^{-1}. See also Supplementary Fig. 2 for visualization of unit cells and cell facets.

1.11. Calculation of the number of heptazine units per iminium cation (N_{im})

The number of heptazine units (N_{im}) that can be covered by a single iminium cation that is generated upon tertiary amine oxidation is calculated according to the equation (5). It is assumed that the iminium cation is spherical in shape.

$$N_{im} = \frac{k \cdot S_{VDW}}{S_{cf}} = \frac{k \cdot \sqrt[3]{\frac{9\pi V_{VDW}^2}{16}}}{S_{cf}} \tag{5}$$

where S_{cf} – the area of the single unit cell facet, nm^2. *k* – the number of heptazine units per unit cell projected onto the specific cell facet. S_{VDW} – van der Waals cross-section of the iminium cation, nm^2. V_{VDW}

– van der Waals volume of the iminium cation, nm^3. See also Supplementary Fig. 2 for visualization of the unit cell and the cell facets.

1.12. Kinetic studies

The setup that was used to conduct kinetic studies is shown in Supplementary Fig. 12. To confirm that there is no leakage, the setup was evacuated to 0.2 mbar absolute pressure, disconnected from a vacuum pump and a vacuum meter. After 5 min the assembly was connected back to the vacuum pump and the vacuum meter. No prompt pressure increase in the system was detected suggesting absence of leakage.

A three-necked flask equipped with cooler, a magnetic stirrer and a temperature sensor was charged with MeCN (55 mL), Et_3N (0.98 mL, 7 mmol), imine **1a** (0.68 g, 3.5 mmol) and mpg-CN-8nm-193 (0.7 g). A rubber stopper with one inlet for Ar, one inlet for connection to T-shaped adaptor T_1 and Loop 1 was inserted into the remaining neck. The reaction mixture was purged with Ar for 5 min upon vigorous stirring.

A solution of methylviologen (4.2 mM) was prepared in a glove box (O_2 concentration < 0.1 ppm and H_2O concentration < 0.1 ppm) by dissolving MV_2^+ $2PF_6^-$ (0.21 mmol, 0.1 g) in MeCN (50 mL) in a pear-shaped flask. The flask was closed with the rubber stopper equipped with Loop 2 (V = 2.4 mL, 120 cm, PTFE, I.D. 1.6 mm, O.D. 3.2 mm), T-shaped connector (T_1) and Y-shaped connector (Y_1). Valves in the connectors were brought to the positions to isolate the content of the flask from the ambient environment. The flask with the attached connectors was removed from the glove box and connected to the setup.

The temperature of the reaction mixture was controlled at 25°C. Aliquots were taken at a certain time while irradiating the reaction mixture at 465 nm (photon flux 16 µmol s^{-1}). While performing sampling of the reaction mixture the following principles were used. 1) Transfer of the defined quantity of the liquid from one vessel through tubing into another vessel was enabled by applying differential pressure of approx. 0.1 bar: P = 1.1 bar (absolute) in the vessel from which liquid was transferred, P = 0 bar (atmospheric pressure) – in the receiver. 2) After the loop was filled with the exact volume of liquid, the end of the tubing was withdrawn from the bulk of liquid and connected to the gas head space. 3) The liquid from the loop was transferred into a vial or cuvette by applying differential pressure of approx. 0.1 bar. 3) The end of the loop tubing was inserted back into the bulk of solution and the next aliquot was taken.

Photocharged mpg-CN titration. Loop 2 was filled with MV_2^+ $2PF_6^-$ solution (2.4 mL) and Loop 1 was filled with the reaction mixture (0.1 mL). The content of the loops was transferred into the quartz cuvette, where MV^{2+} was allowed to oxidize photocharged mpg-CN forming persistent $MV^{\bullet+}$ PF_6^-. The valves in the connectors T_2 and Y_2 were brought to the position to isolate the content of the cuvette from the ambient environment. The cuvette was disconnected from the syringe, Ar/vacuum line and connector Y_1. The cuvette together with the connectors Y_2 and T_2 was transferred into the chamber of the UV-vis absorption spectrometer. Absorption at 605 nm (A_{605}) and 900 nm (A_{900}, to take into account absorption due to light scattering by incompletely precipitated mpg-CN) vs. MeCN was measured. We observed that the solution of MV^{2+} $2PF_6^-$ in MeCN in the pear-shaped flask gradually gains pale-blue tint. To take into account absorption at 605 nm, which is due to spontaneous reduction of methylviologen, periodically Loop 2 was filled with this MV^{2+} $2PF_6^-$ stock solution and transferred into the quartz cuvette without mixing with the reaction mixture followed by absorption measurement at 605 nm (A_{corr}). The number of electrons transferred from photocharged mpg-CN to MV^{2+} (n, µmol) and the specific concentration of electrons stored in mpg-CN (δ, mol g^{-1}) were calculated using equation (6) and (7):

$$n = \frac{(A_{605} - A_{900} - A_{corr}) \cdot (V_{Loop1} + V_{Loop2}) \cdot 10^3}{\varepsilon \cdot L} \quad (6)$$

where A_{605} – absorption at 605 nm. A_{900} – absorption at 900 nm. A_{corr} – absorption at 605 nm of MV^{2+} $2PF_6^-$ stock solution in MeCN. V_{Loop1} – Loop 1 volume, mL. V_{Loop2} – Loop 2 volume, mL. ε – extinction coefficient of methyl viologen radical at 605 nm, 13 900 M^{-1} cm^{-1}.[5] L – optical path, cm.
The specific concentration of electrons (µmol g^{-1}) stored in mpg-CN:

$$\delta = \frac{n \cdot V}{m \cdot V_{Loop1}} \quad (7)$$

where n – the amount of electrons transferred from the photocharged mpg-CN to MV^{2+}, µmol. V – initial volume of the reaction mixture, mL. m – mass of mpg-CN, g.
Conversion of imine 1a and Et_3N; yield of the coupling products and Et_2NH. A quartz cuvette was replaced by a glass vial. Approx. 0.5 mL of the reaction mixture was transferred into the glass vial. The content of the glass vial was exposed to air, transferred into an Eppendorf tube and centrifuged at 13000 rpm for 5 min. An aliquot (0.4 mL) was transferred into an NMR tube followed by addition of 1,3,5-trimthoxybenzene solution in MeCN-d_3 (0.3 mL, 50 mM) and ^{1}H NMR spectrum acquisition. Conversion and yield were calculated vs. the internal standard.

1.13. Screening of reaction conditions of benzylamine tetramerization

A mixture of benzylamine (0.2 mmol, 21 µL), a semiconductor (20 mg), MeCN (2 mL) and a magnetic stirring bar were placed into a 5 mL screw-capped glass tube. The mixture was degassed three times using freeze-pump-thaw procedure and refilled with Ar. The mixture was stirred under irradiation with an LED for 24 h. After irradiation was stopped, a solution of 1,3,5-trimethoxybenzene (0.1 mL, 0.2 M) in CH_3CN was added to the reaction mixture. The reaction mixture was transferred into a 2 mL Eppendorf tube and centrifuged (13300 rpm, 3 min). The supernatant layer was separated. The semiconductor was washed with CH_2Cl_2 (3 x 2 mL) followed by centrifugation. The organic phases were combined in a flask and solvent was evaporated in vacuum (50°C, 150 mbar). The residue was dissolved in $CDCl_3$ (0.9 mL) and analyzed by ^{1}H NMR.

1.14. A general procedure of screening the reaction conditions of imine **1a** *aza*-pinacol coupling by mpg-CN-6nm-204, mpg-CN-8nm-193, mpg-CN-17nm-171 and CdS

A mixture of imine **1a** (0.1 mmol, 19.5 µL), a semiconductor (20 mg), a sacrificial electron donor (SED), solvent and a magnetic stirring bar were placed into a 4 mL screw-capped vial. The mixture was degassed three times using freeze-pump-thaw procedure and refilled with Ar. The mixture was stirred under irradiation with blue LED (465 nm, 10±2 mW cm^{-2}) for 24 h at +25±5°C by cooling with air fan. Irradiation was ceased and a solution of 1,3,5-trimethoxybenzene (0.1 mL, 0.2 M) in CH_3CN was added to the reaction mixture. The reaction mixture was transferred into 2 mL Eppendorf tube and centrifuged (13300 rpm, 3 min). The supernatant layer was separated. The semiconductor was washed with CH_2Cl_2 (3 x 2 mL) followed by centrifugation. The organic phases were combined in a flask and solvent was evaporated in vacuum (50°C, 150 mbar). The residue was dissolved in $CDCl_3$ (0.9 mL) and analyzed by ^{1}H NMR.

1.15. A general procedure of screening the reaction conditions of imine **1a** *aza*-pinacol coupling by Na-PHI, H-PHI and $Ir(ppy)_2(dtbbpy)PF_6$

A mixture of imine **1a** (0.1 mmol, 19.5 µL), $Ir(ppy)_2(dtbbpy)PF_6$ (1.8 mg, 2 mol. %), or Na-PHI (20 mg), or H-PHI (20 mg), a sacrificial electron donor (SED), solvent and a magnetic stirring bar were placed into a 5 mL screw-capped tube. The mixture was degassed three times using freeze-pump-thaw procedure and refilled with Ar. The mixture was stirred under irradiation with purple LED (410 nm, 39±8 mW cm^{-2}) for 24 h at +25±5°C. Irradiation was ceased and solution of 1,3,5-trimethoxybenzene (0.1 mL, 0.2 M) in CH_3CN

was added to the reaction mixture. The reaction mixture was transferred into a 2 mL Eppendorf tube and centrifuged (13300 rpm, 3 min). The supernatant layer was separated. The semiconductor was washed with CH_2Cl_2 (3 x 2 mL) followed by centrifugation. The organic phases were combined in a flask. In the case of homogeneous Ir(ppy)$_2$(dtbbpy)PF_6, the catalyst was not separated. The solvent was evaporated in vacuum (50°C, 150 mbar). The residue was dissolved in $CDCl_3$ (0.9 mL) and analyzed by ^{1}H NMR.

1.16. An attempt to enable *aza*-pinacol coupling with photocharged mpg-CN-6nm-204(e$^-$/H$^+$) in dark

A mixture of mpg-CN-6nm-204 (20 mg), Et_3N (0.2 mmol, 28 µL) in CH_3CN (2 mL) was degassed by freeze-pump-thaw procedure three times and refilled with Ar. The mixture was stirred under illumination with blue light (465 nm, 10±2 mW cm^{-2}) for 24 h at 25±5°C. A vial was transferred into an Ar-filled glove box with O_2 concentration < 0.1 ppm and H_2O concentration < 0.1 ppm. To the suspension, imine **1a** (0.029 mmol, 5.6 µL) was added. The vial was sealed and removed from the glove box. The mixture was stirred in the dark at the given temperature for 24 h. After irradiation was stopped, a solution of 1,3,5-trimethoxybenzene (0.1 mL, 0.2 M) in CH_3CN was added to the reaction mixture. The reaction mixture was transferred into a 2 mL Eppendorf tube and centrifuged (13300 rpm, 3 min). The supernatant layer was separated. The semiconductor was washed with CH_2Cl_2 (3 x 2 mL) followed by centrifugation. The organic phases were combined in a flask and solvent was evaporated in vacuum (50°C, 150 mbar). The residue was dissolved in $CDCl_3$ (0.9 mL) and analyzed by ^{1}H NMR.

1.17. Synthesis of imines 1

Imines were synthesized by mixing benzylic amine (typically 10-100 mmol) and aryl ring-substituted benzaldehyde in 1:1 molar ratio in EtOH (typically 10-50 mL) followed by stirring at reflux for 1 h. If upon cooling to room temperature imine precipitated, it was filtered and washed with EtOH. If upon cooling to room temperature imine did not precipitate, EtOH was concentrated in vacuum (+50°C, 10 mbar). If imine liquid at room temperature it was used without further purification; if imine is solid – it was recrystallized from pentane, hexane or methyl *tert*-butyl ether (MTBE).

(*E*)-N-benzyl-1-phenylmethanimine **1a**

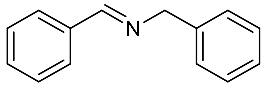

Yield: 99%, pale-yellow oil. ^{1}H NMR (400 MHz, CD_3CN) δ 8.46 (t, *J* = 1.5 Hz, 1H), 7.80 – 7.75 (m, 2H), 7.48 – 7.41 (m, 3H), 7.35 (d, *J* = 4.4 Hz, 4H), 7.30 – 7.25 (m, 1H), 4.77 (d, *J* = 1.6 Hz, 2H). ^{13}C NMR (101 MHz, CD_3CN) δ 162.7, 140.8, 137.4, 131.7, 129.6, 129.4, 129.0, 128.9, 127.8, 65.5.

(*E*)-N-(4-fluorobenzyl)-1-(3-fluorophenyl)methanimine **1b**

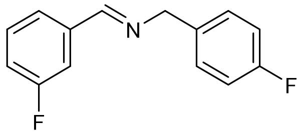

Yield: 91%, pale-yellow oil. ^{1}H NMR (400 MHz, $CDCl_3$) δ 8.39 (q, *J* = 1.5 Hz, 1H), 7.60 – 7.50 (m, 2H), 7.41 (td, *J* = 7.9, 5.6 Hz, 1H), 7.36 – 7.30 (m, 2H), 7.16 (tdd, *J* = 8.4, 2.7, 1.2 Hz, 1H), 7.10 – 7.03 (m, 2H), 4.82 (s, 2H). ^{13}C NMR (101 MHz, $CDCl_3$) δ 163.1 (d, *J* = 252.5 Hz), 162.2 (d, *J* = 242.4 Hz), 160.8 (d, *J* = 2.9 Hz), 138.4 (d, *J* = 7.3 Hz), 134.8 (d, *J* = 3.3 Hz), 130.3 (d, *J* = 8.1 Hz), 129.6 (d, *J* = 8.1 Hz), 124.5 (d, *J* = 2.9 Hz), 118.0 (d, *J* = 21.6 Hz), 115.5 (d, *J* = 21.3 Hz), 114.5 (d, *J* = 22.4 Hz), 64.2.

(*E*)-N-(4-methylbenzyl)-1-(p-tolyl)methanimine **1c**

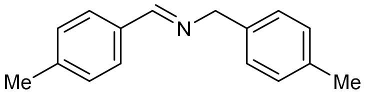

Yield: 85%, white solid (crystallized from EtOH). ^{1}H NMR (400 MHz, CD_3CN) δ 8.38 (s, 1H), 7.65 (d, *J* = 8.2 Hz, 2H), 7.25 (d, *J* = 7.9 Hz, 2H), 7.21 (d, *J* = 8.1 Hz, 2H), 7.15 (d, *J* = 7.8 Hz, 2H), 4.69 (s, 2H), 2.36 (s, 3H), 2.31 (s, 3H). ^{13}C NMR (101 MHz, CD_3CN) δ 162.3, 142.0, 137.9, 137.4, 134.9, 130.3, 130.0, 128.9, 65.3, 21.5, 21.1.

(*E*)-N-(4-methoxybenzyl)-1-(p-tolyl)methanimine **1d**

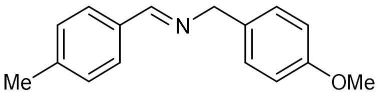

Yield: 98%, white solid (recrystallized from MTBE). ^{1}H NMR (400 MHz, CD_3CN) δ 8.37 (s, 1H), 7.64 (d, *J* = 8.2 Hz, 2H), 7.24 (d, *J* = 8.8 Hz, 4H), 6.89 (d, *J* = 8.8 Hz, 2H), 4.67 (s, 2H), 3.76 (s, 3H), 2.36 (s, 3H). ^{13}C NMR (101 MHz, CD_3CN) δ 162.1, 159.6, 142.0, 134.9, 132.9, 130.3, 130.2, 128.9, 114.7, 65.0, 55.8, 21.5.

(*E*)-1-p-tolyl-N-(4-(trifluoromethyl)benzyl)methanimine **1e**

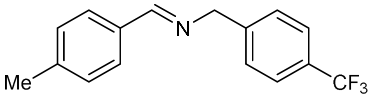

Yield: 86%, white solid (recrystallized from pentane). ^{1}H NMR (400 MHz, CD_3CN) δ 8.43 (s, 1H), 7.69 – 7.64 (m, 4H), 7.53 (d, *J* = 7.9 Hz, 2H), 7.26 (d, *J* = 7.9 Hz, 2H), 4.81 (s, 2H), 2.37 (s, 3H). ^{13}C NMR (101 MHz, CD_3CN) δ 163.5, 145.8 (d, *J* = 1.8 Hz), 142.3, 134.7, 130.3, 129.3, 129.0, 126.2 (q, *J* = 3.4 Hz), 125.6 (q, *J* = 245.8 Hz), 64.6, 21.5.

(*E*)-N-(4-fluorobenzyl)-1-(p-tolyl)methanimine **1f**

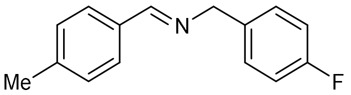

Yield: 87%, white solid (recrystallized from pentane). ^{1}H NMR (400 MHz, CD_3CN) δ 8.39 (s, 1H), 7.65 (d, *J* = 8.2 Hz, 2H), 7.38 – 7.31 (m, 2H), 7.25 (d, *J* = 7.9 Hz, 2H), 7.12 – 7.03 (m, 2H), 4.71 (s, 2H), 2.36 (s, 3H). ^{13}C NMR (101 MHz, CD_3CN) δ 162.7 (d, *J* = 242.1 Hz), 162.7, 142.1, 137.1 (d, *J* = 2.9 Hz), 134.8, 130.7 (d, *J* = 8.1 Hz), 130.3, 129.0, 115.9 (d, *J* = 21.6 Hz), 64.6, 21.5.

1.18. Synthesis of *R,S*-**2a-f** and *R,R*-**2a-f** using mpg-CN-8nm-193

A mixture of imine **1** (3.5 mmol), mpg-CN-8nm-193 (0.7 g), MeCN (70 mL) and Et_3N (0.98 mL, 7 mmol) and a magnetic stirring bar were loaded into a three-neck glass tube equipped with a cold-finger, an inlet for N_2 and a temperature sensor that was connected to a thermostat. The mixture was purged with N_2 for 5 min and held under a positive pressure of N_2 of ~0.1 bar to avoid possible air leakage into the reactor. The mixture was stirred under irradiation by blue LED (465 nm, 4.3 µmol s^{-1}) for 24 h. The temperature was set to 25°C and controlled by a thermostat. The reaction progress was monitored by ^{1}H NMR. Upon complete conversion of the imine **1**, the reaction mixture was concentrated in vacuum (+50°C, 100 mbar). The resultant solid was washed with CH_2Cl_2 (4 x 8 mL) and separated by centrifugation (13000 rpm, 3 min). Organic washings were combined and filtered through a 0.3 µm PTFE syringe filter. The solution was diluted with CH_2Cl_2 to a total volume of 30 mL. An aliquot (0.5 mL) was mixed with a solution of internal standard, 1,3,5-trimethoxybenzene (0.1 mL, 0.2 M in MeCN), and concentrated in vacuum (+50°C, 100 mbar). The yield of *R,S*-**2** and *R,R*-**2** isomers was calculated from the ^{1}H NMR spectra. The whole

amount of the *R,S*-**2** and *R,R*-**2** isomers solution in CH_2Cl_2 was concentrated in vacuum (+50°C, 50 mbar). The residue was triturated with pentane, the solid was filtered and washed with a small amount of pentane. Recrystallization from MTBE afforded *R,S*-**2**. The pentane solution was concentrated in vacuum (+50°C, 500 mbar). The oily residue was purified by column chromatography using a stationary phase of basic Al_2O_3 (65 g). The *R,R*-**2** isomer was obtained by washing the column with equal volumes of eluent with gradually increasing polarity – 1) hexane 2x100 mL, 2) hexane:ethylacetate (19:1) 2x100 mL, 3) hexane:ethylacetate (9:1) 2x100 mL, 4) hexane:ethylacetate (4:1) 3x100 mL.

(1*R*,2*S*)-N^1,N^2-dibenzyl-1,2-diphenylethane-1,2-diamine

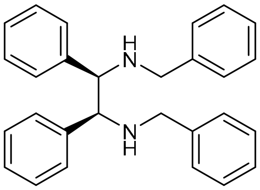

R,S-**2a**

Yield: 33%, white solid. ^{1}H NMR (400 MHz, $CDCl_3$) δ 7.35 – 7.25 (m, 10H), 7.23 – 7.15 (m, 6H), 6.98 – 6.92 (m, 4H), 3.75 (s, 2H), 3.53 (d, *J* = 13.7 Hz, 2H), 3.29 (d, *J* = 13.8 Hz, 2H), 1.82 (s, 2H). ^{13}C NMR (101 MHz, $CDCl_3$) δ 140.7, 140.3, 128.7, 128.5, 128.3, 128.0, 127.8, 126.8, 67.2, 51.0. HRMS (m/z): [M+H]$^+$ calcd. for $C_{28}H_{29}N_2^+$, 393.2325; found, 393.2304.

(1R,2R)-N^1,N^2-dibenzyl-1,2-diphenylethane-1,2-diamine

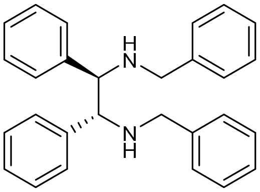

R,R-**2a**

Yield: 28%, colorless oil. ^{1}H NMR (400 MHz, $CDCl_3$) δ 7.33 – 7.22 (m, 10H), 7.19 – 7.14 (m, 6H), 7.07 – 7.05 (m, 4H), 3.76 (s, 2H), 3.71 (d, *J* = 13.1 Hz, 2H), 3.53 (d, *J* = 13.3 Hz, 2H). ^{13}C NMR (101 MHz, $CDCl_3$) δ 140.3, 140.1, 128.5, 128.3, 128.2, 128.1, 127.3, 127.1, 68.2, 51.3. HRMS (m/z): [M+H]$^+$ calcd. for $C_{28}H_{29}N_2^+$, 393.2325; found, 393.2309.

(1R,2S)-N[1],N[2]-bis(4-fluorobenzyl)-1,2-bis(3-fluorophenyl)ethane-1,2-diamine

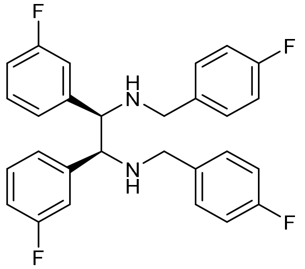

R,S-**2b**

Yield: 34%. The isomer was obtained by recrystallization of a mixture of *R,S*-**2b** and *R,R*-**2b** (1:0.72) from MTBE. ^{1}H NMR (400 MHz, $CDCl_3$) δ 7.31 – 7.25 (m, 2H), 7.02 – 6.89 (m, 14H), 3.68 (s, 2H), 3.51 (d, *J* = 13.7 Hz, 2H), 3.28 (d, *J* = 13.6 Hz, 2H), 1.66 (s, 2H). ^{13}C NMR (101 MHz, $CDCl_3$) δ 163.2 (d, *J* = 242.4 Hz), 162.0 (d, *J* = 252.5 Hz), 143.4 (d, *J* = 6.6 Hz), 135.7 (d, *J* = 3.3 Hz), 130.0 (d, *J* = 8.1 Hz), 129.5 (d, *J* = 8.1 Hz), 124.4 (d, *J* = 2.6 Hz), 115.3 (d, *J* = 20.2 Hz), 115.1 (d, *J* = 20.2 Hz), 115.0 (d, *J* = 20.2 Hz), 66.6, 50.3. HRMS (m/z): $[M+H]^+$ calcd. for $C_{28}H_{25}F_4N_2^+$, 465.1948; found, 465.1924.

(1R,2R)-N[1],N[2]-bis(4-fluorobenzyl)-1,2-bis(3-fluorophenyl)ethane-1,2-diamine

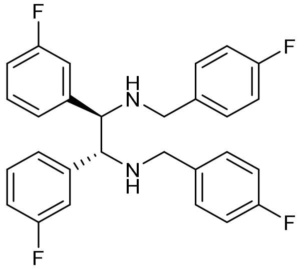

R,R-**2b**

Yield: 16% (a mixture of *R,R*-**2b**:*R,S*-**2b** = 1.0:0.15). ^{1}H NMR (400 MHz, $CDCl_3$) δ 7.21 – 7.16 (m, 4H), 7.15 – 7.10 (m, 2H), 7.00 – 6.96 (m, 6H), 6.88 – 6.76 (m, 6H), 3.66 (s, 2H), 3.64 (d, *J* = 13.3 Hz, 2H), 3.46 (d, *J* = 13.3 Hz, 2H), 2.55 (br.s, 2H). ^{13}C NMR (101 MHz, $CDCl_3$) δ 163.0 (d, *J* = 246.4 Hz), 162.1 (d, *J* = 246.4 Hz), 143.3, 135.5, 129.8 (d, *J* = 10.1 Hz), 129.8 (d, *J* = 10.1 Hz), 123.8 (d, *J* = 2.9 Hz), 115.3 (d, *J* = 21.3 Hz), 114.6 (d, J = 21.2 Hz), 114.4 (d, J = 21.2 Hz), 67.6, 50.6. HRMS (m/z): $[M+Na]^+$ calcd. for $C_{28}H_{24}F_4N_2Na^+$, 487.1768; found, 487.1572.

(1R,2S)-N[1],N[2]-bis(4-methylbenzyl)-1,2-di-p-tolylethane-1,2-diamine

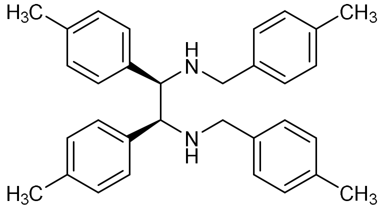

R,S-**2c**

Yield: 32%, white solid. ^{1}H NMR (400 MHz, $CDCl_3$) δ 7.17 (d, *J* = 8.2 Hz, 4H), 7.13 (d, *J* = 8.2 Hz, 4H), 7.02 (d, *J* = 7.9 Hz, 4H), 6.87 (d, *J* = 7.9 Hz, 4H), 3.72 (s, 2H), 3.49 (d, *J* = 13.6 Hz, 2H), 3.26 (d, *J* = 13.6 Hz, 2H), 2.37 (s, 6H), 2.30 (s, 6H), 1.79 (s, 3H). ^{13}C NMR (101 MHz, $CDCl_3$) δ 137.9, 137.4, 137.3, 136.2, 129.2, 128.9, 128.7, 128.0, 67.0, 50.7, 21.3, 21.2. HRMS (m/z): $[M+H]^+$ calcd. for $C_{32}H_{37}N_2^+$, 449.2951; found, 449.2924.

(1R,2R)-N[1],N[2]-bis(4-methylbenzyl)-1,2-di-p-tolylethane-1,2-diamine

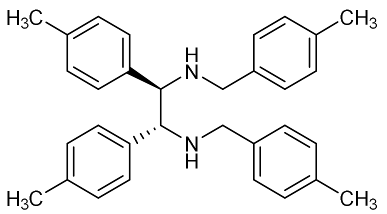

R,R-**2c**

Yield: 21%, pale-yellow oil. ^{1}H NMR (400 MHz, $CDCl_3$) δ 7.16 (d, *J* = 8.3 Hz, 4H), 7.13 (d, *J* = 8.4 Hz, 4H), 7.02 (d, *J* = 8.4 Hz, 4H), 6.98 (d, *J* = 8.3 Hz, 4H), 3.71 (s, 2H), 3.65 (d, *J* = 13.2 Hz, 2H), 3.47 (d, *J* = 13.2 Hz, 2H), 2.37 (s, 6H), 2.31 (s, 6H). ^{13}C NMR (101 MHz, $CDCl_3$) δ 138.3, 137.8, 136.3, 129.1, 128.8, 128.2, 128.0, 67.9, 51.1, 21.2. HRMS (m/z): $[M+H]^+$ calcd. for $C_{32}H_{37}N_2^+$, 449.2951; found, 449.2918.

(1R,2S)-N^1,N^2-bis(4-methoxybenzyl)-1,2-di-p-tolylethane-1,2-diamine

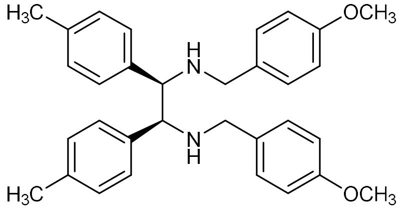

R,S-**2d**

Yield: 28%, white solid. ^{1}H NMR (400 MHz, $CDCl_3$) δ 7.18 (d, *J* = 8.2 Hz, 4H), 7.14 (d, *J* = 8.2 Hz, 4H), 6.88 (d, *J* = 8.7 Hz, 4H), 6.75 (d, *J* = 8.8 Hz, 4H), 3.78 (s, 6H), 3.68 (s, 2H), 3.46 (d, *J* = 13.6 Hz, 2H), 3.22 (d, *J* = 13.6 Hz, 2H), 2.37 (s, 6H), 1.67 (s, 3H). ^{13}C NMR (101 MHz, $CDCl_3$) δ 158.4, 137.9, 137.2, 132.6, 129.2, 129.2, 128.6, 113.6, 66.9, 55.4, 50.3, 21.3. HRMS (m/z): $[M+H]^+$ calcd. for $C_{32}H_{37}N_2O_2^+$, 481.2850; found, 481.2838.

(1R,2S)-1,2-di-p-tolyl-N^1,N^2-bis(4-(trifluoromethyl)benzyl)ethane-1,2-diamine

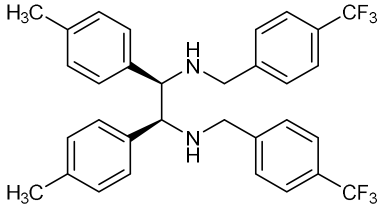

R,S-**2e**

Yield: 29%, white solid. ^{1}H NMR (400 MHz, $CDCl_3$) δ 7.46 (d, *J* = 8.3 Hz, 4H), 7.20 (d, *J* = 8.4 Hz, 4H), 7.17 (d, *J* = 8.4 Hz, 4H), 7.08 (d, *J* = 8.2 Hz, 4H), 3.65 (s, 2H), 3.57 (d, *J* = 14.3 Hz, 2H), 3.35 (d, *J* = 14.3 Hz, 2H), 2.39 (s, 6H), 1.71 (s, 2H). ^{13}C NMR (101 MHz, $CDCl_3$) δ 144.6, 137.7, 137.4, 129.4, 129.0 (q, *J* = 33.7 Hz), 128.5, 128.2, 125.2 (q, *J* = 3.9 Hz), 124.1 (q, *J* = 255.9 Hz), 67.1, 50.4, 21.3. HRMS (m/z): $[M]^{\bullet+}$ calcd. for $C_{32}H_{30}F_6N_2^{\bullet+}$, 557.2386; found, 557.2391.

(1R,2R)-1,2-di-p-tolyl-N^1,N^2-bis(4-(trifluoromethyl)benzyl)ethane-1,2-diamine

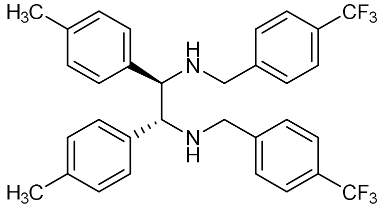

R,R-**2e**

Yield: 22%, pale-yellow oil. ^{1}H NMR (400 MHz, $CDCl_3$) δ 7.54 (d, *J* = 8.2 Hz, 4H), 7.32 (d, *J* = 8.1 Hz, 4H), 7.02 (d, *J* = 8.1 Hz, 4H), 6.98 (d, *J* = 8.3 Hz, 4H), 3.72 (d, *J* = 13.9 Hz, 2H), 3.69 (s, 2H), 3.54 (d, *J* = 13.9 Hz, 2H), 2.30 (s, 6H). ^{13}C NMR (101 MHz, $CDCl_3$) δ 144.8 (q, *J* = 1.5 Hz), 137.7, 136.8, 129.1 (q, *J* = 33.7 Hz), 129.0, 128.4, 127.9, 125.3 (q, *J* = 3.4 Hz), 124.4 (q, *J* = 272.7 Hz), 67.8, 50.9, 21.2. HRMS (m/z): $[M]^{\bullet+}$ calcd. For $C_{32}H_{30}F_6N_2^{\bullet+}$, 557.2386; found, 557.2403.

(1R,2S)-N^1,N^2-bis(4-fluorobenzyl)-1,2-di-p-tolylethane-1,2-diamine

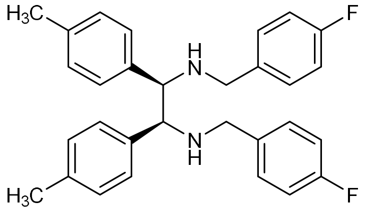

R,S-**2f**

Yield: 25%, white solid. ^{1}H NMR (400 MHz, $CDCl_3$) δ 7.16 (d, *J* = 2.6 Hz, 8H), 6.94 – 6.86 (m, 8H), 3.65 (s, 2H), 3.48 (d, *J* = 13.8 Hz, 2H), 3.25 (d, *J* = 13.7 Hz, 2H), 2.38 (s, 6H), 1.71 (s, 2H). ^{13}C NMR (101 MHz, $CDCl_3$) δ 161.8 (d, *J* = 244.3 Hz), 137.6, 137.5, 136.1 (d, *J* = 2.9 Hz), 129.5 (d, *J* = 7.7 Hz), 129.3, 128.5, 115.0 (d, *J* = 21.3 Hz), 66.9, 50.2, 21.3. HRMS (m/z): $[M+H]^+$ calcd. for $C_{30}H_{31}F_2N_2^+$, 457.2450; found, 457.2439.

(1R,2R)-N^1,N^2-bis(4-fluorobenzyl)-1,2-di-p-tolylethane-1,2-diamine

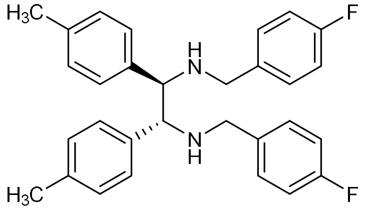

R,R-**2f**

Yield: 14% (contains ~18% of *R,S*-**2f**), pale-yellow oil. ^{1}H NMR (400 MHz, $CDCl_3$) δ 7.17 – 7.13 (m, 4H), 7.00 – 6.93 (m, 12H), 3.64 (s, 2H), 3.60 (d, *J* = 13.2 Hz, 2H), 3.42 (d, *J* = 13.3 Hz, 2H), 2.38 (s, 1H), 2.28 (s, 6H), 2.02 (s, 2H). ^{13}C NMR (101 MHz, $CDCl_3$) δ 161.9 (d, *J* = 244.3 Hz), 138.0, 136.6, 136.4 (d, *J* = 3.3 Hz), 129.7 (d, *J* = 8.1 Hz), 128.9, 127.9, 115.1 (d, *J* = 20.9 Hz), 67.8, 50.6, 21.2. HRMS (m/z): $[M+H]^+$ calcd. for $C_{30}H_{31}F_2N_2^+$, 457.2450; found 457.2429.

1.19. Synthesis of *R,S*-**2a**-$^{15}N_2$ and *R,R*-**2a** -$^{15}N_2$ using K-PHI

A mixture of benzylamine-^{15}N (0.2 mmol, 22 µL), K-PHI (20 mg) and MeCN (2 mL) and a magnetic stirring bar were placed into a 5 mL screw-capped glass tube. The mixture was degassed three times using freeze-pump-thaw procedure and refilled with Ar. The mixture was stirred under irradiation with blue LED

(461 nm) for 48 h. The reaction mixture was transferred into a 2 mL Eppendorf tube and centrifuged (13000 rpm, 5 min). The supernatant layer was separated. K-PHI was washed with $CHCl_3$ (3 x 2 mL) followed by centrifugation. The organic phases were combined and passed through a 200 nm PTFE-syringe filter. The solution was concentrated in vacuum (50°C, 100 mbar) followed by distillation of low-boiling fraction at +60°C (0.1 mbar). The diastereomers were separated by fractional crystallization from acetonitrile. Thus, *R,S*-**2a**-$^{15}N_2$ was obtained by crystallization from a small amount of hot MeCN solution (approx. 200 µL) upon cooling to –20°C. The assignment of the absolute configuration was made based on the identity of the aggregate state to that of non-labeled *R,S*-**2a**. *R,R*-**2a**-$^{15}N_2$ (as a mixture with *R,S*-**2a**-$^{15}N_2$ in 9:1 ratio) was recovered from the mother liquor upon concentration of MeCN in vacuum.

(1R,2S)-dibenzyl-1,2-diphenylethane-1,2-diamine-$^{15}N_2$

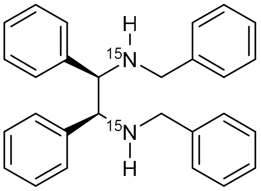

R,S-**2a**-$^{15}N_2$

^{1}H NMR (400 MHz, Acetonitrile-*d*$_3$) δ 7.35 – 7.14 (m, 16H), 7.03 (d, *J* = 6.7 Hz, 4H), 3.77 (s, 2H), 3.50 (d, *J* = 13.5 Hz, 2H), 3.29 (d, *J* = 13.6 Hz, 2H). ^{13}C NMR (101 MHz, Acetonitrile-*d*$_3$) δ 142.3 (d, *J* = 1.9 Hz), 141.7, 129.4, 129.0, 128.9, 128.8, 128.1, 127.5, 68.0 (dd, *J* = 4.5, 3.0 Hz), 51.5 (d, *J* = 4.5 Hz). ^{15}N NMR (41 MHz, Acetonitrile-*d*$_3$) δ 49.8. HRMS (m/z): $[M+H]^+$ calcd. for $C_{28}H_{29}{}^{15}N_2{}^+$, 395.2266; found, 395.2256.

(1R,2R)-dibenzyl-1,2-diphenylethane-1,2-diamine-$^{15}N_2$

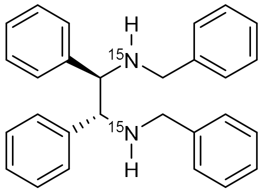

^{1}H NMR (400 MHz, Acetonitrile-*d*$_3$) δ 7.26 (dq, *J* = 16.0, 8.8, 8.1 Hz, 13H), 7.16 – 7.05 (m, 11H), 3.70 (s, 2H), 3.56 (d, *J* = 13.4 Hz, 2H), 3.42 (d, *J* = 13.3 Hz, 2H). ^{13}C NMR (101 MHz, Acetonitrile-*d*$_3$) δ 142.7, 141.9, 129.2, 129.1, 128.9, 128.7, 127.6, 127.5, 69.1 – 68.8 (m), 51.7 (d, *J* = 4.7 Hz). ^{15}N NMR (41 MHz, Acetonitrile-*d*$_3$) δ 49.7. HRMS (m/z): $[M+H]^+$ calcd. for $C_{28}H_{29}{}^{15}N_2{}^+$, 395.2266; found, 395.2259.

1.20. Synthesis of *R,S*-**2a** and *R,R*-**2a** from **1a** by reduction with Zn dust

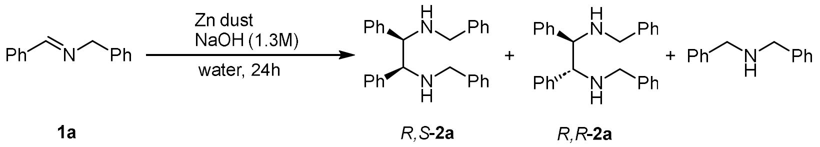

According to the adapted literature procedure,[7] Zn dust (24.9 g, 383 mmol) was added in portions to a stirred mixture of imine **1a** (4.88 g, 25 mmol) in aqueous solution of NaOH (1.3 M, 100 mL). The reaction mixture was stirred at room temperature overnight. The solid was separated by filtration followed by washing with EtOAc. The organic phase was separated, dried over anhydrous Na_2SO_4, and concentrated in vacuum. The residue was triturated with MeCN, the solid was filtered, washed with small amount of MeCN and dried on filter affording *R,S*-**2a**. Yield: 1.04 g. The solution was concentrated in vacuum (+50°C, 100 mbar). Dibenzylamine was distilled of in vacuum (0.03 mbar, 100°C). The residue was purified by flash

column chromatography (basic Al_2O_3, hexane:EtOAc gradient 19:1 ⟶ 9:1). Yield of *R,R*-**2a**: 0.72 g. Combined yield of *R,S*-**2a** and *R,R*-**2a**: 36%.[8]

1.21. A general procedure trialkylamines conversion under anaerobic conditions.

A 4 mL screw-caped glass vial was charged with mpg-CN-8nm-193 (20 mg), R_3N (0.2 mmol), CD_3CN (2 mL) and magnetic stir bar. The mixture was degassed three times using freeze-pump-thaw procedure and refilled with Ar. The mixture was stirred at room temperature under irradiation with blue LED (465 nm, 10±2 mW cm^{-2}) for 24 h. A solution of 1,3,5-trimethoxybenzene (0.1 mL, 0.2 M) was added to the reaction mixture. An aliquot (1 mL) was transferred in an NMR tube. The composition of the mixture was analyzed by ^1H NMR.

Supplementary Discussion 1

In Figure 2, Na-PHI with S_{SA} of approx. 1 $m^2 g^{-1}$, determined from the isotherm of N_2 physisorption, was used. The material with such a low S_{SA} was chosen to study the influence of the material ionic character (instead of its surface area) on its performance in benzylamine oxidation. Note that mpg-CN is a covalent structure. For comparison, we also studied Na-PHI with S_{SA} of 35 $m^2 g^{-1}$. Conversion of benzylamine, yield of **1a** and **2a** were higher for Na-PHI with higher S_{SA} as shown in Supplementary Fig. 9. Corresponding data is given in Supplementary Table 1.

In Supplementary Fig. 10, conversion of benzylamine scales linearly with log(S) (corresponding data is given in Supplementary Table 31). These results suggest that oxidation of benzylamine is a heterogeneous process and occurs on the g-CN surface. Otherwise, it would be independent on surface area, if the reaction occurred in the bulk of the material. More specifically the fitting parameters are summarized in Supplementary Table 32. In the case of Na-PHI, fitting parameters are similar, k = 25-28 and b = 70-74, suggesting that they are independent on the temperature at which tetramerization of benzylamine was conducted. In the case of mpg-CN, conversion of benzylamine is more sensitive to the surface area of the semiconductor (k = 40) compared to Na-PHI (k = 25-28). The fact that the relationship between the conversion of benzylamine and the available surface area of the semiconductor is non-linear suggests contribution of other factors – dependence of enthalpy of H^+ adsorption at g-CN on the degree of the semiconductor photocharging (see section "Results of DFT modelling" in the manuscript).

Supplementary Discussion 2

To check if higher δ values are obtained, when greater excess of benzylamine per carbon nitride mass is used, we conducted photocharging of Na-PHI (5 mg, i.e., the same optical density) in the presence of various benzylamine concentrations. The results are shown in Supplementary Fig. 8. Note that δ value obtained using 0.3 mol L^{-1} benzylamine solution in MeCN might be overestimated due to spontaneous reduction of MV^{2+} $2PF_6^-$ by benzylamine in the dark as indicated in the Supplementary Methods section "Measurement of the number of stored in semiconductor electrons". Overall, δ values are independent of benzylamine concentration in the range 0.01-0.2 mol L^{-1}, i.e., δ is independent on carbon nitride to benzylamine ratio.

According to Bunsen-Roscoe law the amount of converted reagent proportional to the amount absorbed light. Therefore, using thick vials or vials which are not transparent to photons of specific wavelength will give lower δ values. In this work, we used vials that absorb approx. 10% of incident photons at $\lambda > 350$ nm.

Earlier it was reported that saturation of carbon nitride materials with electrons occurs within 2 h of irradiation, i.e., δ reaches its maximum value under given conditions.[9] Taking into account time of a typical photocharging experiment (24 h), during which reaction the mixture received portion of photons approx. 0.2 mol (410 nm, 39 mW cm^{-2}, vial outer surface area 18 cm^2) the apparent quantum yield (AQY) that is defined by the number of electrons stored in carbon nitride to the number of incident photons is approx. 2×10^{-5} (or 0.002%). Kinetic studies suggest that amines have strong affinity to carbon nitride. Therefore, we speculate that their oxidation is fast. Relatively low AQY value indicates that in the typical photocharging experiment, the semiconductor receives large excess of photons. Overall, these analyzes imply that the reported δ values are maximum under the specific conditions, i.e., carbon nitrides are saturated with electrons.

Supplementary Discussion 3

We performed photocharging of Na-PHI at 80°C using benzylamine as electron donor. Quenching with MV^{2+} $2PF_6^-$ was performed at room temperature. The corresponding δ values were plotted vs. Na-PHI mass in Figure 3c and original data was added to Supplementary Table 1. Na-PHI shows systematically lower δ values, when photocharging was conducted at 80°C compared to that at room temperature, which agrees with the results of DFT modelling. Given that sorption of e^-/H^+ is exergonic, while desorption of e^-/H^+ is therefore endergonic, heating of the reaction mixture favors discharging.

Heating facilitates dispersion of Na-PHI, which results in colloidal solution of higher optical density. Therefore, even at low Na-PHI loading (1 mg), absorption of light by the colloidal solution was improved compared to the same amount of Na-PHI photocharged at room temperature. Therefore, comparable δ values were obtained for 1 mg of Na-PHI photocharged at room temperature and at 80°C.

Supplementary Discussion 4

To check if in benzylamine tetramerization experiments conducted at room temperature and at 80°C, substitution of Na in Na-PHI by other cations took place, samples (original mass of Na-PHI 80 mg) after washing with CH_2Cl_2 were dispersed in deionized water (1.5 mL). Na-PHI was separated by centrifugation at 13000 rpm. The liquid phase was decanted and solid was dried in vacuum (+60°C, 20 mbar). Fresh Na-PHI, and recovered samples were characterized by DRUV-vis absorption and FT-IR spectroscopies. DRUV-vis spectra revealed that optical band gap of Na-PHI did not change, although additional states were introduced, which are observed as absorption in the range 470-650 nm (Supplementary Fig. 15a). FT-IR spectra of three materials are similar (Supplementary Fig. 15b). The peak at approx. 985 nm, which is assigned to N-Na vibrations,[10] is also present in three samples suggesting that extensive substitution of Na^+ by NH_4^+ or H^+ did not take place.

Next, we checked if there is any correlation between the amount of Na^+ extracted from Na-PHI and the amount of electrons stored in Na-PHI. Na content in Na-PHI was determined using inductively coupled plasma optical emission spectroscopy (ICP-OES). The dependence of n_{Na+} on n_{e-} is shown in Supplementary Fig. 16 (original data is shown in Supplementary Table 34). Assuming that there is a linear relationship between n_{Na+} and n_{e-}, which is expressed by the function $n_{Na+} = k \times n_{e-} + b$, slopes were determined to be k = 25.3 (reaction conducted at 80°C, R^2 = 0.544) and k = 7.1 (reaction conducted at room temperature, R^2 = 0.999). In other words, storage of one electron in Na-PHI occurs at the expense of approx. 25 and 7 Na^+ cations. To maintain charge balance, Na^+ ions that were extracted from Na-PHI are like to be replaced by NH_4^+ and H^+. While NH_4^+ ions are generated upon coupling of two benzylamine molecules, H^+ might originate from water that is present in MeCN, crystallization water in Na-PHI or water adsorbed on Na-PHI. This process may be described by the equation:

$$Na_xH_y[C_6N_8]_z + 2PhCH_2NH_2 + bH_2O \xrightarrow{h\nu} Na_{x-b}H_{y+b}[C_6N_8]_z(2e_t^-/H^+/NH_4^+) + PhCHNCH_2Ph + bNaOH \quad (8)$$

We speculate that the reason that storage of one electron in Na-PHI requires extraction of more than one Na^+ is better stabilization of the stored electrons in the bulk of the material. Taking into account ordered transport of ions through Na-PHI microchannels,[11] in order for electron and charge-compensating cation (NH_4^+ or H^+) to reach the bulk up to 7 and 25 Na^+ must leave the micropore when photocharging is conducted at room temperature and 80°C, respectively. Overall, such mechanism agrees with the proposed earlier.[11]

Furthermore, the fitting lines intersect X-axis at 1.3 and 2.7 µmol, respectively. In other words, storage of up to 2.7 µmol electrons in Na-PHI should be possible without extracting Na^+. We speculate that such mode of electron storage is possible when occurs exclusively on the outer surface of Na-PHI particles without perturbing the environment of the micropores. Taking into account specific surface area of Na-PHI used to create Supplementary Fig. 16 (1 $m^2\ g^{-1}$), unit cell parameters ($a = b$ = 1.238 nm, c = 0.33 nm),[2] the corresponding areas of the unit cell individual facets (S_{ab} = 1.533 nm^2, $S_{ac} = S_{bc}$ = 0.409 nm^2) and the number of heptazine units per unit cell projected onto the specific cell facet (k = 2, see Supplementary Fig. 2 for crystal structure visualization), the amount of heptazine units on the surface of Na-PHI particles is 8, 2 or 8 µmol g^{-1}, when the surface is terminated by (ac), (ab) or (bc) facets, respectively. Na-PHI loading greater than 1.5-6.2 g (photocharging at 80°C) and 0.74-3.0 g (photocharging at room temperature) would be required to store 1.3-2.7 µmol of electrons and charge-compensating ions exclusively on the outer surface of Na-PHI particles. However, such mode of electron storage is very unlikely to be achieved due

to better stabilization of electrons in the bulk of the material (see a paragraph above). Therefore, it is a theoretical value.

Supplementary Discussion 5

To determine more accurately how long Na-PHI can remain photocharged in air, we conducted two experiments. In both experiments, a mixture of Na-PHI and NH_4COOH in MeCN/H_2O (3:1) was irradiated at 410 nm for 12 h upon stirring. After that the reaction mixture in the first flask was allowed to react with O_2 from air upon stirring. MeCN/H_2O from the second flask was decanted and the residue was dried in vacuum (room temperature, 0.2 mbar) for 30 min and exposed to air. Change of Na-PHI color in both cases is shown in Supplementary Fig. 11.

It is obvious that in the first case, oxidation of Na-PHI occurs fast (within 30 min), which is due to improved mass transport of O_2 to Na-PHI particles. Oxidation of photocharged Na-PHI in the solid state proceeds slower – some areas of Na-PHI film retained its gray metallic sheen even after 4 h.

Flocculation observed in these experiments is due to separation of MeCN/H_2O phases. While these two solvents are immiscible, phase separation occurs in the presence of electrolyte, such as NH_4COOH, which decreases solubility of MeCN in water. Black droplets are rich in water, Na-PHI and NH_4COOH.

Supplementary Discussion 6

A mixture of Na-PHI, NH_4COOH in MeCN/H_2O was photocharged upon irradiation at 410 nm. Within 5 s of mixture exposure to light, it changed color from yellow to dark blue. This color is due to appearance of an additional absorption band stretching from 450 nm to nIR (Supplementary Fig. 7). It should be noted that such short exposure of Na-PHI to light did not affect its intrinsic band gap – a band in the UV-vis range of the electromagnetic spectrum with the onset of absorption at approx. 450 nm is observed in pristine Na-PHI and Na-PHI exposed to light for 5 s.

Extended irradiation of the mixture resulted in further color change to dark gray. This color transition is due to broadening of the absorption peak at approx. 650 nm – the resultant material absorbs all photons in the UV-nIR range.

Supplementary Discussion 7

Considering the specific surface area of g-CN materials used in the photocharging experiments and the obtained corresponding δ values, we calculated the ratio of heptazine units per electron. In the case of mpg-CN with specific surface area in the range 84-204 $m^2\ g^{-1}$, statistically one electron is stored per 1-7 heptazine units (electron donor Et_3N) or 2-9 heptazine units (electron donor $^nBu_3N$, Supplementary Table 33). These results do not exclude the possibility that in mpg-CN electrons and charge-compensating cations are stored exclusively on the material surface. However, in case of Na-PHI due to its lower S_{SA} of only 1 $m^2\ g^{-1}$ and higher δ values compared to mpg-CN, up to 167 or 111 electrons (Et_3N and $^nBu_3N$ electron donors respectively) must be stored in a single heptazine unit. Assuming full reduction of a heptazine unit, i.e., disintegration of the material with the formation of three CH_4 and four NH_3 molecules, this process would allow for storage of only 24 electrons in the products of heptazine unit reduction. Therefore, in poly(heptazine imides), electrons and charge compensating cations, must be stored in the micropores. The surface of micropores on the interface with the reaction medium is composed exclusively of (ac) and (bc) crystal cell facets – both are edge planes.

On the other hand, assuming transport of electrons into the bulk of materials accompanied by intercalation of charge compensating cations between the layers, one electron is stored per 14-30 heptazine units. In principle, such a possibility could not be excluded for both types of g-CN materials. However, due to microporous structure ion transport into the bulk of Na-PHI should be more facile compared to mpg-CN.[11]

Taking into account results published earlier and general pathway of tertiary amines oxidation,[12,13,14] the mechanism of aza-pinacol coupling implies formation of the iminium cation (Figure 7). A photocharged mpg-CN with charge-compensating iminium cations, g-CN($2e_t^-/H^+/Im^+$), is a possible intermediate. However, given high reactivity of the iminium cation toward water, which is unavoidably present in MeCN and/or adsorbed on the surface of carbon nitrides, it is converted into dialkylamine and aldehyde. These two species were detected by ^1H NMR, while the released H^+ becomes the charge-compensating ion instead of the iminium cation for the stored electron.

Supplementary Discussion 8

MV^{2+} $2PF_6^-$ in MeCN undergoes electrochemically reversible one-electron reduction to the radical cation, $MV^{\bullet+}$ PF_6^-, at the potential –0.83 V vs. Fc^+/Fc followed by electrochemically reversible one-electron reduction to the dimethylbipyridinylidene at the potential –1.24 V vs. Fc^+/Fc (Supplementary Fig. 6). Both processes are electrochemically reversible because peak separations in both cases are approx. 60 mV.[15]

Tertiary amines, Et_3N and $^nBu_3N$, undergo electrochemically and chemically irreversible one-electron oxidation to the corresponding radical cations at the potentials +0.34 and +0.30 V vs. Fc^+/Fc respectively. Because the reduction peak is absent in the CV curve upon scanning back from positive to negative potential, these processes are electrochemically irreversible. In addition, the radical cations of tertiary amines are short-lived species and undergo further spontaneous transformations, such as cleavage of (sp^3)C-H bond next to nitrogen atom, with the formation of the corresponding iminium cation.[16] The lifetime of the tertiary amine radical cation is significantly shorter compared to the duration of the CV experiment. In other words, at the scan rate 50 mV s^{-1} used to determine oxidation potentials of the tertiary amines, it is impossible to detect electron transfer from the working electrode to the radical cation, because the latter is decomposed and therefore absent in the proximity of the electrode.

Supplementary Discussion 9

Atomic charges in PHIs. Having a closer look at the charges of Na-PHI gives further insights. They were obtained using DDEC6 as implemented in Chargemol, with the cube-files calculated via CP2K as described in the manuscript, although an increased cutoff of 1000 Ry has been used. This is necessary for an appropriate assignment of the electrons. To have the charges in an experimental feasible and computational accurate manner only two decimals were evaluated. The Supplementary Table 24 and 25 give the average charge in [e] for all atoms of each element in the structure, with its standard deviation, if applicable. Overall, the charges of the elements do not drastically vary with changing system size. Although a slight increase in positive charge by circa 0.02 e is observable for carbon and sodium from 2 to 20 heptazine units. This is accompanied by an increase of negative charge on the bridging nitrogen that forms a covalent bond with a hydrogen during the adsorption process.

In general, the nitrogen atoms on the outer part of the heptazine imide cumulate most of the negative charge, which is ascribable to the charge transfer with sodium or other interacting atoms/molecules, but also to their lone pair. Charges on individual atoms in PHIs composed of 20 heptazine units are summarized in Supplementary Table 26 (Na-PHI) and Supplementary Table 27 (H-PHI). Assignment of labels to specific atoms in PHI is given in Supplementary Fig. 17. The trends are identical in the smaller systems, deviations are only small (in the range of 0.02e compared to 2 heptazine PHIs).

When Na-PHI without solvent (Supplementary Table 27) and with solvent (Supplementary Table 28) are compared, charges vary in second decimal due to interaction with the solvent, e.g. hydrogen bonding. Overall influence is of quantitative nature and rather small.

Na-backbone distances in PHIs. Average distance of sodium with CN-Backbone increases when adding water (Supplementary Table 29), but the actual values are again highly dependent on the position and orientation of the explicit solvent. There is a shift of the sodium towards the pore center, as one can derive from the Na-N3 distance (Supplementary Fig. 17). With water molecules solvating the ion the higher distance is sterically favorable.

Adsorption energies in solvated PHIs. PHI systems with each sodium ion explicitly solvated by 5 water molecules[11], were investigated regarding the adsorption energies of H^+/NH_4^+. These energies are shown in Supplementary Table 19. There is no noticeable trend, and the energies are rather dominated by the interaction with the solvent. These interactions are strongly influenced by small differences in the orientation and position of the molecules which result from the geometric optimization of the system. Omitting the solvent reveals more information about the interaction of the adsorbate with the adsorbent in regard of differently charged systems, as can be seen in Fig. 6.

Supplementary Discussion 10

The results of kinetic studies are summarized in the following points:

1. At the beginning of the reaction, the ratio between *R*,*S*-**2a** and *R*,*R*-**2a** is > 1, which is in agreement with the experiments conducted in batch (Supplementary Fig. 13). Due to *R*,*S*-**2a** low solubility in MeCN, the ratio between *R*,*S*- and *R*,*R*-**2a** becomes noticeably smaller than unity when imine **1a** conversion reaches 72% (reaction mixture irradiation for 15 h). After kinetic study was completed, the setup was disassembled. Crystals of *R*,*S*-**2a** were observed to deposit on the cooler. Therefore, the measured by ^{1}H NMR combined yield of the coupling product is lower than the actual.

2. Before the irradiation of the reaction mixture was initiated, 65% of the initial amount of triethylamine was adsorbed on the surface of mpg-CN. Given electron-deficient character of heptazine-based carbon nitride, such results are not surprising. We speculate that the driving force for sorption of electron-rich Et_3N on the surface of mpg-CN is the formation of charge-transfer complex.

3. Although conversion of Et_3N reached 95% after 43 h, the yield of Et_2NH was only 18% suggesting that large fraction remains adsorbed on the surface of mpg-CN.

4. When imine **1a** conversion is < 30%, δ is relatively low – electrons are not accumulated at the significant extent in mpg-CN. However, photocharging of mpg-CN is accelerated when imine **1a** conversion is > 70% (Supplementary Fig. 14).

Supplementary Tables

Supplementary Table 1. Screening of conditions of benzylamine tetramerization by semiconductors and Ir(ppy)$_2$(dtbbpy)PF$_6$.

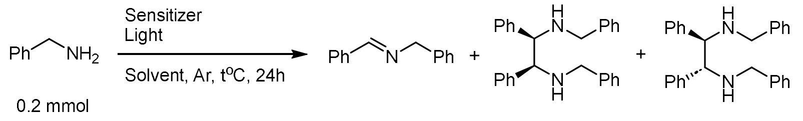

Entry	Sensitizer	Mass, mg	t,°C	Conversion of benzylamine,[a] %	Yield of **1a**,[a] %	Combined yield of *R,S*-**2a** and *R,R*-**2a**,[a],[b] %
1	mpg-CN-8nm-193[c]	20	25±5	30	8	3 (1:0.88)
2	mpg-CN-8nm-193[d]	20	25±5	59	2	31 (1:0.86)
3	mpg-CN-8nm-193[d]	20	80	62±0.4[f]	2±0.5[f]	40±0.5 (1:0.86) [f]
4	mpg-CN-8nm-193[d]	40	80	76	4	48 (1:0.85)
5	mpg-CN-8nm-193[d]	80	80	85	6	58 (1:0.83)
6	mpg-CN-8nm-193[d]	160	80	99	10	58 (1:0.80)
7	mpg-CN-8nm-193[d]	20	80	62	1	40 (1:0.85)
8	Na-PHI[d],[e]	5	25±5	37	8	4 (1:1)
9	Na-PHI[d],[e]	10	25±5	52	8	12 (1:0.91)
10	Na-PHI[d],[e]	20	25±5	76	9	25 (1:0.84)
11	Na-PHI[d],[e]	5	80	54	6	10 (1:0.88)
12	Na-PHI[d],[e]	10	80	52	5	21 (1:0.87)
13	Na-PHI[d],[e]	20	80	69±3[f]	5±0.5[f]	35±2 (1:0.84±0.02)[f]
14	Na-PHI[d],[e]	40	80	75	6	41 (1:0.81)
15	Na-PHI[d],[e]	80	80	90	9	46 (1:0.81)
16	Na-PHI[d],[e]	120	80	92	11	51 (1:0.82)
17	Na-PHI[d],[g]	5.8	25±5	18	3	0.4 (1:1)
18	Na-PHI[d],[g]	10.2	25±5	19	4	1 (1:2)
19	Na-PHI[d],[g]	21.3	25±5	21	5	2 (1:0.6)
20	Na-PHI[d],[g]	40	25±5	3	7	6 (1:1)
21	Na-PHI[d],[g]	80	25±5	43	8	14 (1:1.06)
22	Na-PHI[d],[g]	120	25±5	47	9	17 (1:0.95)
23	Na-PHI[d],[g]	160	25±5	54	10	23 (1:1)
24	Na-PHI[d],[g]	5.3	80	13	3	1 (1:0.5)
25	Na-PHI[d],[g]	10	80	15	4	1 (1:0.75)
26	Na-PHI[d],[g]	21.2	80	30	5	5 (1:0.92)
27	Na-PHI[d],[g]	40	80	36	6	10 (1:0.92)
28	Na-PHI[d],[g]	80	80	43	8	19 (1:0.88)
29	Na-PHI[d],[g]	120	80	51	10	23 (1:0.89)
30	Na-PHI[d],[g]	160	80	61	12	26 (1:0.82)
31	H-PHI[d]	20	25±5	35	11	8 (1:1)

32	CdS[d]	20	25±5	75	0	20 (~ 1:1)
33	CdS[d]	20	80	74	0	14 (~ 1:1)
34	$Ir(ppy)_2(dtbbpy)PF_6$[d]	3.7 (2 mol. %)	25±5	39	10	10 (1:0.76)
35	$Ir(ppy)_3$[d]	2.6 (2 mol. %)	25±5	34	11	1 (1:0.50)
36	TiO2 (anatase < 25 nm)[h]	20	25±5	48	0	29 (1:0.82)
37	WO_3[d]	20	25±5	30	13	0 (–)
38	WO_3[d]	20	80	27	13	0 (–)

[a] determined from ^{1}H NMR spectra using 1,3,5-trimethoxybenzene as internal standard.

[b] ratio between *R,S*-**2a** and *R,R*-**2a** isomers is given in parentheses.

[c] Light 465 nm, 10±2 mW cm^{-2}.

[d] Light 410 nm, 39±8 mW cm^{-2}.

[e] Na-PHI S_{SA} = 35 m^2 g^{-1}.

[f] average±st.deviation, n = 3.

[g] Na-PHI S_{SA} = 1 m^2 g^{-1}.

[h] Light 365nm, 16±4 mW cm^{-2}.

Supplementary Table 2. Control experiments of benzylamine tetramerization by mpg-CN-8nm-193.

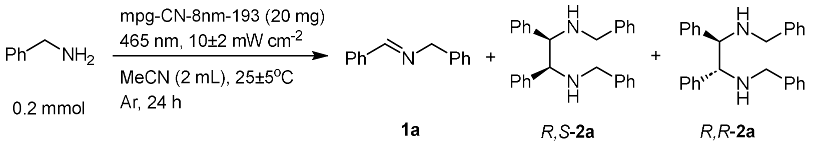

Entry	Deviation from standard conditions	Conversion of benzylamine,[a] %	Yield of **1a**,[a] %	Combined yield of *R,S*-**2a** and *R,R*-**2a**,[b] %
1	None	30	8	3 (1:0.88)
2	Without degassing	47±5[c]	28±2[c]	< 1 (~ 1:1)
3	Without degassing, water (10 equiv.) was added	48	28	< 1 (~ 1:1)
4	In dark	25	7	0 (–)
5	Without mpg-CN	39	4	0 (–)

[a] determined from ^{1}H NMR spectra using 1,3,5-trimethoxybenzene as internal standard.

[b] ratio between *R,S*-**2a** and *R,R*-**2a** isomers is given in parentheses.

[c] average±std., n = 2.

Supplementary Table 3. Half-peak reduction and oxidation potentials.

Entry	Compound	$E_{p1/2}$, V vs. Fc^+/Fc (reduction)	$E_{p1/2}$,[a] V vs. SCE (reduction)	$E_{p1/2}$, V vs. Fc^+/Fc (oxidation)	$E_{p1/2}$,[a] V vs. SCE (oxidation)
1	MV_2^+ $2PF_6^-$	–0.83	–0.45	–	–
2	Et_3N	–	–	+0.34	+0.72
3	$^nBu_3N$	–	–	+0.30	+0.68
4	$PhCH_2NH_2$	–	–	+0.78	+1.16

[a] Half-peak redox potentials vs. SCE was calculated according to equation:

$$E_{p1/2(SCE)} = E_{p1/2(Fc+/Fc)} + 0.38 \quad (9)$$

where $E_{p1/2(Fc+/Fc)}$ – half-peak redox potential measured by CV, V vs. Fc^+/Fc. Conversion constant was taken from reference [17].

Supplementary Table 4. Summary of δ values for different semiconductors.

Entry	Semiconductor	S_{SA}, $m^2 g^{-1}$	*m*, mg	SED	δ, μmol g^{-1}
1	Na-PHI	1	1.057	$PhCH_2NH_2$	380±108
2	Na-PHI	1	5	$PhCH_2NH_2$	644
3	Na-PHI	1	10	$PhCH_2NH_2$	470
4	Na-PHI	1	20	$PhCH_2NH_2$	353
5	Na-PHI	1	40	$PhCH_2NH_2$	282
6	H-PHI	12	1.116	$PhCH_2NH_2$	304
7	H-PHI	12	5	$PhCH_2NH_2$	146
8	H-PHI	12	10	$PhCH_2NH_2$	104
9	H-PHI	12	20	$PhCH_2NH_2$	39
10	H-PHI	12	40	$PhCH_2NH_2$	31
11	mpg-CN-8nm-193	193	1.07	$PhCH_2NH_2$	289
12	mpg-CN-8nm-193	193	5	$PhCH_2NH_2$	121
13	mpg-CN-8nm-193	193	10	$PhCH_2NH_2$	116
14	mpg-CN-6nm-204	204	20	$PhCH_2NH_2$	56
15	mpg-CN-8nm-193	193	20	$PhCH_2NH_2$	48
16	mpg-CN-17nm-171	171	20	$PhCH_2NH_2$	43
17	mpg-CN-7nm-149	149	20	$PhCH_2NH_2$	91
18	mpg-CN-7nm-84	84	20	$PhCH_2NH_2$	53
19	mpg-CN-8nm-193	193	40	$PhCH_2NH_2$	42
20	K-PHI	89	5	$PhCH_2NH_2$	701±46[4]
21	mpg-CN	180	10	$PhCH_2NH_2$	43±5[4]
22	mpg-CN-6nm-204	204	20	Et_3N	252±16
23	mpg-CN-8nm-193	193	20	Et_3N	250
24	mpg-CN-8nm-193	193	20	$Et_3N + NH_4PF_6$[a]	68
25	mpg-CN-17nm-171	171	20	Et_3N	241
26	mpg-CN-7nm-149	149	20	Et_3N	258
27	mpg-CN-7nm-84	84	20	Et_3N	269
28	mpg-CN-6nm-204	204	20	$^{n}Bu_3N$	187
29	mpg-CN-8nm-193	193	20	$^{n}Bu_3N$	181
30	mpg-CN-8nm-193	193	20	$^{n}Bu_3N + NH_4PF_6$[a]	47
31	mpg-CN-17nm-171	171	20	$^{n}Bu_3N$	164
32	mpg-CN-7nm-149	149	20	$^{n}Bu_3N$	187
33	mpg-CN-7nm-84	84	20	$^{n}Bu_3N$	167
34	Na-PHI	1	20	Et_3N	349
35	Na-PHI	1	20	$Et_3N + NH_4PF_6$[a]	423
36	Na-PHI	1	20	$^{n}Bu_3N$	243
37	Na-PHI	1	20	$^{n}Bu_3N + NH_4PF_6$[a]	338
38	Na-PHI[b]	1	1	$PhCH_2NH_2$	397
39	Na-PHI[b]	1	5	$PhCH_2NH_2$	445
40	Na-PHI[b]	1	10	$PhCH_2NH_2$	202
41	Na-PHI[b]	1	20	$PhCH_2NH_2$	106
42	Na-PHI[b]	1	40	$PhCH_2NH_2$	92±1

[a] NH_4PF_6 (0.4 mmol).

[b] Photocharging was conducted at 80°C for 24 h followed by reaction mixture quenching with MV^{2+} at room temperature.

Supplementary Table 5. *Aza*-pinacol coupling of imine **1a** by mpg-CN-6nm-204. Screening of SEDs.

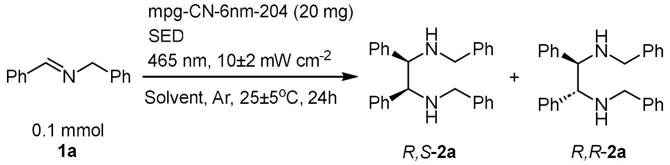

Entry	SED	Solvent	Conversion of **1a**,[a] %	Yield of *R,S*-**2a**,[a] mmol	Yield of *R,R*-**2a**,[a] mmol	Combined yield of *R,S*-**2a** and *R,R*-**2a**,[b] %
1	$^{i}Pr_2NEt$ (0.2 mmol, 35 µL)	MeCN (2 mL)	100	0.026	0.0065	65 (1:0.25)
2	$^{n}Bu_3N$ (0.2 mmol, 48 µL)	MeCN (2 mL)	99	0.024	0.0028	54 (1:0.12)
3	Et_3N (0.2 mmol, 28 µL)	MeCN (2 mL)	92	0.017	0.012	58 (1:0.69)
4	Me_3N in EtOH (0.2 mmol, 48 µL, ~ 4.2 M)	MeCN (2 mL)	98	0.0072	0.0051	25 (1:0.70)
5	NH_4COOH (0.2 mmol, 13 mg)	MeCN (2 mL)	20	0.0061	0.0012	15 (1:0.19)
6	NH_4COOH (0.2 mmol, 13 mg)	MeCN (1.5 mL) + H_2O (0.5 mL)	47	0.00032	0.00032	1 (1:1.00)

[a] Determined from ^{1}H NMR spectra using 1,3,5-trimethoxybenzene as internal standard.

[b] Ratio between *R,S*-**2a** and *R,R*-**2a** isomers is given in parentheses.

Supplementary Table 6. *Aza*-pinacol coupling of imine **1a** by mpg-CN-8nm-193. Screening of SEDs.

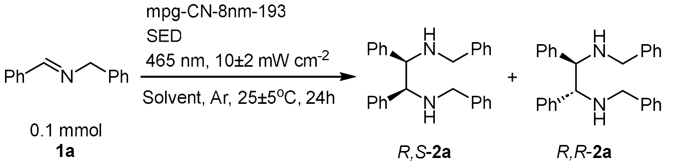

Entry	Mpg-CN-8nm-193 mass, mg	SED	Solvent	Conversion of **1a**,[a] %	Yield of *R,S*-**2a**,[a] mmol	Yield of *R,R*-**2a**,[a] mmol	Combined yield of *R,S*-**2a** and *R,R*-**2a**,[b] %
1	5 mg	${}^{i}Pr_2NEt$ (0.2 mmol, 35 µL)	MeCN (2 mL)	94	0.025	0.013	77 (1:0.54)
2	10 mg	${}^{i}Pr_2NEt$ (0.2 mmol, 35 µL)	MeCN (2 mL)	99	0.026	0.013	79 (1:0.53)
3	20	${}^{i}Pr_2NEt$ (0.2 mmol, 35 µL)	MeCN (2 mL)	100	0.026	0.0075	68 (1:0.29)
4	20	${}^{n}Bu_3N$ (0.2 mmol, 48 µL)	MeCN (2 mL)	99	0.025	0.0084	66 (1:0.34)
5	20	Et_3N (0.2 mmol, 28 µL)	MeCN (2 mL)	98	0.018	0.014	65 (1:0.77)
6	20	Me_3N in EtOH (0.2 mmol, 48 µL, ~ 4.2 M)	MeCN (2 mL)	99	0.010	0.0099	40 (1:0.99)
7	20	NH_4COOH (0.2 mmol, 13 mg)	MeCN (2 mL)	26	0.0050	0.0030	16 (1:0.60)
8	20	NH_4COOH (0.2 mmol, 13 mg)	MeCN (1.5 mL) + H_2O (0.5 mL)	44	0.0017	0.00049	4 (1:0.29)

[a] Determined from ^{1}H NMR spectra using 1,3,5-trimethoxybenzene as internal standard.

[b] Ratio between *R,S*-**2a** and *R,R*-**2a** isomers is given in parentheses.

Supplementary Table 7. *Aza*-pinacol coupling of imine **1a** by mpg-CN-17nm-171. Screening of SEDs.

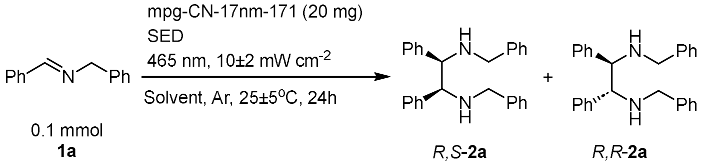

Entry	SED	Solvent	Conversion of **1a**,[a] %	Yield of *R,S*-**2a**,[a] mmol	Yield of *R,R*-**2a**,[a] mmol	Combined yield of *R,S*-**2a** and *R,R*-**2a**,[b] %
1	$^iPr_2NEt$ (0.2 mmol, 35 µL)	MeCN (2 mL)	100	0.025	0.0095	69 (1:0.38)
2	$^nBu_3N$ (0.2 mmol, 48 µL)	MeCN (2 mL)	99	0.025	0.0092	69 (1:0.36)
3	Et_3N (0.2 mmol, 28 µL)	MeCN (2 mL)	99	0.019	0.017	72 (1:0.91)
4	Me_3N in EtOH (0.2 mmol, 48 µL, ~ 4.2 M)	MeCN (2 mL)	99	0.011	0.0074	38 (1:0.64)
5	NH_4COOH (0.2 mmol, 13 mg)	MeCN (2 mL)	28	0.0063	0.0045	22 (1:0.72)
6	NH_4COOH (0.2 mmol, 13 mg)	MeCN (1.5 mL) + H_2O (0.5 mL)	45	0.0013	0.0013	5 (1:1.00)

[a] Determined from ^1H NMR spectra using 1,3,5-trimethoxybenzene as internal standard.

[b] Ratio between *R,S*-**2a** and *R,R*-**2a** isomers is given in parentheses.

Supplementary Table 8. *Aza*-pinacol coupling of imine **1a** by CdS. Screening of SEDs.

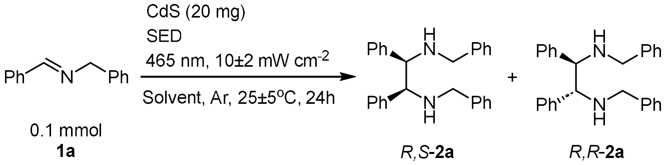

Entry	SED	Solvent	Conversion of **1a**,[a] %	Yield of *R,S*-**2a**,[a] mmol	Yield of *R,R*-**2a**,[a] mmol	Combined yield of *R,S*-**2a** and *R,R*-**2a**,[b] %
1	$^{i}Pr_2NEt$ (0.2 mmol, 35 µL)	MeCN (2 mL)	24	0.0057	0.0038	19 (1:0.65)
2	$^{n}Bu_3N$ (0.2 mmol, 48 µL)	MeCN (2 mL)	40	0.0085	0.0070	31 (1:0.83)
3	Et_3N (0.2 mmol, 28 µL)	MeCN (2 mL)	45	0.0077	0.0056	27 (1:0.73)
4	Me_3N in EtOH (0.2 mmol, 48 µL, ~ 4.2 M)	MeCN (2 mL)	14	0.0027	0.0021	10 (1:0.8)
5	NH_4COOH (0.2 mmol, 13 mg)	MeCN (2 mL)	98	0.016	0.012	56 (1:0.77)
6	NH_4COOH (0.2 mmol, 13 mg)	MeCN (1.5 mL) + H_2O (0.5 mL)	100	0.023	0.018	81 (1:0.79)

[a] Determined from ^{1}H NMR spectra using 1,3,5-trimethoxybenzene as internal standard.

[b] Ratio between *R,S*-**2a** and *R,R*-**2a** isomers is given in parentheses.

Supplementary Table 9. *Aza*-pinacol coupling of imine **1a** by Ir(ppy)$_2$(dtbbpy)PF$_6$. Screening of SEDs.

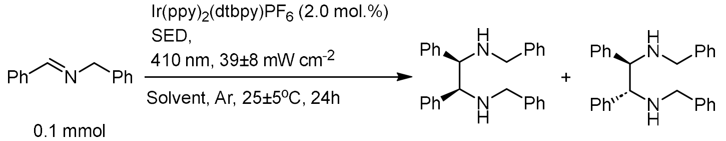

0.1 mmol **1a** | *R,S*-**2a** | *R,R*-**2a**

Entry	SED	Solvent	Conversion of **1a**,[a] %	Yield of *R,R*-**2a**,[a] mmol	Yield of *R,R*-**2a**,[a] mmol	Combined yield of *R,S*-**2a** and *R,R*-**2a**,[b] %
1	$^iPr_2NEt$ (0.2 mmol, 35 µL)	MeCN (2 mL)	99	0.026	0.019	90 (1:0.74)
2	NH_4COOH (0.2 mmol, 13 mg)	MeCN (2 mL)	3	0.00099	0.00055	3 (1:0.56)
3	$^nBu_3N$ (0.2 mmol, 48 µL)	MeCN (2 mL)	100	0.022	0.017	66 (1:0.81)
4	Et_3N (0.2 mmol, 28 µL)	MeCN (2 mL)	99	0.023	0.019	65 (1:0.83)
5	NH_4COOH (0.2 mmol, 13 mg)	MeCN (1.5 mL) + H_2O (0.5 mL)	65	0.00069	0.00069	3 (1:1.00)
6	Me_3N in EtOH (0.2 mmol, 48 µL, ~ 4.2 M)	MeCN (2 mL)	99	0.017	0.0077	50 (1:0.44)
7[c]	NH_4COOH (0.2 mmol, 13 mg)	MeCN (1.5 mL) + H_2O (0.5 mL)	48	0.00049	0.00049	2 (1:1.00)

[a] Determined from ^{1}H NMR spectra using 1,3,5-trimethoxybenzene as internal standard.

[b] Ratio between *R,S*-**2a** and *R,R*-**2a** isomers is given in parentheses.

[c] Without photocatalyst.

Supplementary Table 10. *Aza*-pinacol coupling of imine **1a** by Na-PHI. Screening of SEDs.

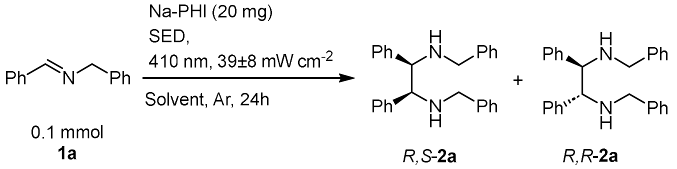

Entry	SED	Solvent	t, °C	Conversion of **1a**,[a] %	Yield of *R,S*-**2a**,[a] mmol	Yield of *R,R*-**2a**,[a] mmol	Combined yield of *R,S*-**2a** and *R,R*-**2a**,[b] %
1	$^{i}Pr_2NEt$ (0.2 mmol, 35 µL)	MeCN (2 mL)	25±5	46	0.012	0.011	46 (1:0.84)
2	$^{n}Bu_3N$ (0.2 mmol, 48 µL)	MeCN (2 mL)	25±5	45	0.013	0.0098	45 (1:0.78)
3	Et_3N (0.2 mmol, 28 µL)	MeCN (2 mL)	25±5	35	0.0073	0.0057	26 (1:0.78)
4	Me_3N in EtOH (0.2 mmol, 48 µL, ~ 4.2 M)	MeCN (2 mL)	25±5	34	0.0066	0.0036	20 (1:0.55)
5	NH_4COOH (0.2 mmol, 13 mg)	MeCN (2 mL)	25±5	83	0.017	0.014	61 (1:0.83)
6	NH_4COOH (0.2 mmol, 13 mg)	MeCN (1.5 mL) + H_2O (0.5 mL)	25±5	61	0.011	0.0055	33 (1:0.50)
7	NH_4COOH (0.2 mmol, 13 mg)	MeCN (2 mL)	80	98	0.016	0.0091	51 (1:0.56)
8	Triethanolamine (0.2 mmol, 26 µL)	MeCN (2 mL)	80	0	0	0	0 (–)
9	$^{n}Bu_3N$ (0.2 mmol, 48 µL)	MeCN (2 mL)	80	64	0.019	0.013	64 (1:0.68)
10	Et_3N (0.1 mmol, 14 µL)	MeCN (2 mL)	80	57	0.014	0.0079	44 (1:0.55)
11	Et_3N (0.2 mmol, 28 µL)	MeCN (2 mL)	80	76	0.022	0.013	70 (1:0.60)
12	Et_3N (0.3 mmol, 42 µL)	MeCN (2 mL)	80	61	0.015	0.0083	47 (1:0.55)
13	$^{i}Pr_2NEt$ (0.1 mmol, 35 µL)	MeCN (2 mL)	80	51	0.016	0.0087	49 (1:0.56)
14	$^{i}Pr_2NEt$ (0.2 mmol, 35 µL)	MeCN (2 mL)	80	72	0.023	0.013	72 (1:0.56)
15	$^{i}Pr_2NEt$ (0.3 mmol, 35 µL)	MeCN (2 mL)	80	68	0.019	0.0078	54 (1:0.41)

16	$^{i}Pr_2NEt$ (0.4 mmol, 35 μL)	MeCN (2 mL)	80	82	0.023	0.012	69 (1:0.53)
17	$^{i}Pr_2NEt$ (0.5 mmol, 35 μL)	MeCN (2 mL)	80	61	0.017	0.0092	52 (1:0.54)
18	H_2 (~1 bar)[c]	MeCN (2 mL)	80	1	0	0	0 (–)
19	D(+)-glucose (0.1 mmol, 18 mg)	MeCN (2 mL)	80	0	0	0	0 (–)
20	Na_2SO_3 (0.2 mmol, 25 mg)	$^{i}PrOH$ (2 mL)	80	27	0.0082	0.0053	27 (1:0.64)
21	Na_2SO_3 (0.2 mmol, 25 mg)	H_2O (2 mL)	80	4	0.0011	0.001	4 (1:0.90)
22	–	$^{i}PrOH$ (2 mL)	80	19	0	0	0 (–)
23	–	$^{i}PrOH$[d] (2 mL)	80	11	0.004	0.001	9 (1:0.22)
24	Na_2SO_3 (0.2 mmol, 25 mg)	MeCN (2 mL)	80	9	0	0	0 (–)

[a] Determined from ^{1}H NMR spectra using 1,3,5-trimethoxybenzene as internal standard.

[b] Ratio between *R,S*-**2a** and *R,R*-**2a** isomers is given in parentheses.

[c] The reactor headspace was refilled with H_2 after freeze-pump-thaw procedure. A balloon with H_2 (approx. 1 bar, absolute pressure) remained connected to the reaction mixture during the experiment.

[d] [EMIM]OTf (0.1 mmol, 1 equiv.)

Supplementary Table 11. *Aza*-pinacol coupling of imine **1a** by H-PHI. Screening of SEDs.

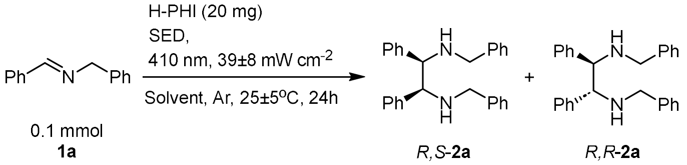

Entry	SED	Solvent	Conversion of **1a**,[a] %	Yield of *R,S*-**2a**,[a] mmol	Yield of *R,R*-**2a**,[a] mmol	Combined yield of *R,S*-**2a** and *R,R*-**2a**,[b] %
1	$^{i}Pr_2NEt$ (0.2 mmol, 35 µL)	MeCN (2 mL)	66	0.0159	0.0076	47 (1:0.48)
2	$^{n}Bu_3N$ (0.2 mmol, 48 µL)	MeCN (2 mL)	99	0.0166	0.0108	55 (1:0.65)
3	Et_3N (0.2 mmol, 28 µL)	MeCN (2 mL)	88	0.0142	0.0085	45 (1:0.60)
4	NH_4COOH (0.2 mmol, 13 mg)	MeCN (2 mL)	98	0.0231	0.0141	74 (1:0.61)
5	NH_4COOH (0.2 mmol, 13 mg)	MeCN (1.5 mL) + H_2O (0.5 mL)	80	0.0109	0.0052	32 (1:0.47)

[a] Determined from ^{1}H NMR spectra using 1,3,5-trimethoxybenzene as internal standard.

[b] Ratio between *R,S*-**2a** and *R,R*-**2a** isomers is given in parentheses.

Supplementary Table 12. Control experiments in *aza*-pinacol coupling of imine **1a** by mpg-CN-8nm-193.

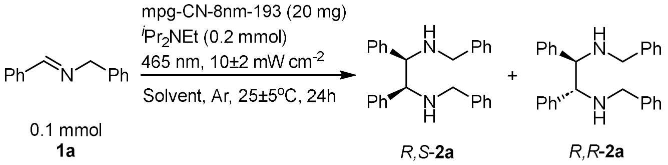

Entry	Deviation from standard conditions	Conversion of **1a**,[a] %	Yield of *R,S*-**2a**,[a] mmol	Yield of *R,R*-**2a**,[a] mmol	Combined yield of *R,S*-**2a** and *R,R*-**2a**,[b] %
1	None	100	0.026	0.0075	68 (1:0.29)
2	Without degassing	99	0.026	0.0074	67 (1:0.28)
3	Without degassing, water (10 equiv.) was added	97	0.026	0.012	77 (1:0.46)
4	In dark	0	0	0	0 (–)
5	Without mpg-CN	0	0	0	0 (–)
6	Without ^{i}Pr$_2$NEt	0	0	0	0 (–)

[a] Determined from ^{1}H NMR spectra using 1,3,5-trimethoxybenzene as internal standard.

[b] Ratio between *R,S*-**2a** and *R,R*-**2a** isomers is given in parentheses.

Supplementary Table 13. Study of Et_3N oxidation in *aza*-pinacol coupling.

Entry	Photocatalyst	Light	Et_2NH yield,[a] mmol	**1a** conversion,[a] mmol	*R,S*– and *R,R*–**2a** yield,[a] mmol
1	mpg-CN-6nm-204	465 nm (10±2 mW cm^{-2})	0.060	0.093	0.0132
2	mpg-CN-8nm-193	465 nm (10±2 mW cm^{-2})	0.059	0.096	0.0133
3	mpg-CN-17nm-171	465 nm (10±2 mW cm^{-2})	0.060	0.096	0.0146
4	CdS	465 nm (10±2 mW cm^{-2})	0.021	0.054	0.0075
5	Na-PHI	410 nm (39±8 mW cm^{-2})	0.032	0.053	0.0087
6	$Ir(ppy)_2(dtbbpy)PF_6$ 2 mol. %	410 nm (39±8 mW cm^{-2})	0.074	0.100	0.0179
7	TiO_2 (anatase < 25 nm)	365 nm (16±4 mW cm^{-2})	0.030	0.100	0.0137

[a] Determined from ^1H NMR spectra using 1,3,5-trimethoxybenzene as internal standard.

Supplementary Table 14. Study of Et_3N and $^nBu_3N$ oxidation in *aza*-pinacol coupling.

Entry	Sensitizer	S_{SA}, $m^2\ g^{-1}$	Light	SED	Et_2NH yield,[a] μmol	$^nPrC(O)H$ yield,[a] μmol
1	mpg-CN-5nm-14	14	465 nm (10±2 mW cm^{-2})	Et_3N	19.3	–
2	mpg-CN-5nm-28	28	465 nm (10±2 mW cm^{-2})	Et_3N	22.8	–
3	mpg-CN-5nm-61	61	465 nm (10±2 mW cm^{-2})	Et_3N	20.5	–
4	mpg-CN5nm-33	33	465 nm (10±2 mW cm^{-2})	Et_3N	24.1	–
5	mpg-CN-7nm-84	84	465 nm (10±2 mW cm^{-2})	Et_3N	31.8	–
6	mpg-CN-7nm-70	70	465 nm (10±2 mW cm^{-2})	Et_3N	24.4	–
7	mpg-CN-5nm-141	141	465 nm (10±2 mW cm^{-2})	Et_3N	29.1	–
8	mpg-CN-7nm-149	149	465 nm (10±2 mW cm^{-2})	Et_3N	33.3	–
9	mpg-CN-17nm-171	171	465 nm (10±2 mW cm^{-2})	Et_3N	24.5±0.2	–
10	mpg-CN-8nm-193	193	465 nm (10±2 mW cm^{-2})	Et_3N	27.1±0.1	–
11	mpg-CN-8nm-193	193	465 nm (10±2 mW cm^{-2})	Et_3N + Water (1 equiv.)	22.1	–
12	mpg-CN-8nm-193	193	465 nm (10±2 mW cm^{-2})	Et_3N + Water (5 equiv.)	21.2	–
13	mpg-CN-8nm-193	193	465 nm (10±2 mW cm^{-2})	Et_3N + Water (10 equiv.)	20.3	–

14	mpg-CN-8nm-193	193	465 nm (10±2 mW cm^{-2})	Et_3N[b] (0.1 mmol)	25	
15	mpg-CN-8nm-193	193	465 nm (10±2 mW cm^{-2})	Et_3N[c] (0.05 mmol)	21	
16	mpg-CN-8nm-193	193	465 nm (10±2 mW cm^{-2})	$Et_3N + O_2$ (89 μm)	51	
17	mpg-CN-8nm-193	193	–	Et_3N	1.4	
18	–		465 nm (10±2 mW cm^{-2})	Et_3N	4.0	
19	–		–	Et_3N	1.1	
20	mpg-CN-6nm-204	204	465 nm (10±2 mW cm^{-2})	Et_3N	27.3±0.6	–
21	mpg-CN-5nm-14	14	465 nm (10±2 mW cm^{-2})	$^{n}Bu_3N$	–	10.6
22	mpg-CN-5nm-28	28	465 nm (10±2 mW cm^{-2})	$^{n}Bu_3N$	–	12.7
23	mpg-CN-5nm-61	61	465 nm (10±2 mW cm^{-2})	$^{n}Bu_3N$	–	11.7
24	mpg-CN-5nm-28	28	465 nm (10±2 mW cm^{-2})	$^{n}Bu_3N$	–	13.7
25	mpg-CN-7nm-84	84	465 nm (10±2 mW cm^{-2})	$^{n}Bu_3N$	–	16.0
26	mpg-CN-7nm-70	70	465 nm (10±2 mW cm^{-2})	$^{n}Bu_3N$	–	14.1
27	mpg-CN-5nm-141	141	465 nm (10±2 mW cm^{-2})	$^{n}Bu_3N$	–	15.8
28	mpg-CN-7nm-149	149	465 nm (10±2 mW cm^{-2})	$^{n}Bu_3N$	–	13.7

29	mpg-CN-17nm-171	171	465 nm (10±2 mW cm^{-2})	$^nBu_3N$	–	15.0
30	mpg-CN-8nm-193	193	465 nm (10±2 mW cm^{-2})	$^nBu_3N$	–	17.2
31	mpg-CN-6nm-204	204	465 nm (10±2 mW cm^{-2})	$^nBu_3N$	–	16.5

[a] Yield of Et_2NH (in case of using Et_3N) and $^nPrC(O)H$ (in case of using $^nBu_3N$) was determined by ^1H NMR with 1,3,5-trimethoxybenzene as internal standard. Average±Std.dev (n = 3).

[b] Et_3N (0.1 mmol).

[c] Et_3N (0.05 mmol).

Supplementary Table 15. Attempt to enable *aza*-pinacol coupling of imine **1a** by mpg-CN-6nm-204(e⁻/H⁺) in dark.

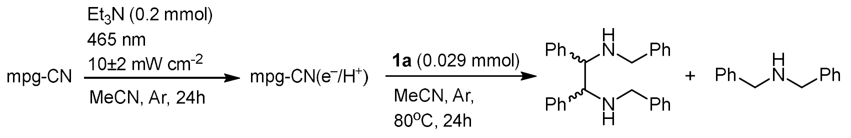

Entry	Semiconductor mass, mg	t,°C	Conversion of **1a**,[a] %	Combined yield of *R,S*-**2a** and *R,R*-**2a**,[a] %	Yield of dibenzylamine,[a] mmol
1	20	25	0	0	0
2	20	50	0	0	0
3	20	80	0	0	0
4	40	80	14	0	$5\cdot10^{-4}$
5	80	80	37	0	$4\cdot10^{-4}$
6	120	80	52	0	$0.5\cdot10^{-4}$

[a] Determined from ^{1}H NMR spectra using 1,3,5-trimethoxybenzene as internal standard.

Supplementary Table 16. Summary of *N* values.

Entry	Semiconductor	S_{SA}, m^2 g^{-1}	SED	δ, mol g^{-1}	*N* (*ab* basal plane)[a]	*N* (*ac* edge plane)[b]	*N* (*bc* edge plane)[c]
1	mpg-CN	204	Et_3N	252	2.6	5.0	6.8
2	mpg-CN	193	Et_3N	250	2.5	4.8	6.5
3	mpg-CN	171	Et_3N	241	2.3	4.4	5.9
4	mpg-CN	149	Et_3N	258	1.9	3.6	4.8
5	mpg-CN	84	Et_3N	269	1.0	1.9	2.6
6	mpg-CN	193	Et_3N + NH_4PF_6	68	9.1	17.6	23.7
7	mpg-CN	204	$^nBu_3N$	187	3.5	6.8	9.1
8	mpg-CN	193	$^nBu_3N$	181	3.4	6.6	8.9
9	mpg-CN	171	$^nBu_3N$	164	3.4	6.5	8.7
10	mpg-CN	149	$^nBu_3N$	187	2.6	5.0	6.7
11	mpg-CN	84	$^nBu_3N$	167	1.6	3.1	4.2
12	mpg-CN	193	$^nBu_3N$ + NH_4PF_6	47	13.1	25.6	34.4
13	mpg-CN	204	$PhCH_2NH_2$	56	11.6	22.5	30.3
14	mpg-CN	193	$PhCH_2NH_2$	48	12.9	25.1	33.8
15	mpg-CN	171	$PhCH_2NH_2$	43	12.9	24.9	33.5
16	mpg-CN	149	$PhCH_2NH_2$	91	5.3	10.2	13.7
17	mpg-CN	84	$PhCH_2NH_2$	53	5.0	9.8	13.2

[a] For mpg-CN, unit cell parameter of melon-type g-CN were used: a = 1.67 nm, b = 1.24 nm, c = 0.32 nm.[18] Areas of the unit cell individual facets are: S_{ab} = 2.071 nm^2 (k = 4), S_{ac} = 0.534 nm^2 (k = 2), S_{bc} = 0.397 nm^2 (k = 2).

Supplementary Table 17. Summary of N_{im} values.

Entry	Semiconductor	SED	Facet	*k*	N_{im}[a]
1	mpg-CN	Et_3N	*ab*	4	0.6
2	mpg-CN	Et_3N	*ac*	2	1.1
3	mpg-CN	Et_3N	*bc*	2	1.5
4	mpg-CN	$^nBu_3N$	*ab*	4	0.9
5	mpg-CN	$^nBu_3N$	*ac*	2	1.7
6	mpg-CN	$^nBu_3N$	*bc*	2	2.2
7	Na-PHI	Et_3N	*ab*	2	0.4
8	Na-PHI	Et_3N	*ac*	1	0.7
9	Na-PHI	Et_3N	*bc*	1	0.7
10	Na-PHI	$^nBu_3N$	*ab*	2	0.5
11	Na-PHI	$^nBu_3N$	*ac*	1	1.1
12	Na-PHI	$^nBu_3N$	*bc*	1	1.1

[a] For mpg-CN, unit cell parameter of melon-type g-CN were used: a = 1.67 nm, b = 1.24 nm, c = 0.32 nm.[18] Areas of the unit cell individual facets are: S_{ab} = 2.071 nm^2, S_{ac} = 0.534 nm^2, S_{bc} = 0.397 nm^2. For H-PHI, unit cell parameter were used: a = 1.28 nm, b = 1.28 nm, c = 0.317 nm.[19] Areas of the unit cell individual facets are: S_{ab} = 1.638 nm^2; S_{ac} = 0.406 nm^2; S_{bc} = 0.406 nm^2. Van der Waals volume of the iminium cation derived from Et_3N is 0.12113 nm^3, $^nBu_3N$ 0.2232 nm^3, was obtained from ChemAxon Marvin Suite.

Supplementary Table 18. Stability of *R,S*-**2a** and *R,R*-**2a** under the conditions of photocatalysis.

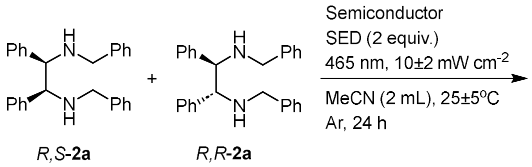

Entry	Semiconductor (mass)	SED	d.r. between *R,S*-**2a** and *R,R*-**2a** before the reaction[a]	*R,S*-**2a** and *R,R*-**2a** recovered after the reaction,[a] %	d.r. between *R,S*-**2a** and *R,R*-**2a** after the reaction[a]
1	mpg-CN-8nm-193 (20 mg)	$^{i}Pr_2NEt$ (0.2 mmol, 35 µL)	0.02:1	74	0.02:1
2	mpg-CN-8nm-193 (20 mg)	Et_3N (0.2 mmol, 28 µL)	0.02:1	100	0.02:1
3	mpg-CN-8nm-193 (20 mg)	$^{n}Bu_3N$ (0.2 mmol, 48 µL)	0.02:1	81	0.02:1
4	CdS (20 mg)	$^{i}Pr_2NEt$ (0.2 mmol, 35 µL)	0.02:1	99	0.02:1
5	CdS (20 mg)	Et_3N (0.2 mmol, 28 µL)	0.02:1	99	0.02:1
6	CdS (20 mg)	$^{n}Bu_3N$ (0.2 mmol, 48 µL)	0.02:1	99	0.02:1
7	mpg-CN-8nm-193 (20 mg)	$^{i}Pr_2NEt$ (0.2 mmol, 35 µL)	1:0	97	1:0
8	mpg-CN-8nm-193 (20 mg)	Et_3N (0.2 mmol, 28 µL)	1:0	97	1:0
9	mpg-CN-8nm-193 (20 mg)	$^{n}Bu_3N$ (0.2 mmol, 48 µL)	1:0	96	1:0

[a] Determined from ^{1}H NMR spectra using 1,3,5-trimethoxybenzene as internal standard.

Supplementary Table 19. Adsorption energies in solvated Na-PHI.

Structure	Adsorption energy, kJ mol^{-1}
H+/2-Na-PHI	-32.29
H+/4-Na-PHI	-69.30
H+/8-Na-PHI	-65.54
H+/20-Na-PHI	0.62
NH4/2-Na-PHI	117.42
NH4/4-Na-PHI	63.78
NH4/8-Na-PHI	-24.40
NH4/20-Na-PHI	117.56

Supplementary Table 20. Screening of reaction conditions of *aza*-pinacol coupling of imine **1a** using benzylamine as electron donor.

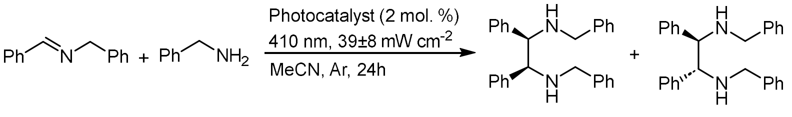

Entry	Photocatalyst	Imine yield,[a] mmol	Benzylamine,[a] mmol	Yield of *R,S*-**2a**,[a] mmol	Yield of *R,R*-**2a**,[a] mmol	Mass balance,[b] %
1	$Ir(ppy)_2(dtbbpy)PF_6$	0.064	0.064	0.025	0.021	100
2	$Ir(ppy)_3$	0.070	0.078	0.0001	0.0001	100

[a] Determined from ^{1}H NMR spectra using 1,3,5-trimethoxybenzene as internal standard.

[b] Calculated according to the equation:

$$x = \frac{4\times n(R,S-\mathbf{2a})+4\times n(R,R-\mathbf{2a})+2\times n(imine)+n(BA)}{2\times n(imine)+n(BA)} \quad (10)$$

where *n*(*R,S*-**2a**) – amount of *R,S*-**2a** formed, mol. *n*(*R,R*-**2a**) – amount of *R,R*-**2a** formed, mol. *n*(imine) – amount of imine **1a** detected in the reaction mixture after the photocatalytic experiment was completed, mol. *n*(BA) – amount of benzylamine detected in the reaction mixture after the photocatalytic experiment was completed, mol.

Supplementary Table 21. Summary of semiconductors textural properties.

Entry	Semiconductor	Surface area, $m^2\ g^{-1}$	Average pore diameter, nm	Cumulative pore volume, $cm^3\ g^{-1}$
1	mpg-CN-6nm-204	204	6	0.41
2	mpg-CN-8nm-193	193	8	0.51
3	mpg-CN-17nm-171	171	17	0.68
4	mpg-CN-7nm-149	149	7	0.30
5	mpg-CN-5nm-141	141	5	0.22
6	mpg-CN-7nm-84	84	7	0.18
7	mpg-CN-7nm-70	70	7	0.16
8	mpg-CN-5nm-61	61	5	0.085
9	mpg-CN-5nm-33	33	5	0.068
10	mpg-CN-5nm-28	28	5	0.053
11	mpg-CN-5nm-14	14	5	0.033
12	CdS	57	Poorly defined	0.17
13	TiO_2 (anatase, < 25 nm)	106	7	0.19
14	WO_3	5	Poorly defined	0.024
15	Na-PHI	1	5.2	0.007
16	K-PHI	89[1]	–	–

Supplementary Table 22. Structure data for Na-PHI in .xyz format.

Cell parameters: a=12.5477 Å, b=12.5022 Å, c=4.1936 Å, α=92.4711°, β=129.6662°, γ=120.3051°

32

#

N	8.0139034463	-0.0953680907	0.0000000000
N	6.0483849769	1.2136376888	0.0000000000
N	4.1734372030	2.5178482712	0.0000000000
N	8.1919080433	2.1799503062	0.0000000000
N	2.3044183297	3.8083396815	0.0000000000
N	6.2818006859	3.5221222865	0.0000000000
N	0.2223402522	4.7582370208	0.0000000000
N	4.4093774235	4.8821788184	0.0000000000
N	8.3833021860	4.4665322597	0.0000000000
N	-1.8659152989	5.7299465787	0.0000000000
N	2.1162393407	6.1546551090	0.0000000000
N	6.4807839093	5.8737608112	0.0000000000
N	-3.9926439777	6.8200164002	0.0000000000
N	0.0029106771	7.0827544612	0.0000000000
N	-2.1215408543	8.0825831548	0.0000000000
N	1.9163775747	8.4266685407	0.0000000000
N	-0.2391820364	9.3794284004	0.0000000000
C	7.3711971218	1.1106857415	0.0000000000
C	5.4699029790	2.4193231259	0.0000000000
C	3.6158710633	3.7891662538	0.0000000000
C	7.6408428343	3.3913859050	0.0000000000
C	1.5554114291	4.9458745220	0.0000000000
C	5.7052140785	4.7927637463	0.0000000000
C	-0.5666694472	5.8382407944	0.0000000000
C	7.8181125350	5.6989711923	0.0000000000
C	-2.6578198282	6.8481832948	0.0000000000
C	1.3585742574	7.2480044792	0.0000000000
C	-0.8022515851	8.2088579928	0.0000000000
C	1.0932716814	9.5385510001	0.0000000000
Na	4.2210306815	7.2777432003	0.0218049699
Na	4.1580858463	-0.2484744748	-0.3839625030
Na	-1.9220402417	3.2822012369	-0.1902122011

Supplementary Table 23. Structure data for H-PHI in .xyz format.
Cell parameters: a=13.0403 Å, b=13.0407 Å, c=4.2076 Å, α=112.5021°, β=112.3852°, γ=119.8127°

32
#

N	7.7412508209	0.0238946954	0.0000000000
N	5.9413928794	1.5074257335	0.0000000000
N	4.2972235781	3.1046954220	0.0000000000
N	8.2308386507	2.1923408174	0.0000000000
N	2.6651078230	4.5990605099	0.0000000000
N	6.5298372993	3.7769393354	0.0000000000
N	0.5572378170	5.2722087899	0.0000000000
N	4.8624584234	5.4152921563	0.0000000000
N	8.8230991066	4.4049235952	0.0000000000
N	-1.6460231252	5.9045641311	-0.0000000000
N	2.2809892442	6.9115106316	0.0000000000
N	7.0960850491	6.0317803377	0.0000000000
N	-3.8280210119	6.7285481676	-0.0000000000
N	0.0304570658	7.5435924506	-0.0000000000
N	-2.1900291368	8.2315238173	-0.0000000000
N	1.7053630145	9.1561110883	0.0000000000
N	-0.5647809578	9.8456142030	-0.0000000000
C	7.2427923797	1.2885331372	0.0000000000
C	5.5693015135	2.7737729651	0.0000000000
C	4.0126356493	4.4069785539	0.0000000000
C	7.8994018388	3.4522943147	0.0000000000
C	1.8287478717	5.6727152654	0.0000000000
C	6.1452733217	5.1100104912	0.0000000000
C	-0.3622383112	6.2113907858	-0.0000000000
C	8.3676777160	5.6457010965	0.0000000000
C	-2.4828546465	6.9248776538	-0.0000000000
C	1.3783831947	7.8727499794	0.0000000000
C	-0.9321035178	8.5705084101	-0.0000000000
C	0.7381942895	10.0673247473	-0.0000000000
H	-4.3328075907	7.5935992326	0.0967237058
H	2.1632236078	3.7332674651	0.0659469812
H	8.7427267504	0.0161098629	0.0967753972

Supplementary Table 24. Variety of charges while hydrogen is adsorbed on Na-PHI, calculated via DDEC6.

	Nitrogen, [e]	Carbon, [e]	Sodium, [e]	bridging-N/ interacting-N, [e]	H, [e]
2-Na-PHI	-0.54 ± 0.13	0.52 ± 0.03	0.88 ± 0.01	-0.39	0.31
4-Na-PHI	-0.54 ± 0.12	0.53 ± 0.03	0.88 ± 0.01	-0.39	0.32
8-Na-PHI	-0.54 ± 0.12	0.53 ± 0.03	0.89 ± 0.01	-0.52	0.32
20-Na-PHI	-0.54 ± 0.12	0.54 ± 0.03	0.89 ± 0.01	-0.53	0.32

Supplementary Table 25. Variety of charges while ammonium is adsorbed on Na-PHI, calculated via DDEC6.

	Nitrogen, [e]	Carbon, [e]	Sodium, [e]	bridging-N/ interacting-N, [e]	N (Ammonium), [e]	~H (Ammonium), [e]
2-Na-PHI	-0.54 ± 0.13	0.52 ± 0.02	0.87 ± 0.02	-0.38	-0.90	0.30 ± 0.01
4-Na-PHI	-0.54 ± 0.12	0.53 ± 0.03	0.88 ± 0.01	-0.38	-0.90	0.30 ± 0.01
8-Na-PHI	-0.54 ± 0.12	0.53 ± 0.03	0.89 ± 0.01	-0.53	-0.86	0.30 ± 0.01
20-Na-PHI	-0.54 ± 0.12	0.54 ± 0.03	0.89 ± 0.01	-0.53	-0.88	0.30 ± 0.01

Supplementary Table 26. Charges of atoms in Na-PHI.

	Arithmetic mean Charge,[a] [e]	Standard Deviation, [e]
N1	-0.215	0.001
N2	-0.592	0.017
N3	-0.584	0.033
N4	-0.547	0.017
C1	0.562	0.023
C2	0.512	0.008
Na	0.885	0.01

[a] Charges were calculated for 20-PHI systems via DDEC6. Atom Labels are depicted in Supplementary Figure 17.

Supplementary Table 27. Charges of atoms in H-PHI.

	Arithmetic mean Charge,[a] [e]	Standard Deviation, [e]
N1	-0.257	0.005
N2	-0.507	0.020
N3	-0.479	0.017
N4	-0.370	0.003
C1	0.552	0.017
C2	0.529	0.018

[a] Charges were calculated for 20-PHI systems via DDEC6. Atom Labels are depicted in Supplementary Figure 17.

Supplementary Table 28. Charges of atoms in Na-PHI solvated with 5-H_2O per Na atom.

	Arithmetic mean Charge,[a] [e]	Standard Deviation, [e]
N1	-0.231	0.014
N2	-0.526	0.044
N3	-0.519	0.023
N4	-0.485	0.056
C1	0.568	0.036
C2	0.539	0.016
Na	0.911	0.012

[a] Charges were calculated for 20-PHI systems via DDEC6. Atom Labels are depicted in Supplementary Figure 17.

Supplementary Table 29. Distance Na-Backbone in Na-PHI.

	Average distance [Å]	Standard deviation
Na-N2 distance	2.64	0.096
Na-N2 distance (with water)	2.85	0.552
Na-N3 distance	2.41	0.080
Na-N3 distance (with water)	3.12	0.556

Supplementary Table 30. Results of kinetics study.

Time, min	Conv. **1a**, %	Conv. Et_3N, %	Yield *R*,*S*-**2a**, %	Yield *R*,*R*-**2a**, %	Yield *R*,*S*-**2a** and *R*,*R*-**2a**, %	Yield Et_2NH, %	δ, μmol g^{-1}
0	0	66	0	0	0	0	12
48±3	5	70	5	4	9	2	
75±3	14	70	7	5	12	3	
164±3							17
174±3	24	76	12	10	22	5	
193±3	31	76	13	9	22	6	
202±3							22
215±3	32	77	16	11	27	6	
899±3							80
905±3	72	86	19	23	42	14	
918±3							78
995±3	74	87	19	24	42	14	
1168±3							95
1176±3	79	89	19	24	43	15	
1465±3	86	91	20	28	48	16	
1807±3							121
1826±3	92	92	19	27	46	17	
2541±3							116
2559±3	98	95	20	28	48	18	

Supplementary Table 31. Correlation of benzylamine conversion with the surface area of the semiconductor.

Semiconductor	t, °C	*m*, mg	S_{SA}, m^2 g^{-1}	Log(*S*), m^2[a]	Conversion, %[b]
mpg-CN	25±5	20	193	0.58659	59
mpg-CN	80	20	193	0.58659	62
mpg-CN	80	40	193	0.88762	76
mpg-CN	80	80	193	1.18865	85
mpg-CN	80	160	193	1.48968	99
Na-PHI	25±5	5.8	1	-2.27572	18
Na-PHI	25±5	10.2	1	-2	19
Na-PHI	25±5	21.3	1	-1.67366	21
Na-PHI	25±5	40	1	-1.39794	38
Na-PHI	25±5	80	1	-1.09691	43
Na-PHI	25±5	120	1	-0.92082	47
Na-PHI	25±5	160	1	-0.79588	54
Na-PHI	25±5	5	35	-0.75696	37
Na-PHI	25±5	10	35	-0.45593	52
Na-PHI	25±5	20	35	-0.1549	76
Na-PHI	80	5.3	1	-2.27572	13
Na-PHI	80	10	1	-2	15
Na-PHI	80	21.2	1	-1.67366	30
Na-PHI	80	40	1	-1.39794	36
Na-PHI	80	80	1	-1.09691	43
Na-PHI	80	120	1	-0.92082	51
Na-PHI	80	160	1	-0.79588	61
Na-PHI	80	5	35	-0.75696	54
Na-PHI	80	10	35	-0.45593	52
Na-PHI	80	20	35	-0.1549	71
Na-PHI	80	40	35	0.14613	75
Na-PHI	80	80	35	0.44716	90
Na-PHI	80	120	35	0.62325	92

[a] Surface area of the semiconductor in a specific experiment was determined according to the equation:

$$S = S_{SA} \cdot m \cdot 10^{-3} \quad (11)$$

where S_{SA} – specific surface area of the semiconductor, m^2 g^{-1}. *m* – mass of the semiconductor used in a specific experiment, mg.

[b] Data from Supplementary Table 1.

Supplementary Table 32. Fitting parameters.

**Semiconductor**	**t, °C**	**k**	**b**	**R^2**
mpg-CN	80	39.51458	39.469	0.989
Na-PHI	25±5	25.19508	69.53221	0.835
Na-PHI	80	27.6296	74.42998	0.967

Data shown in Supplementary Table 31 were fitted with function:

$$\text{Conversion} = k \cdot \log(S) + b \tag{12}$$

Supplementary Table 33. Correlation of the number of electrons stored in g-CN materials with specific surface area.

Material	$S_{S.A.}$, m^2 g^{-1}	$N_{(ac)}$, µmol g^{-1}[a]	$N_{(ab)}$, µmol g^{-1}[a]	$N_{(bc)}$, µmol g^{-1}[a]	N_{total}, µmol g^{-1}[b]	$N_{(ac)}$/δ(Et_3N)[c]	$N_{(ab)}$/δ(Et_3N)[c]	$N_{(bc)}$/δ(Et_3N)[c]	N_{total}/δ(Et_3N)[c]	$N_{(ac)}$/δ(Bu_3N)[c]	$N_{(ab)}$/δ(Bu_3N)[c]	$N_{(bc)}$/δ(Bu_3N)[c]	N_{total}/δ(Bu_3N)[c]
mpg-CN	204	1268	655	1708	4975	5.0	2.6	6.8	20	6.8	3.5	9.1	27
mpg-CN	193	1200	619	1616	4975	4.8	2.5	6.5	20	6.6	3.4	8.9	27
mpg-CN	171	1063	549	1432	4975	4.4	2.3	5.9	21	6.5	3.4	8.7	30
mpg-CN	149	926	478	1248	4975	3.6	1.9	4.8	19	5.0	2.6	6.7	27
mpg-CN	84	522	270	703	4975	1.9	1.0	2.6	19	3.1	1.6	4.2	30
Na-PHI	1	8	2	2	4831	0.023	0.006	0.023	14	0.033	0.009	0.033	20
Na-PHI	35	285	76	285	4831	0.8	0.2	0.8	14	1.2	0.3	1.2	20

[a] $N_{(ac)}$, $N_{(ab)}$, $N_{(bc)}$ define specific number of heptazine units on the surface of g-CN material (in µmol per gram) assuming that the materials surface is terminated by (ac), (ab) and (bc) crystal cell facets, respectively. $N_{(ac)}$, $N_{(ab)}$, $N_{(bc)}$ were calculated according to the equation:

$$N_{(xx)} = \frac{k \cdot S_{S.A.} \cdot 10^{18} \cdot 10^{6}}{S_{cf} \cdot N_A} \quad (13)$$

where S_{cf} – area of the single unit cell facet, nm^2. For mpg-CN, unit cell parameter of melon-type g-CN were used: a = 1.67 nm, b = 1.24 nm, c = 0.32 nm.[18] Areas of the unit cell individual facets are: S_{ab} = 2.071 nm^2, S_{ac} = 0.534 nm^2, S_{bc} = 0.397 nm^2. For Na-PHI, unit cell parameters are: a = b = 1.238 nm, c = 0.33 nm.[2] Areas of the unit cell individual facets are: S_{ab} = 1.533 nm^2, S_{ac} = S_{bc} = 0.409 nm^2. k – number of heptazine units per unit cell projected onto specific cell facet. For mpg-CN facets (ac) and (bc), k = 2, facet (ab) k = 4. For Na-PHI facets (ac), (bc) and (ab), k = 2. See Supplementary Fig. 2 for visualization of g-CN materials crystal cells. N_A – Avogadro's number, 6.02·10^{23} mol^{-1}.

[b] Total specific number of heptazine units in g-CN material (in µmol g^{-1}) was calculated according to the equation:

$$N_{total} = \frac{k_c \cdot 10^{6}}{M_w} \quad (14)$$

where k_c – number of heptazine units in the unit cell. For mpg-CN, k_c = 4. For Na-PHI, k_c = 2 (see Supplementary Fig. 2 for visualization of the materials unit cells). M_w – molar mass of the unit cell, g mol^{-1}. For mpg-CN, M_w = 804 g mol^{-1}, Na-PHI 414 g mol^{-1}.

[c] The values were calculated by dividing $N_{(xxx)}$ values by δ values obtained by the materials photocharging (20 mg) in the presence of Et_3N (0.2 mmol) or $^nBu_3N$ (0.2 mmol) in the absence of NH_4PF_6 (Supplementary Table 4 and Figure 4b). It is assumed that for Na-PHI with S_{SA} = 35 and 1 m^2 g^{-1}, δ values are the same.

Supplementary Table 34. Correlation of the number of electrons accumulated in Na-PHI with the number of Na^+ extracted from Na-PHI.

m(Na-PHI), mg[a]	t, °C[b]	ω(Na), wt. %[c]	n_{Na+}, µmol[d]	δ, µmol g^{-1}[e]	n_{e-}, µmol[f]
5	25±5	12.2±0.48	3	644	3.2
10	25±5	10.3±2.26	15	470	4.7
20	25±5	10.2±0.61	30	353	7.1
40	25±5	10.2±3.16	61	282	11.2
80	25±5	10.6±1.06	108	–	–
120	25±5	11.2±1.76	130	–	–
160	25±5	11.4±0.3	160	–	–
5	80	11.0±2.79	6	445	2.2
10	80	9.41±1.06	19	202	2.0
20	80	9.66±0.79	35	106	2.1
40	80	10.2±0.61	61	92	3.7
80	80	11.3±1.36	83	–	–
120	80	11.9±0.81	94	–	–
160	80	12.0±1.26	118	–	–

[a] Mass of Na-PHI ($S_{S.A.}$ 1 m^2 g^{-1}) taken to perform benzylamine tetramerization.

[b] Temperature of benzylamine tetramerization reaction.

[c] Na content in Na-PHI after recovery from the reaction mixture. Determined by ICP-OES. Na content in fresh Na-PHI is ω(Na_{ref}) = 13.7±1.67 wt. % (mean±std., n = 3).

[d] The amount of Na^+ (µmol) extracted from Na-PHI was calculated according to the equation:

$$n_{Na^+} = \frac{(\omega(Na_{ref}) - \omega(Na))\cdot 10^{-2}\cdot m(\text{Na–PH })\cdot 10^{-3}\cdot 10^{6}}{A_{Na}} \quad (15)$$

where ω(Na_{ref}) – Na content in fresh Na-PHI, ω(Na_{ref}) = 13.7±1.67 wt. % (mean±std, n = 3). ω(Na) – Na content in Na-PHI recovered from the reaction mixture, wt. %. m(Na-PHI) – mass of Na-PHI taken to perform benzylamine tetramerization, mg. A_{Na} – atomic mass of sodium, A_{Na} = 23 g mol^{-1}.

[e] Specific concentration of electrons (δ) in Na-PHI that was photocharged in the presence of benzylamine at room temperature or at 80°C. Data were taken from Supplementary Table 4.

[f] The amount of electrons stored in Na-PHI (mol) was calculated according to the equation:

$$n_{e^-} = \delta \cdot 10^{-6} \cdot m(\text{Na} - \text{PHI}) \cdot 10^{-3} \quad (16)$$

Supplementary Figures

1. James, Glorius et al.[20]

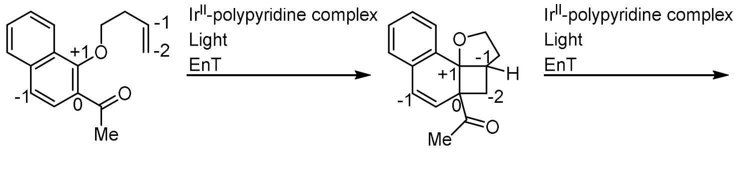

2. Metternich and Gilmour[21]

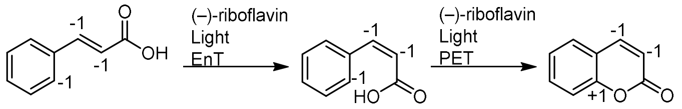

3. Neveselý, Daniliuc and Gilmour[22]

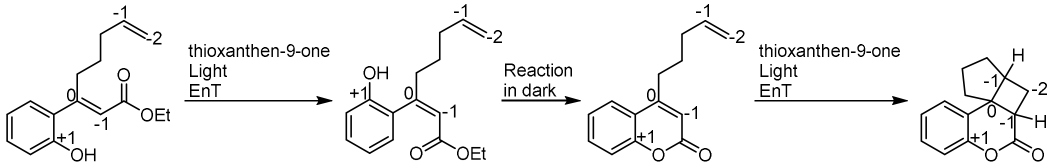

4. Ma, Houk, Glorius et al.[23]

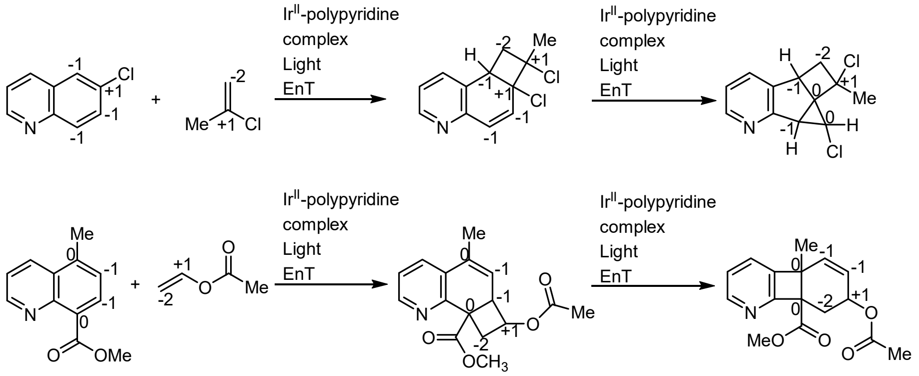

5. Yan, Chen, Xiao et al.[24]

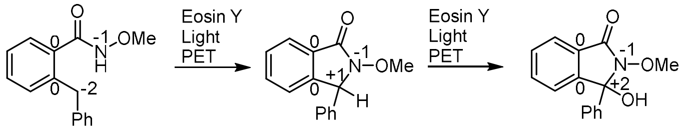

6. Wang, Li, Tang et al.[25]

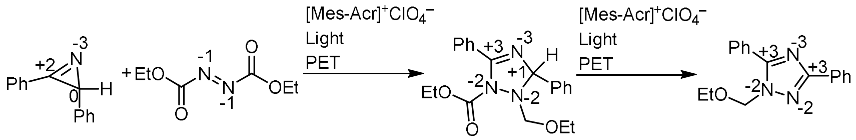

Supplementary Fig. 1. **Overview of photocatalytic cascade processes mediated by molecular sensitizers.**

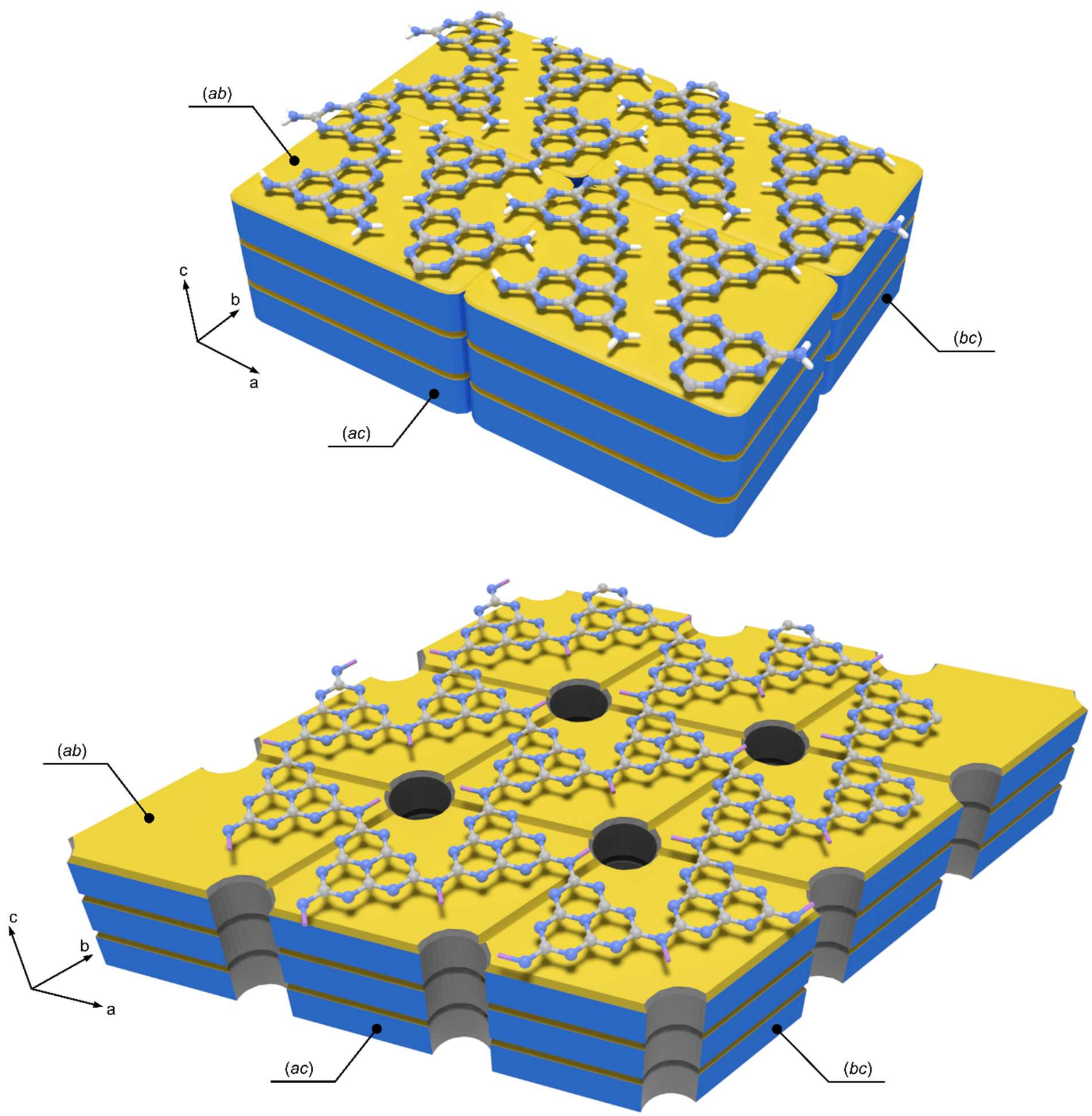

Supplementary Fig. 2. **Visualization of mpg-CN (melon-type g-CN)[18] and PHI unit cells.**[6]
Blue spheres correspond to nitrogen, grey – carbon, white – hydrogen.

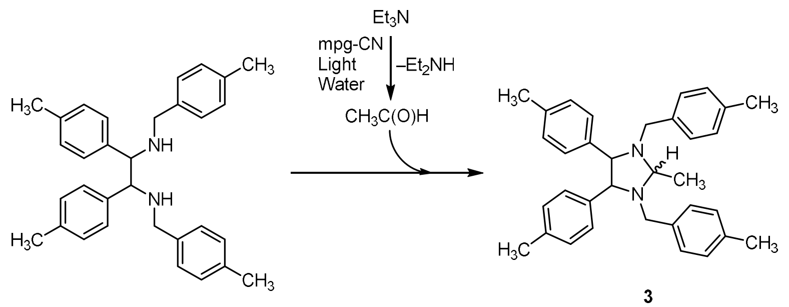

3

$[M+H]^+$ calcd. for $C_{34}H_{39}N_2^+$, 475.3108
found, 475.2946

Supplementary Fig. 3. **Structure of imidazolidine that is formed upon 2c condensation with acetaldehyde.**

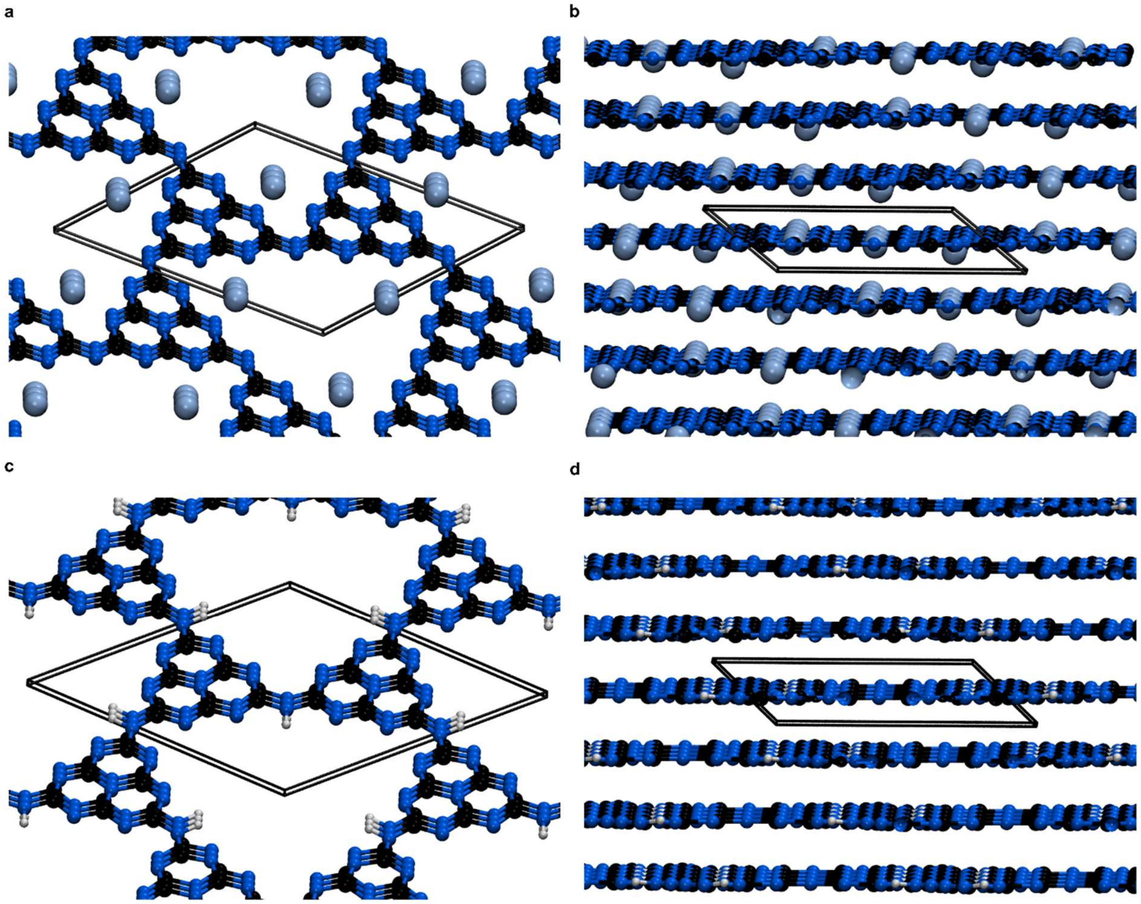

Supplementary Fig. 4. **Geometrically optimized structures of PHIs.**
a Top view on Na-PHI. **b** Side view on Na-PHI. **c** Top view on H-PHI. **d** Side view on H-PHI. Black lines indicate unit cell.

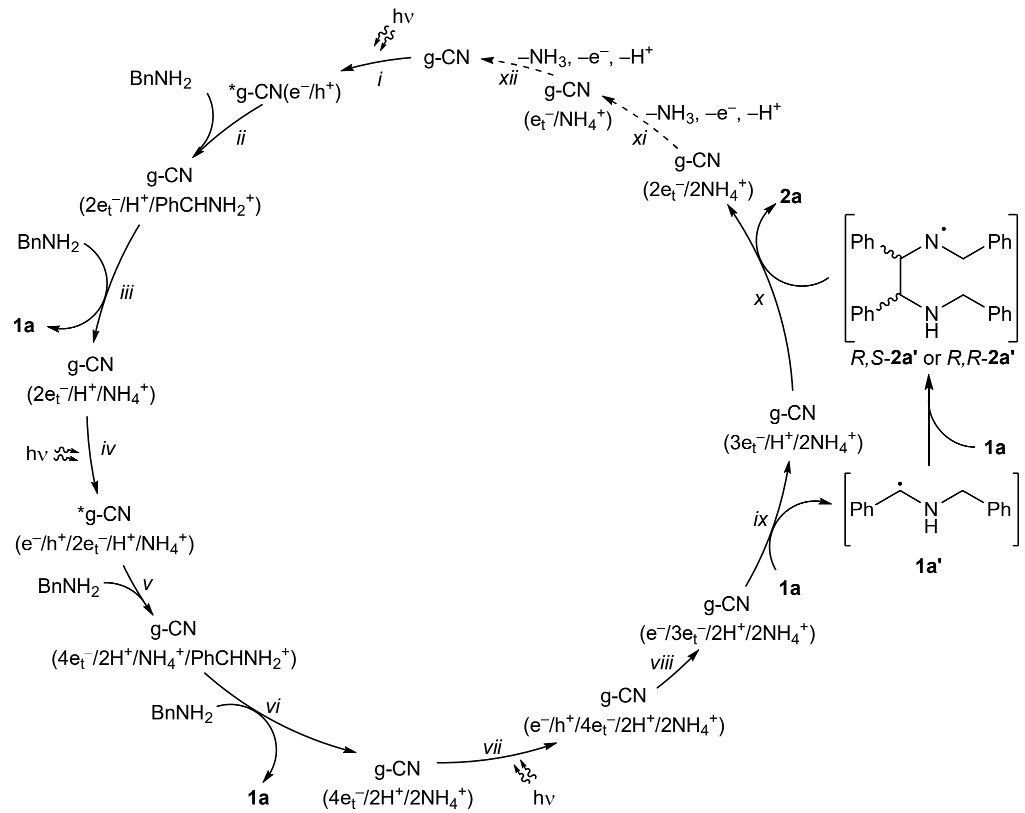

Supplementary Fig. 5. **Proposed mechanism of benzylamine tetramerization mediated by graphitic carbon nitride semiconductor.**

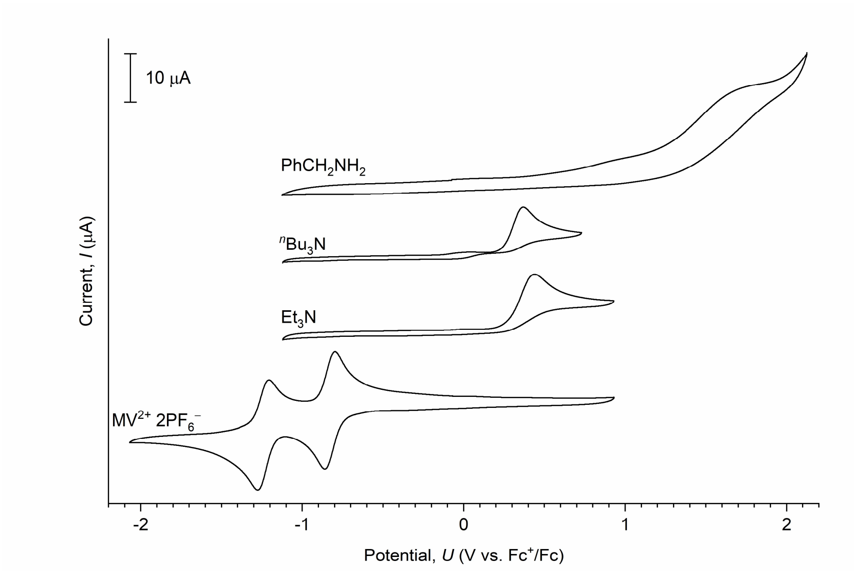

Supplementary Fig. 6. **CV curves of MV^{2+} $2PF_6$, Et_3N, $^{n}Bu_3N$ and $PhCH_2NH_2$ in 0.1 M solution of $^{n}Bu_4N^+$ ClO_4^- in MeCN.**
Scan rate 50 mV s^{-1}.

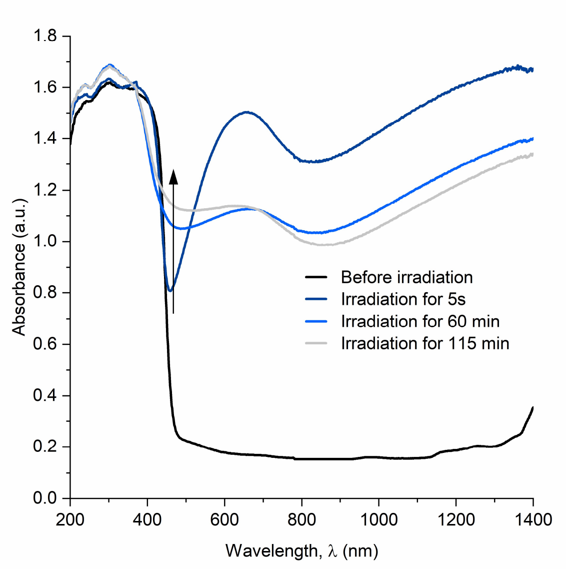

Supplementary Fig. 7. **DRUV-vis spectra of Na-PHI photocharged using NH_4COOH.** Irradiation at 410 nm, 4 W optical power, photon flux 14 µmol s^{-1}.

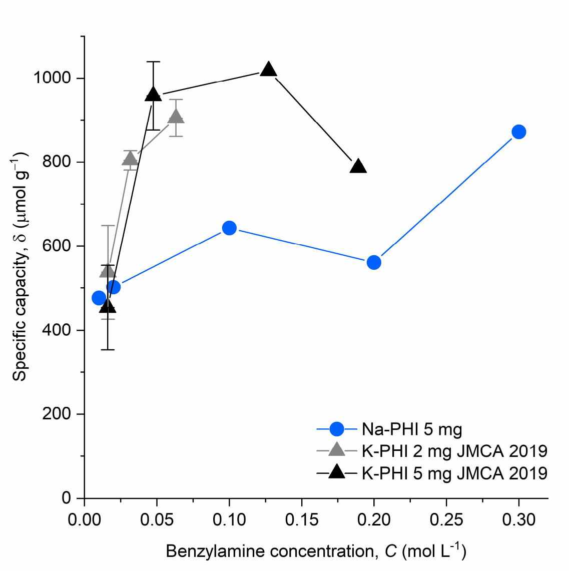

Supplementary Fig. 8. **Dependence of δ on concentration of benzylamine**. Data points for K-PHI are taken from reference [4].

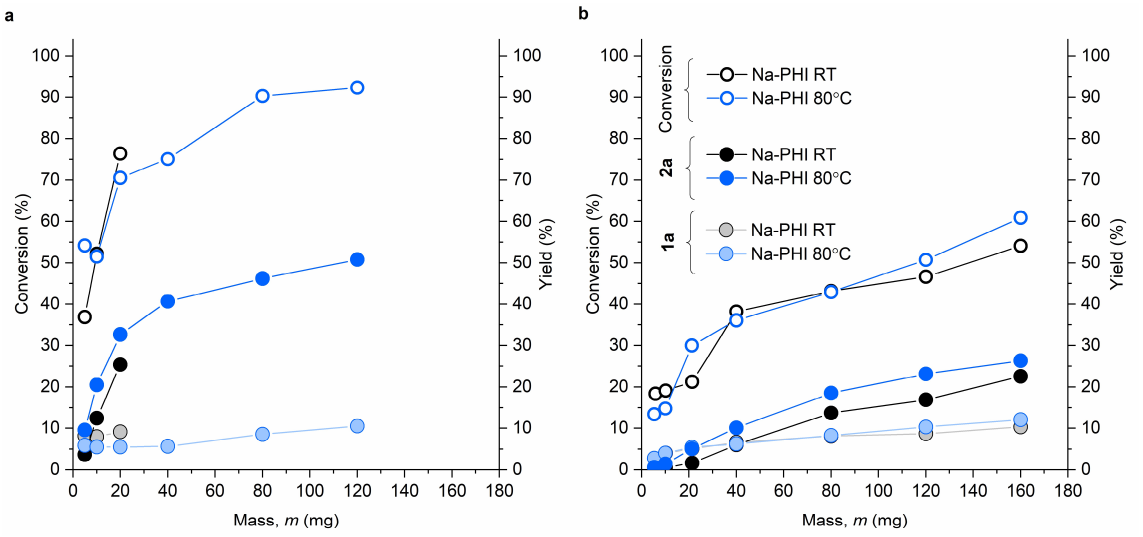

Supplementary Fig. 9. **Benzylamine tetramerization mediated by Na-PHI**.
a Na-PHI with S_{SA} 35 m^2 g^{-1}. **b** Na-PHI with S_{SA} 1 m^2 g^{-1}.

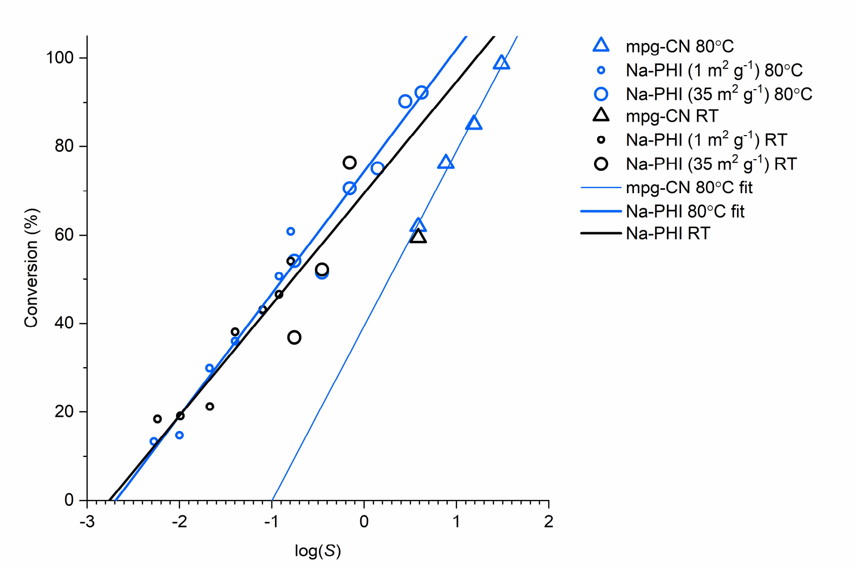

Supplementary Fig. 10. **Dependence of benzylamine conversion on the surface area of the semiconductor.**

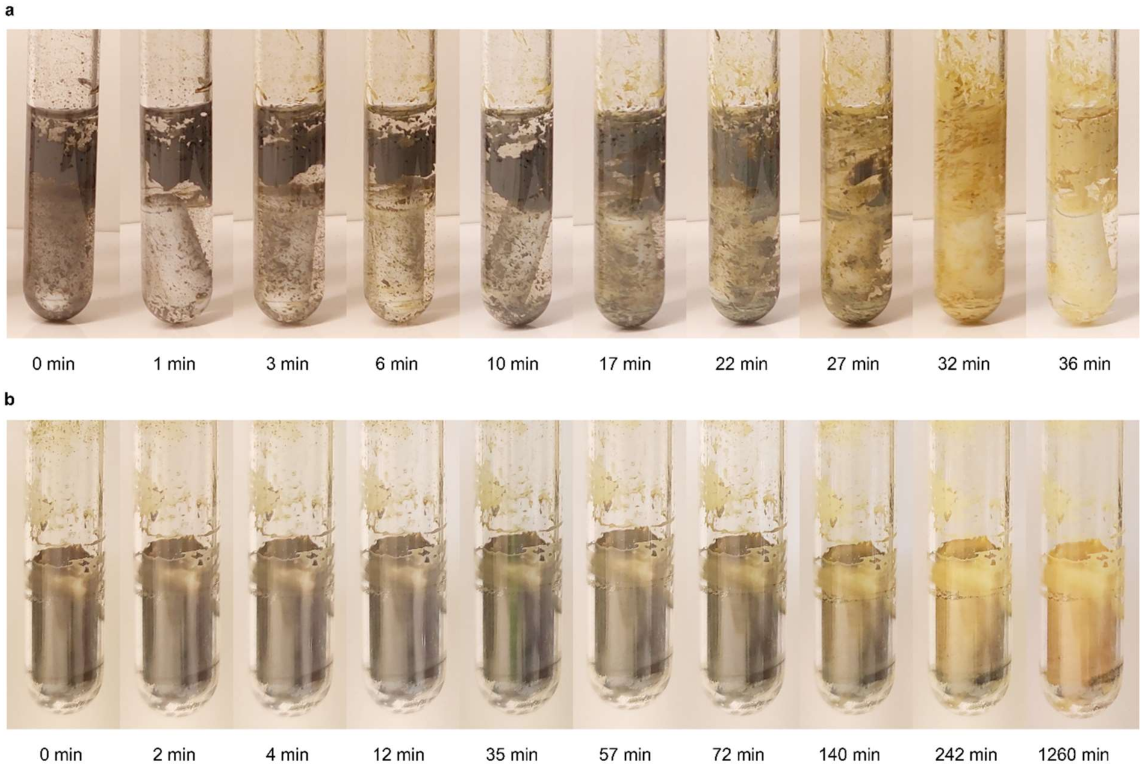

Supplementary Fig. 11. **Oxidation of Na-PHI photocharged using NH_4COOH**.
a Reaction mixture was exposed to air. **b** Solution was decanted followed by drying the residue in vacuum and exposure to air.

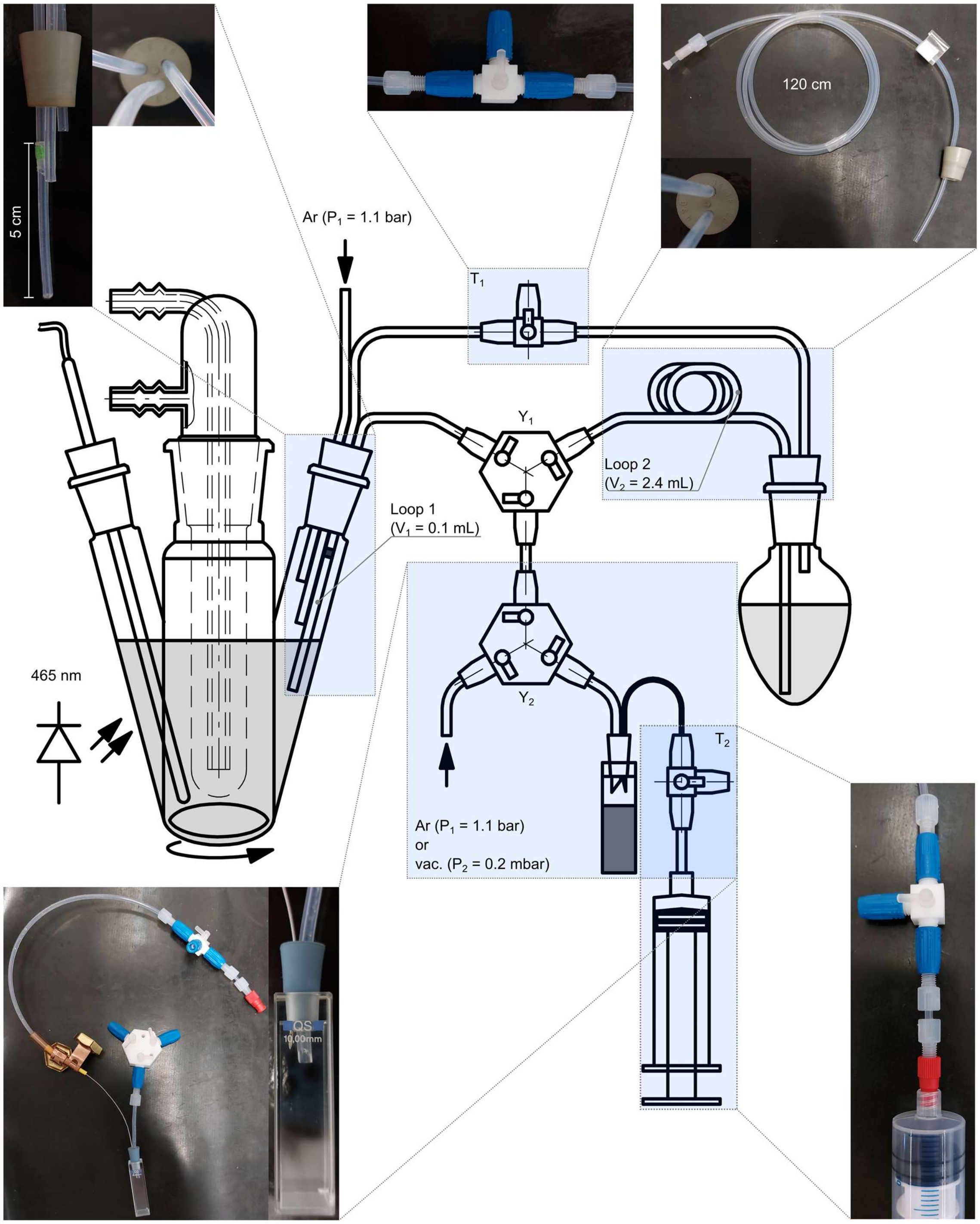

Supplementary Fig. 12. **Schematic representation of the setup and photographs of its elements that was used to perform kinetic measurements studies.**

Absolute pressure values are shown.

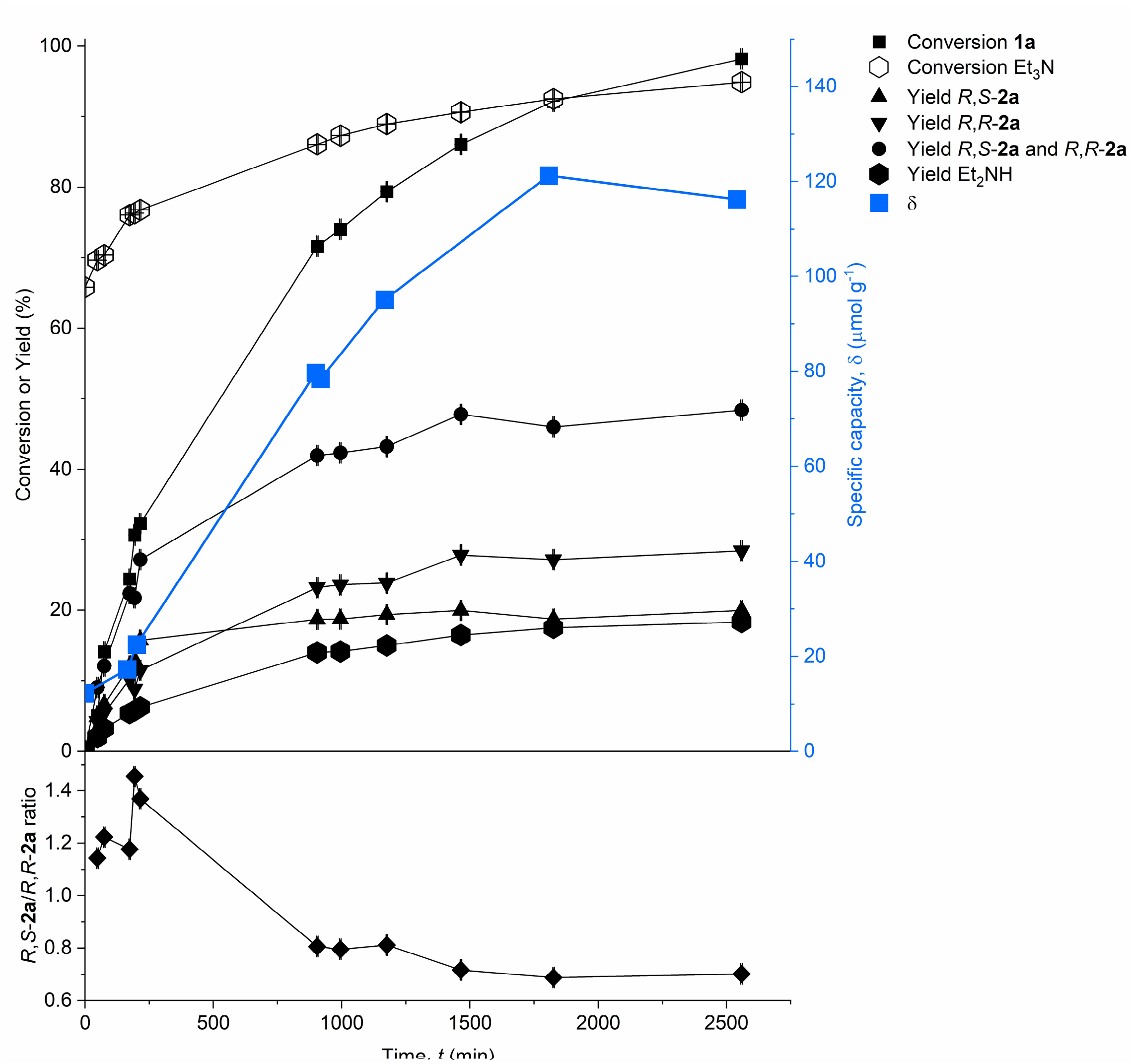

Supplementary Fig. 13. **Results of kinetics study.**

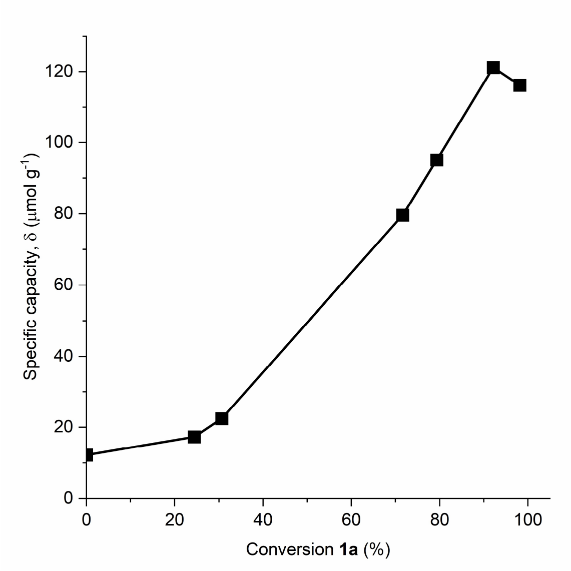

Supplementary Fig. 14. **Dependence of δ on 1a conversion.**

These data points were acquired at 0, 169±5, 197±5, 902±3, 1172±4, 1816±9, ±2550±9 min and taken from Supplementary Table 30.

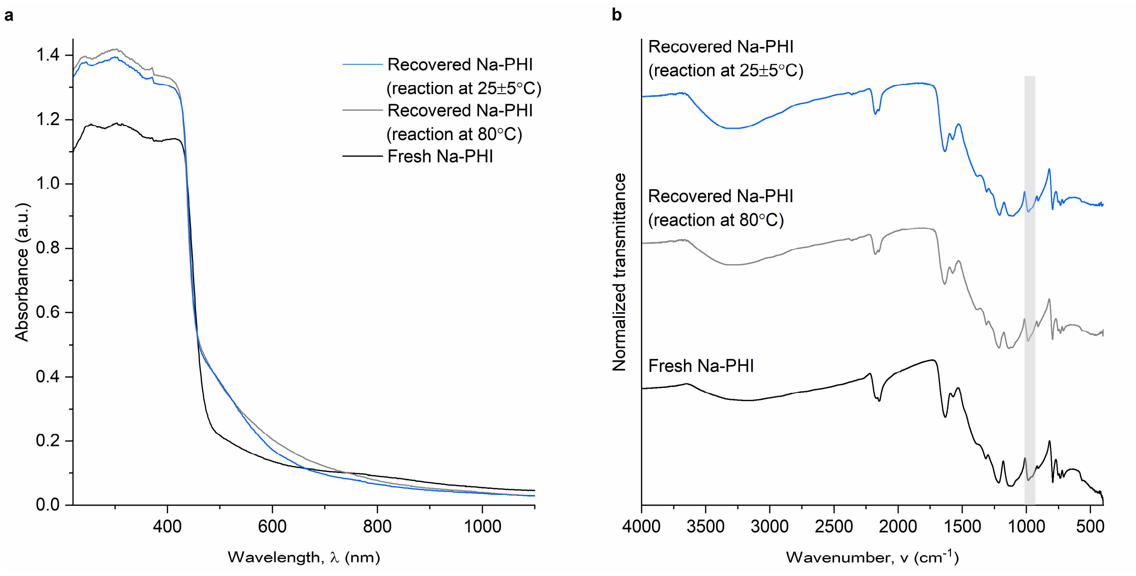

Supplementary Fig. 15. **Characterization of Na-PHI recovered after benzylamine tetramerization.** Reaction was conducted at room temperature and at 80°C. Na-PHI mass taken for the reaction 80 mg. **a** DRUV-vis spectra. **b** FT-IR spectra.

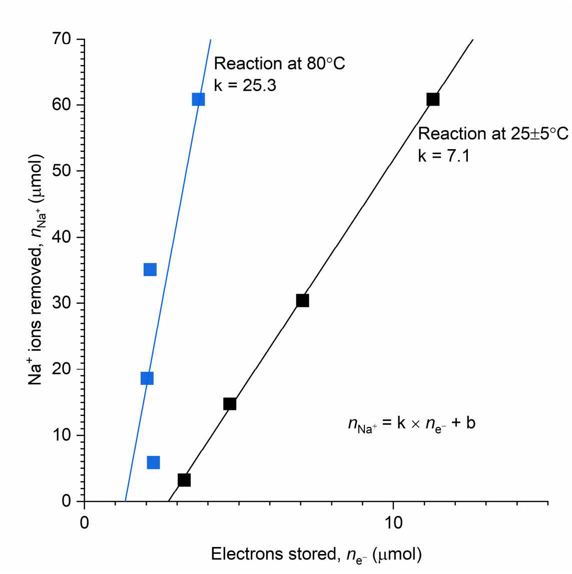

Supplementary Fig. 16. **Correlation of the number of Na^+ extracted from Na-PHI with the number of electrons stored in Na-PHI.**

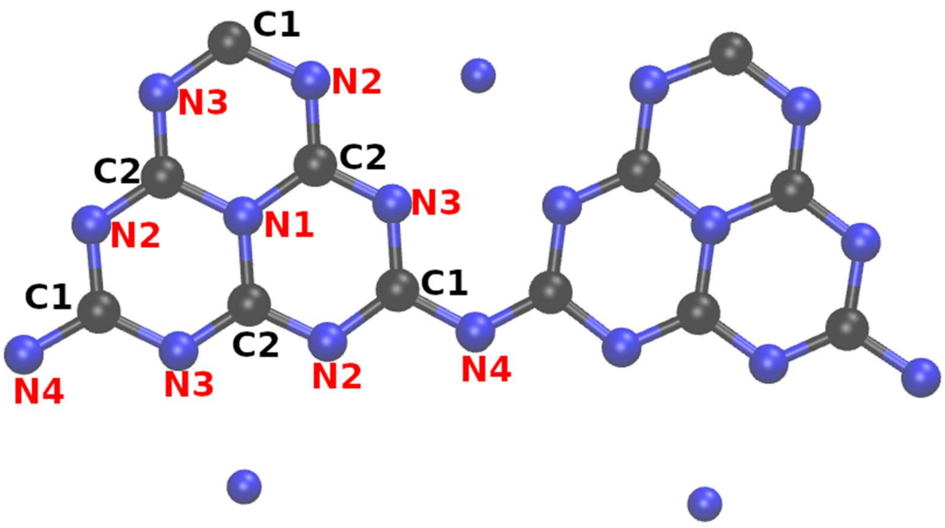

Supplementary Fig. 17. **Charges of the poly(heptazine imide) backbone**.

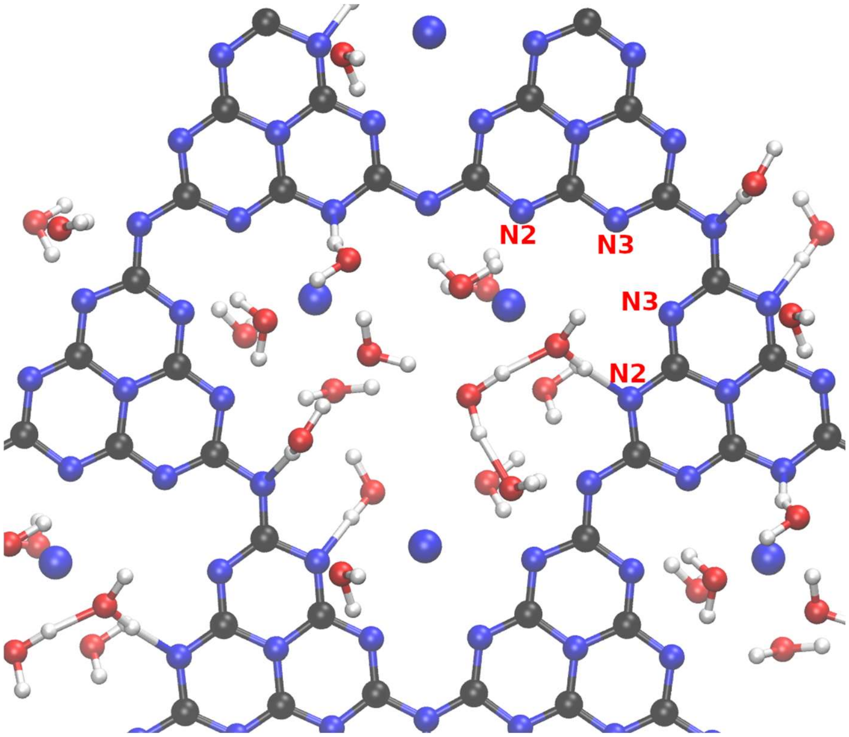

Supplementary Fig. 18. **Structure of Na-PHI with water molecules in the micropore**.
Na-PHI (larger spheres are sodium ions, red oxygen, black carbon, white hydrogen and blue nitrogen) with 5 water molecules per sodium ion. The interlayer distance, compared to non-solvated structure, has been increased.

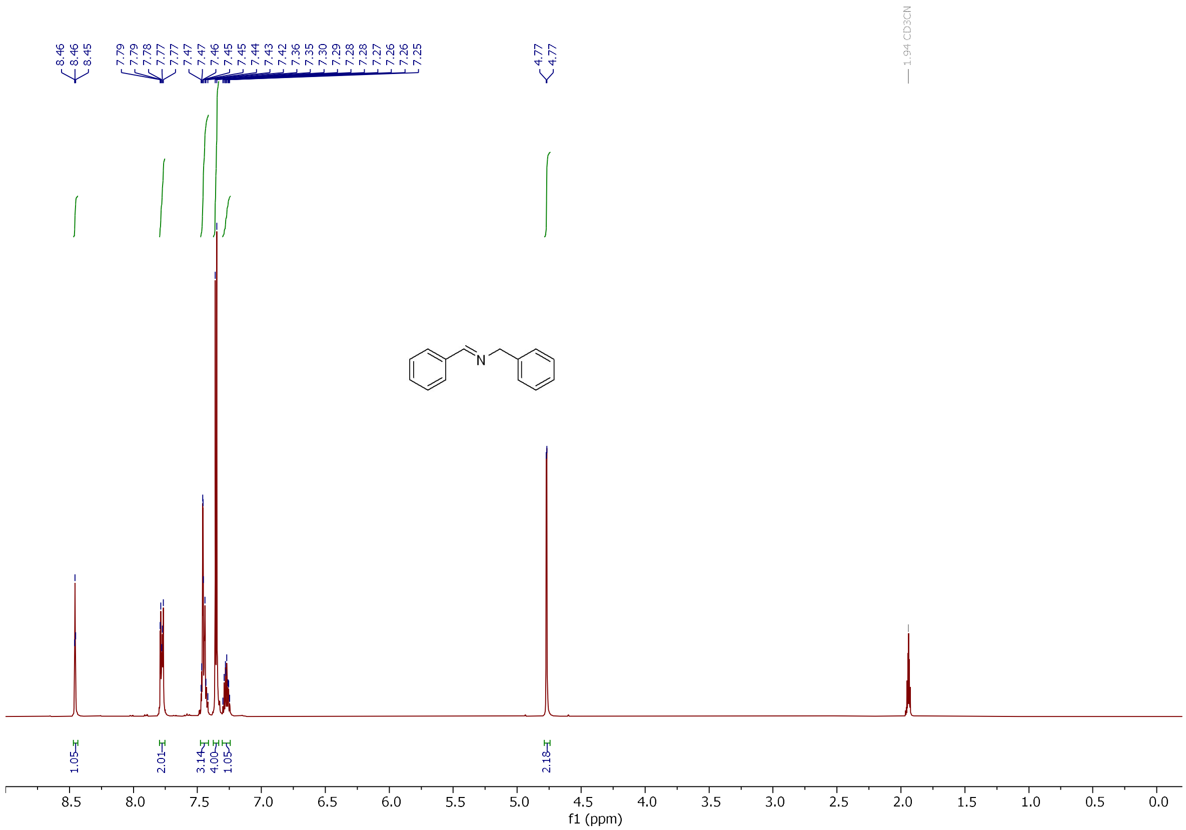

Supplementary Fig. 19. **Imine 1a ^{1}H NMR spectrum in CD_3CN.**

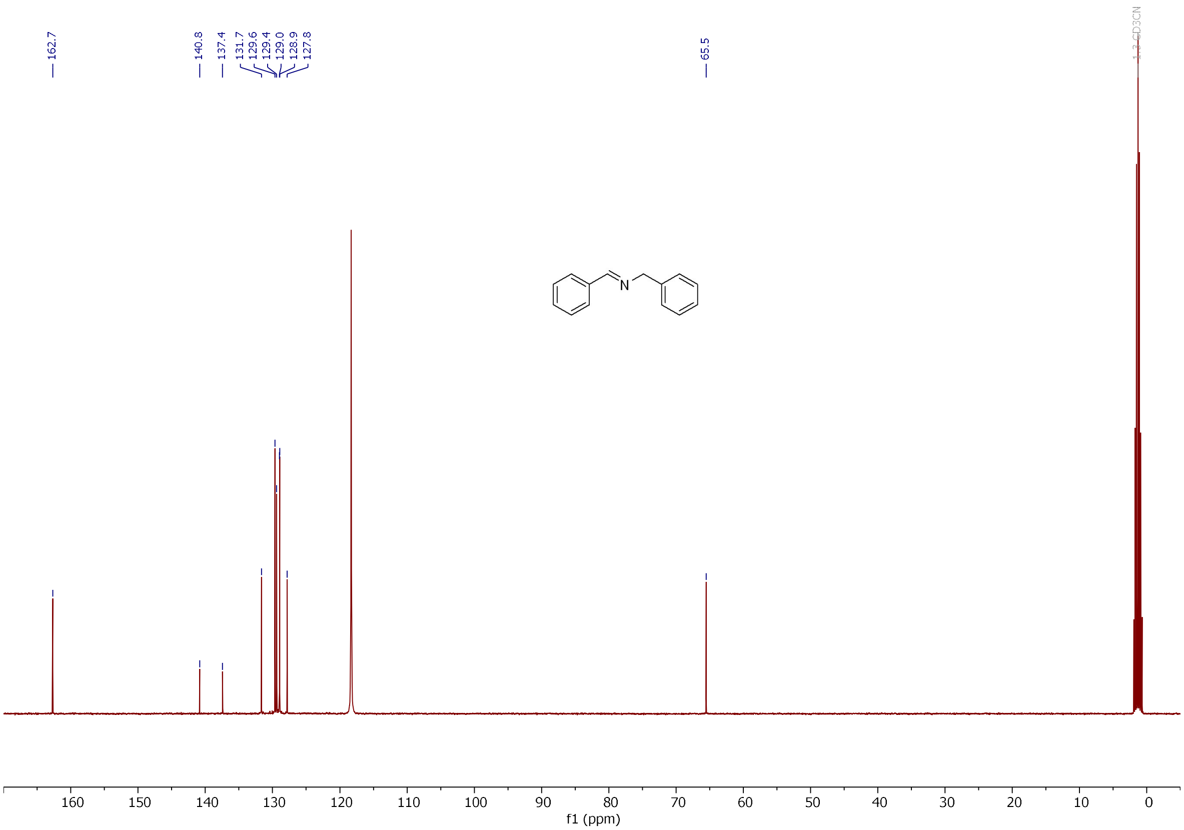

Supplementary Fig. 20**. Imine 1a ^{13}C NMR spectrum in CD_3CN**.

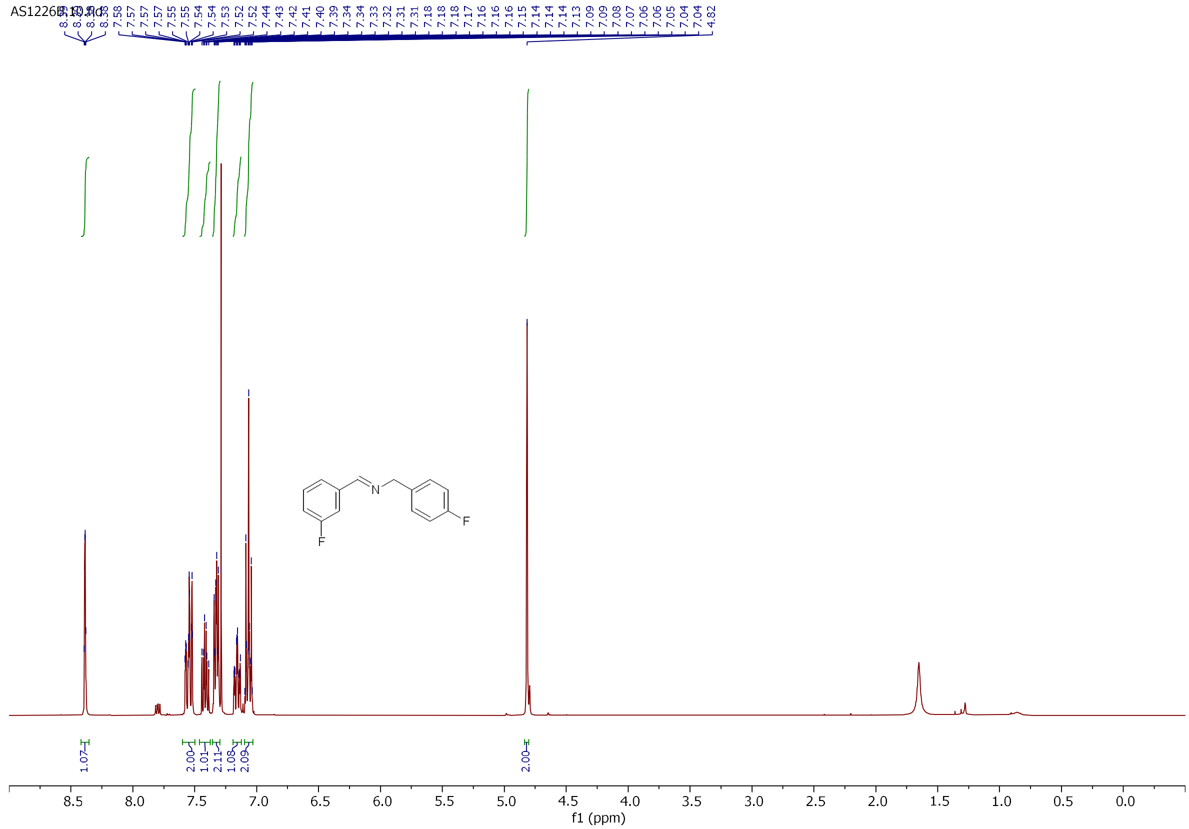

Supplementary Fig. 21. **Imine 1b ^{1}H NMR spectrum in $CDCl_3$.**

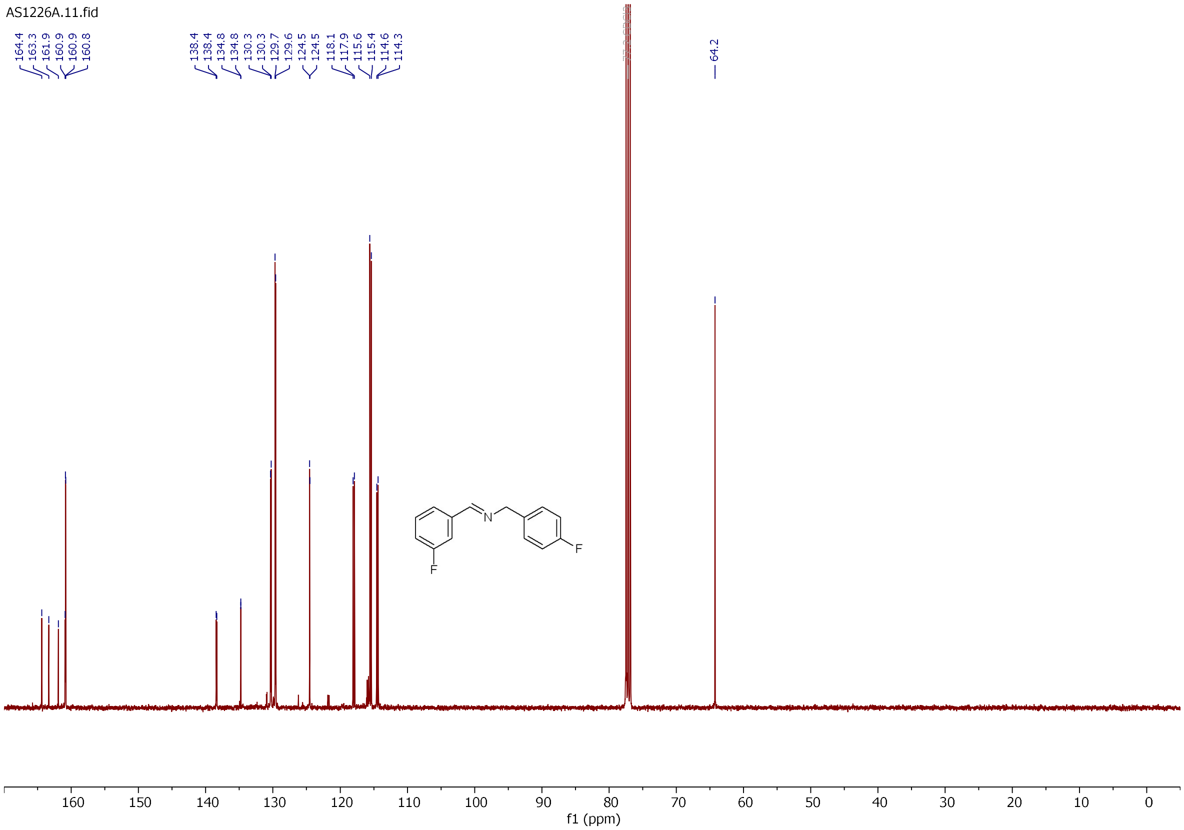

Supplementary Fig. 22. **Imine 1b ^{1}H NMR spectrum in $CDCl_3$.**

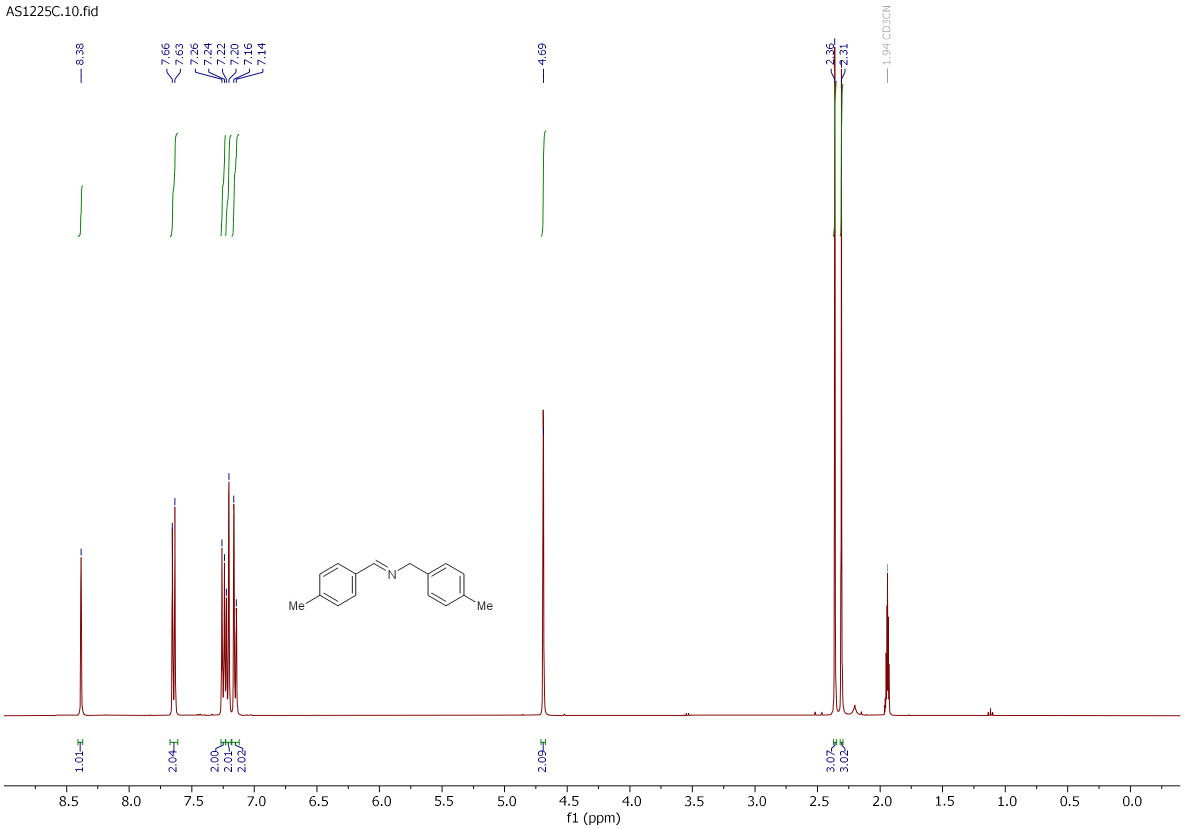

Supplementary Fig. 23. **Imine 1c ^{1}H NMR spectrum in CD_3CN.**

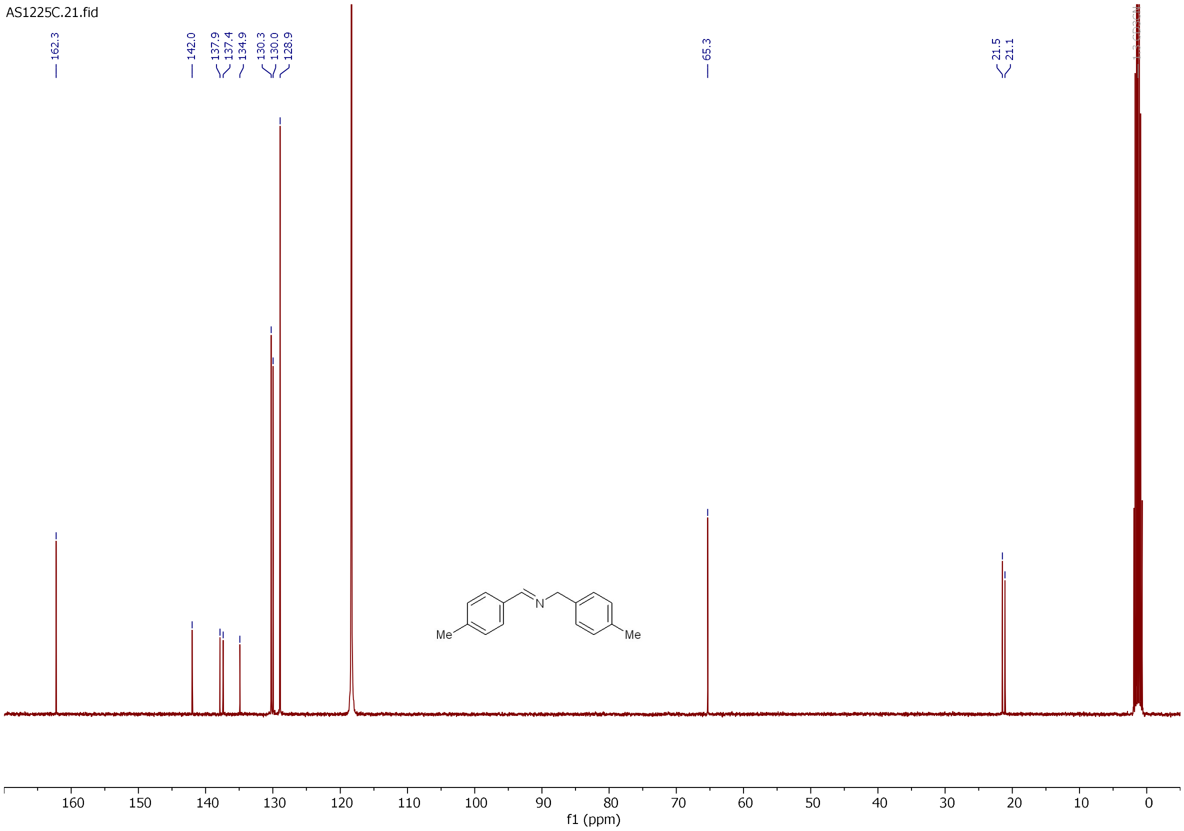

Supplementary Fig. 24. **Imine 1c ^{13}C NMR spectrum in CD_3CN**.

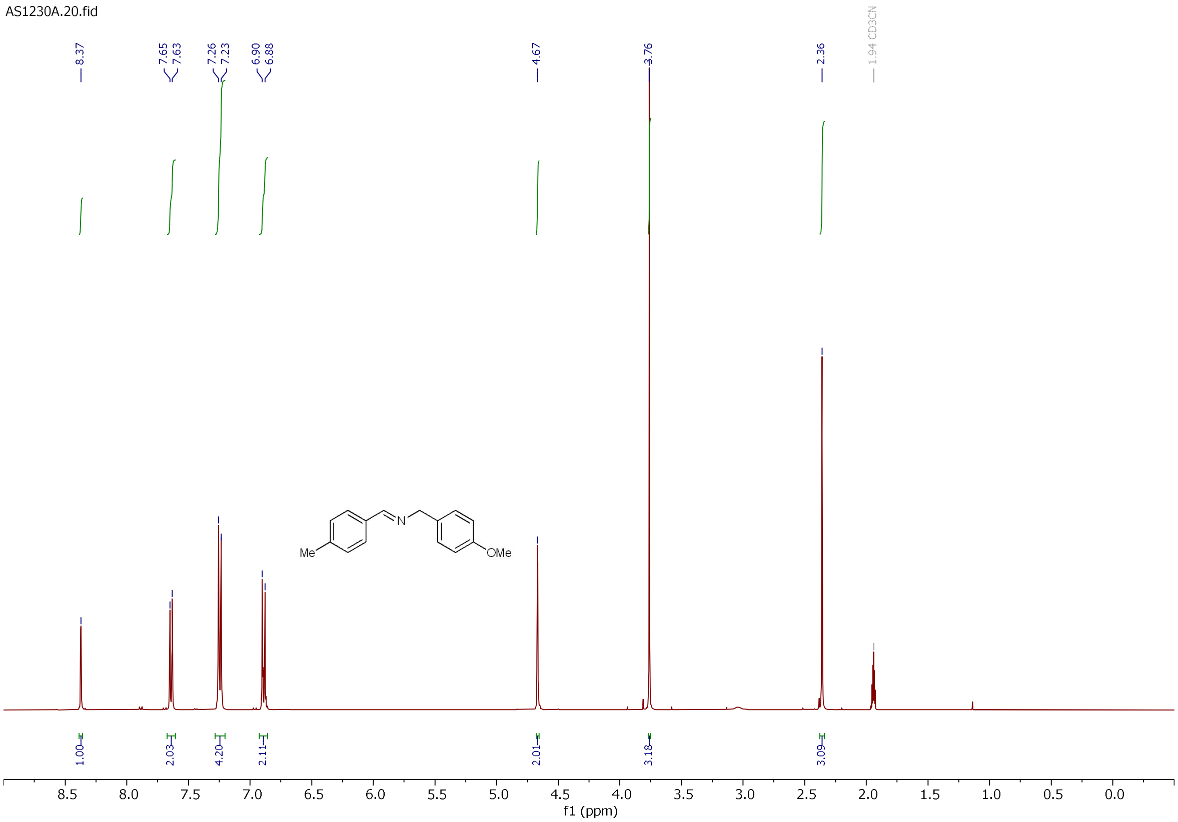

Supplementary Fig. 25. **Imine 1d ^{1}H NMR spectrum in CD_3CN.**

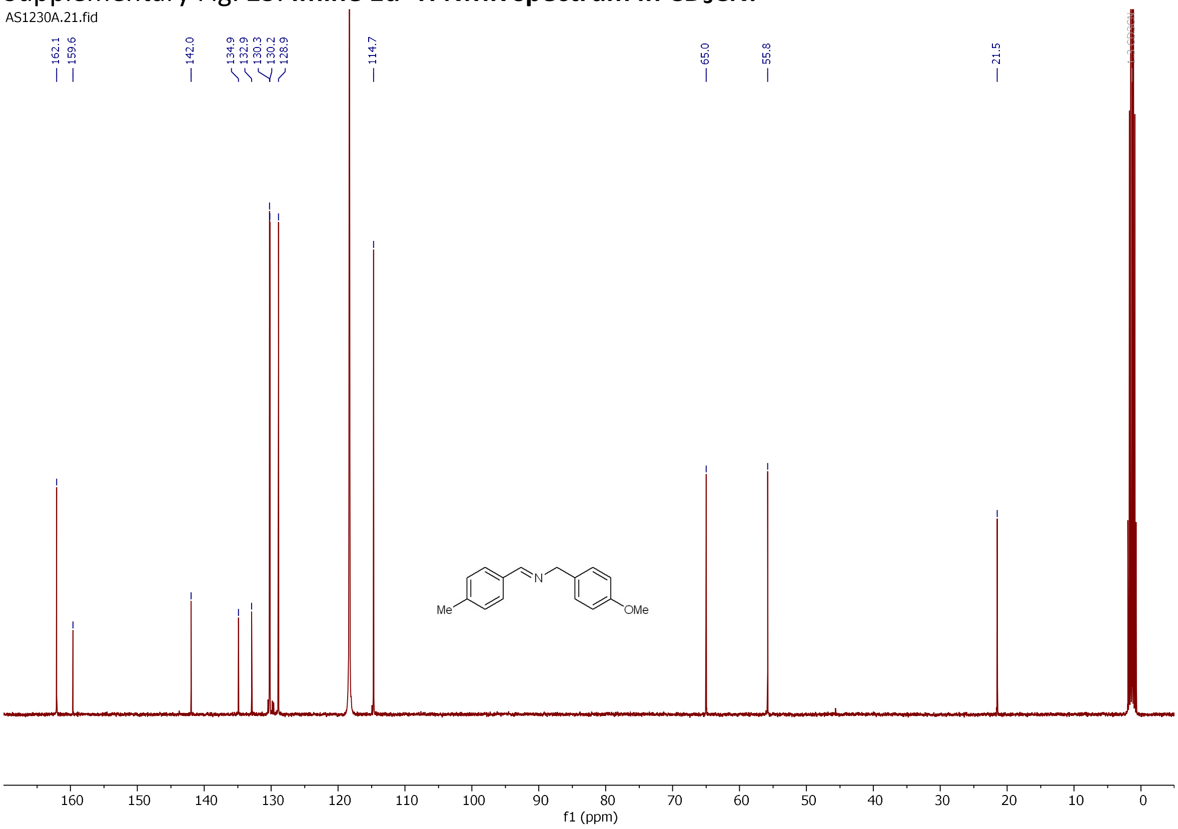

Supplementary Fig. 26. Imine 1d ^{13}C NMR spectrum in CD_3CN.

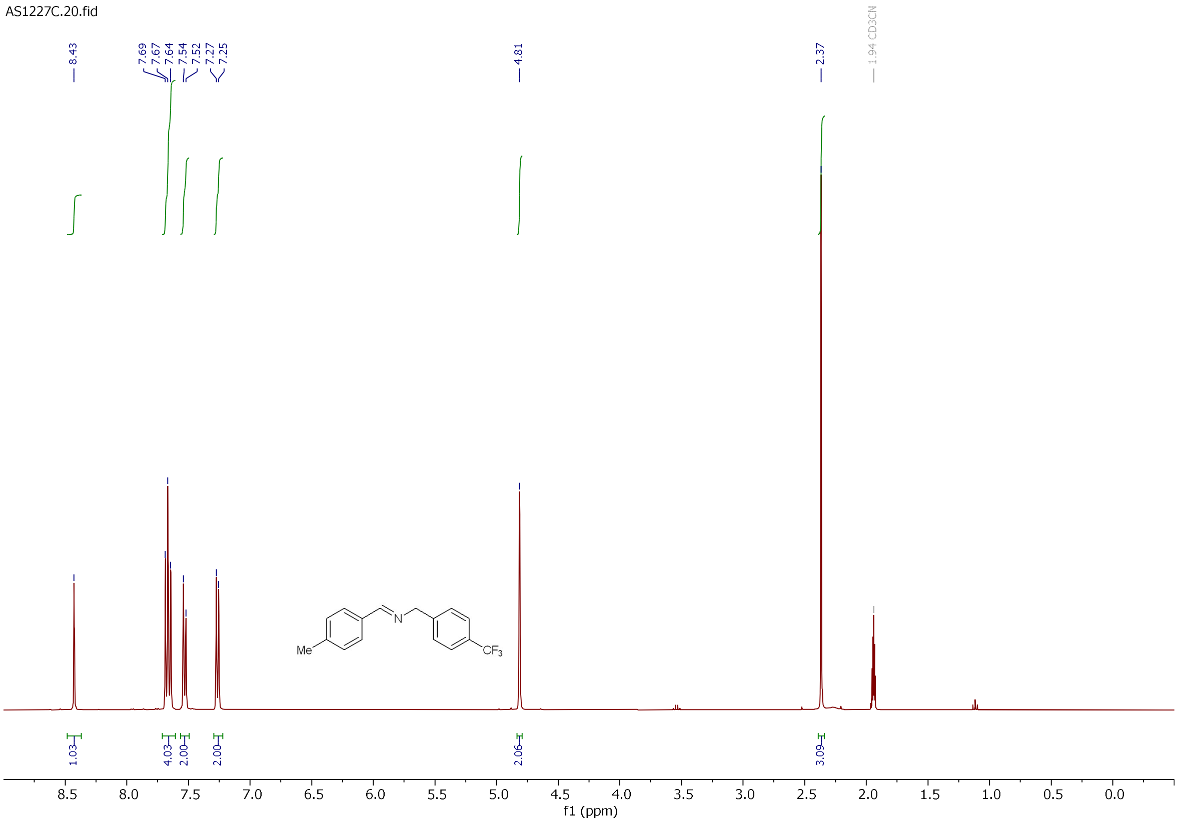

Supplementary Fig. 27. **Imine 1e ^{1}H NMR spectrum in CD_3CN.**

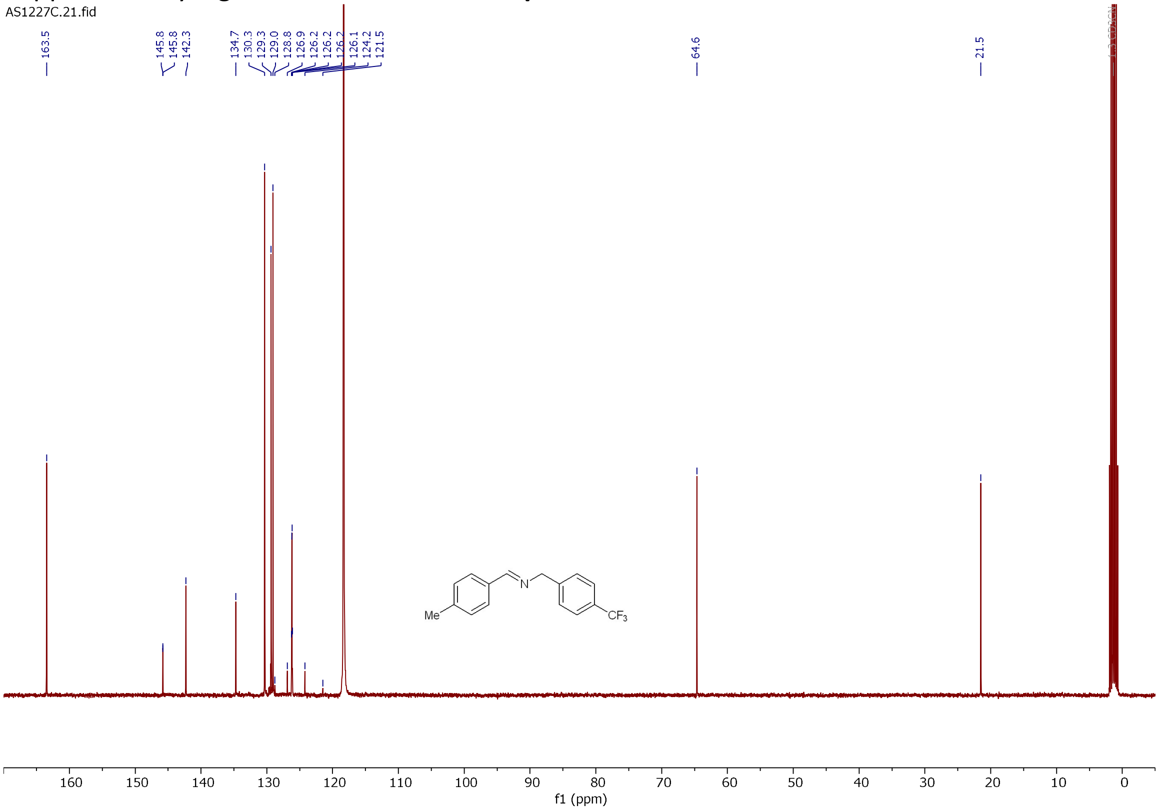

Supplementary Fig. 28. Imine 1e ^{13}C NMR spectrum in CD_3CN.

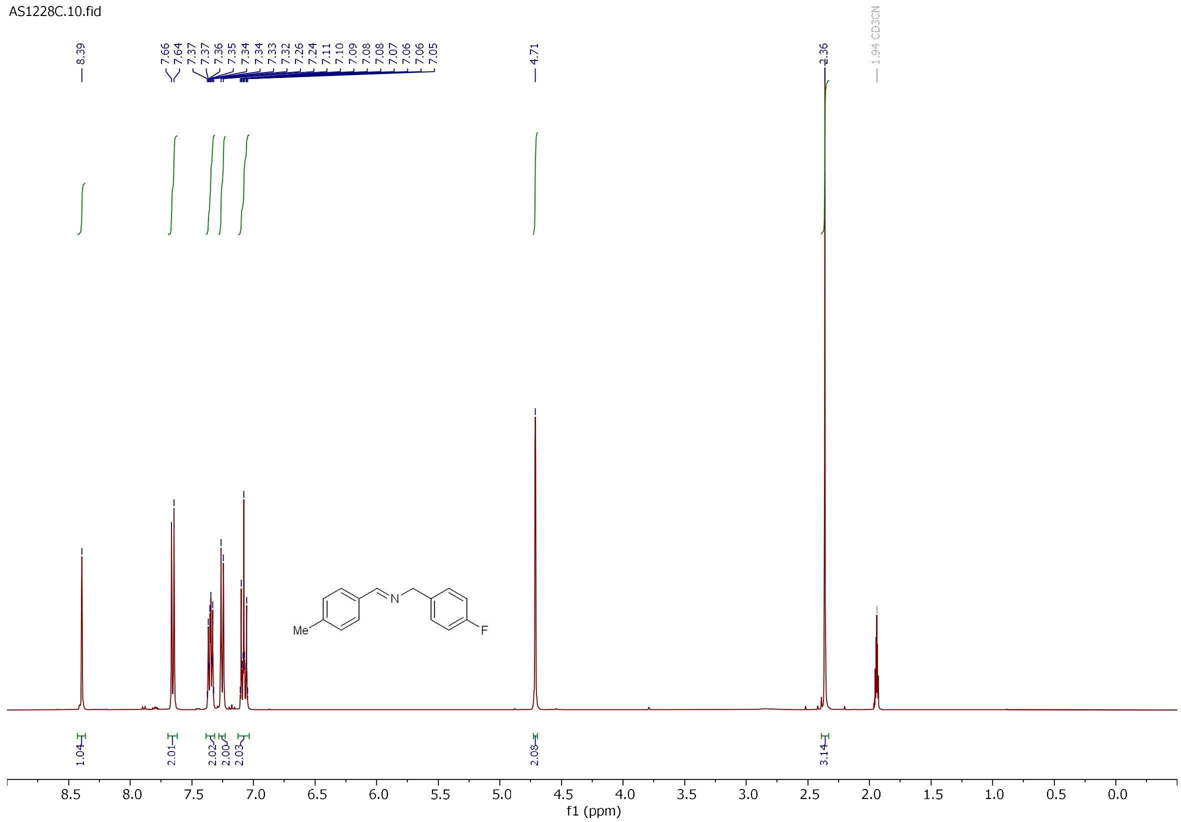

Supplementary Fig. 29. **Imine 1f ^{1}H NMR spectrum in CD_3CN.**

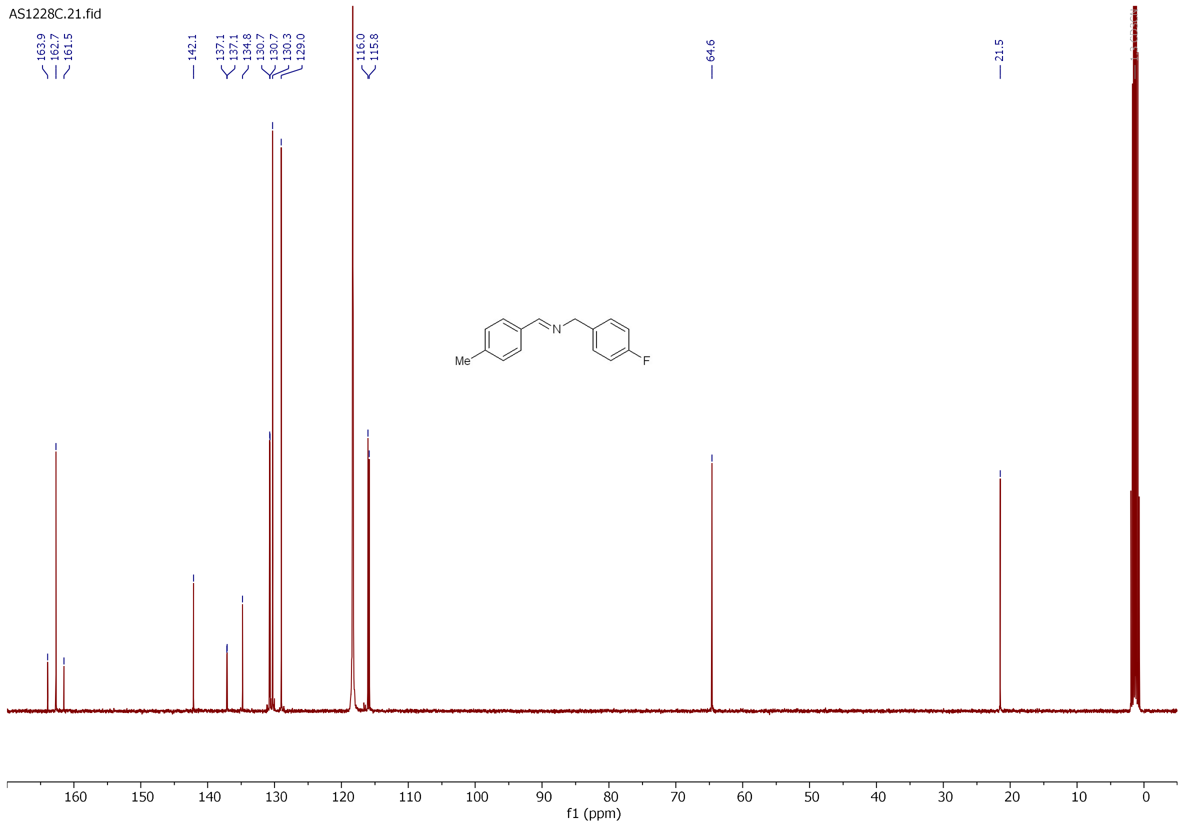

Supplementary Fig. 30. Imine 1f ^{13}C NMR spectrum in CD_3CN.

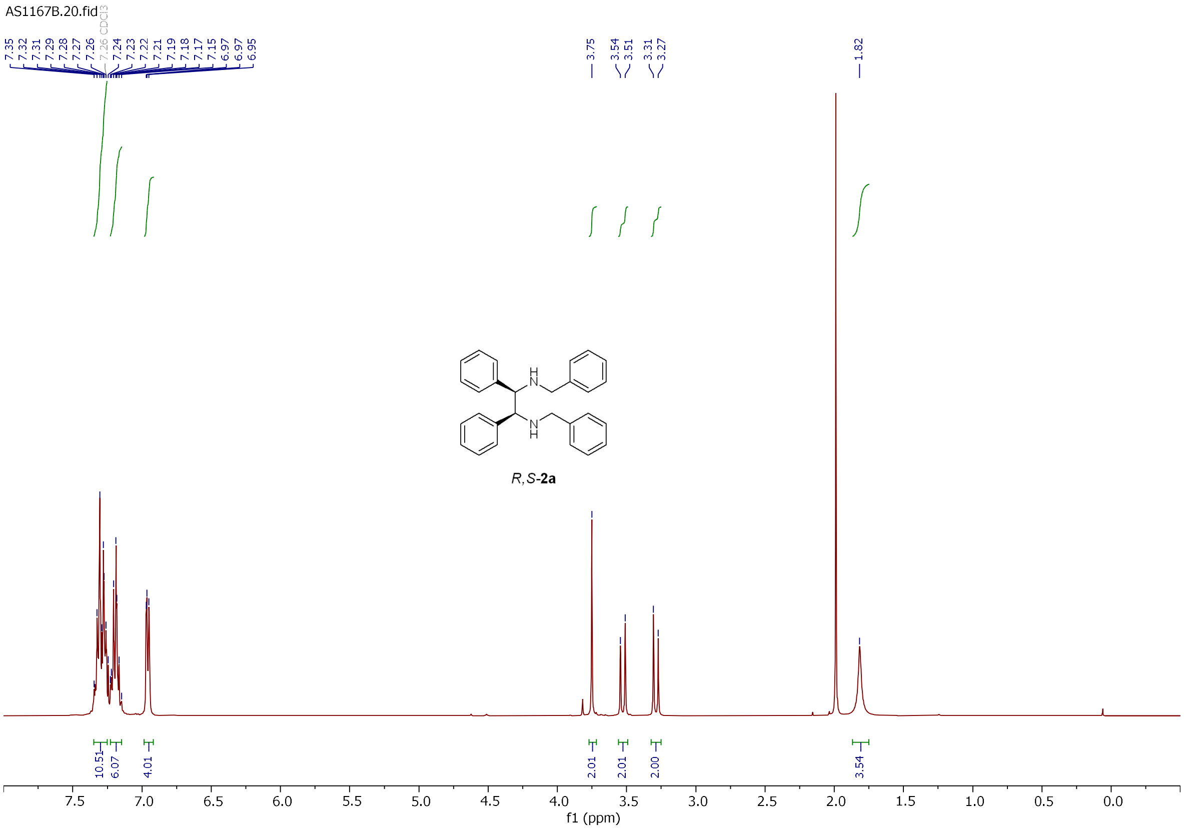

Supplementary Fig. 31. ***R,S*-2a ^{1}H NMR spectrum in $CDCl_3$.**

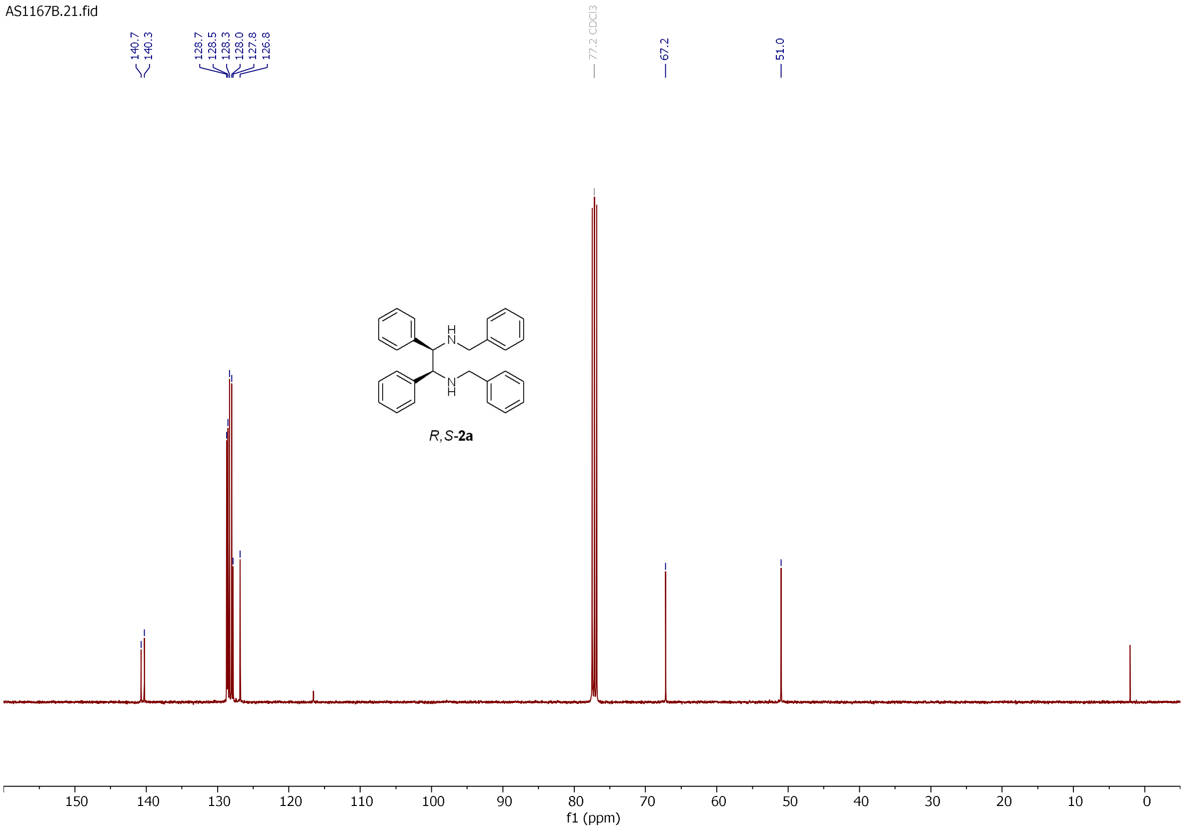

Supplementary Fig. 32. *R,S*-2a ^{13}C NMR spectrum in $CDCl_3$.

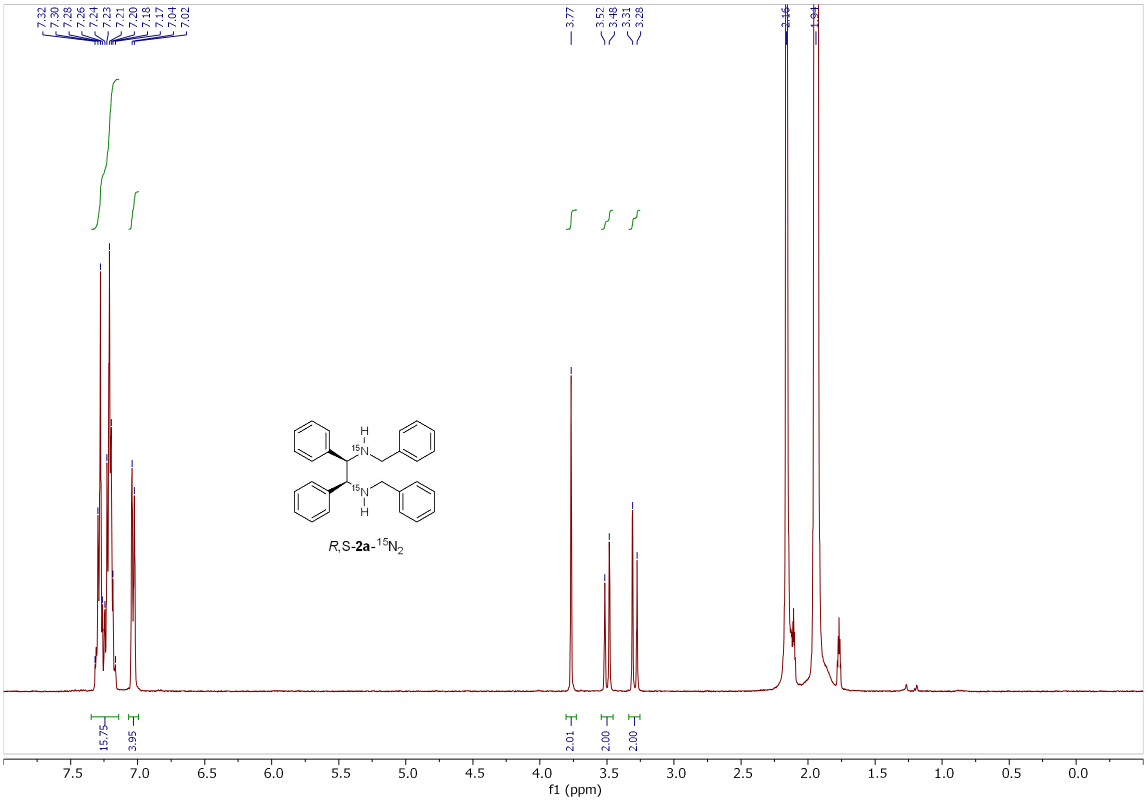

Supplementary Fig. 33. ***R,S*-2a-$^{15}N_2$ ^{1}H NMR spectrum in CD_3CN.**

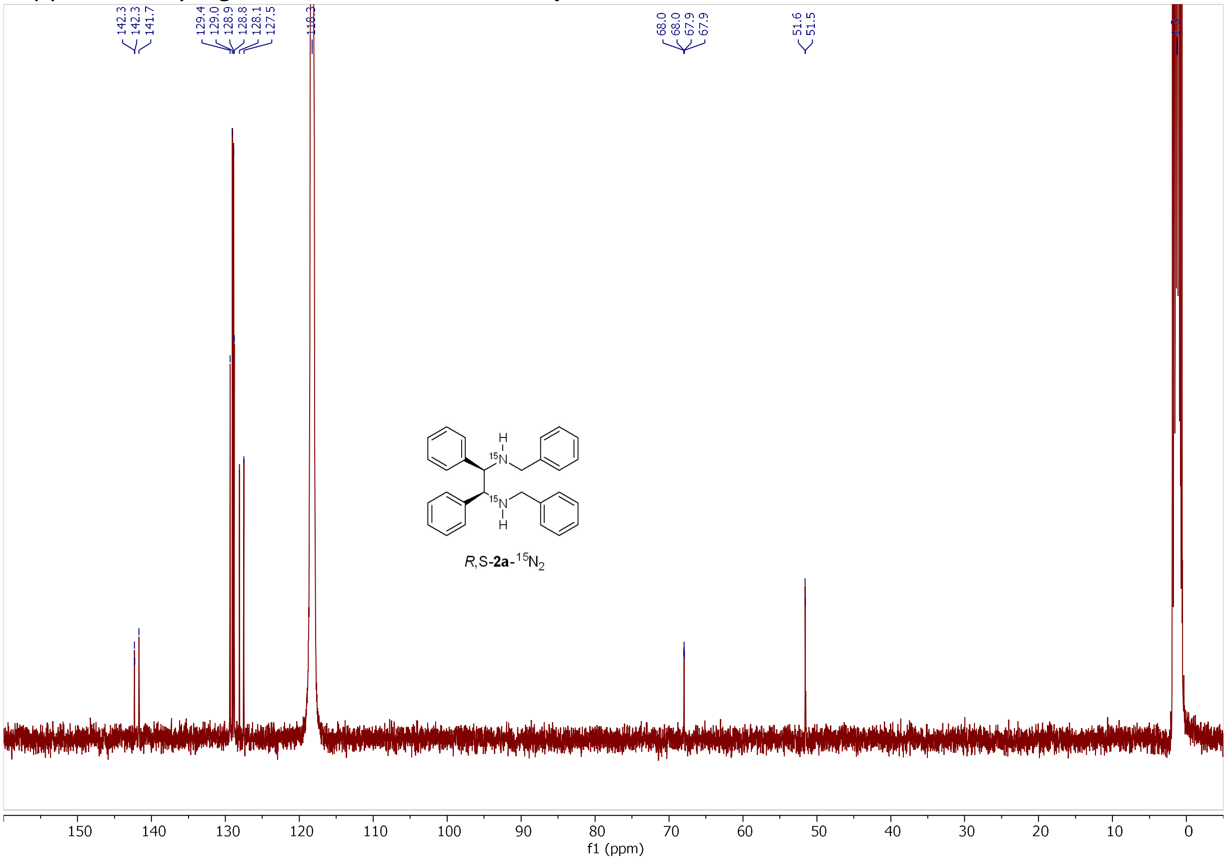

Supplementary Fig. 34. *R,S*-2a-$^{15}N_2$ ^{13}C NMR spectrum in CD_3CN.

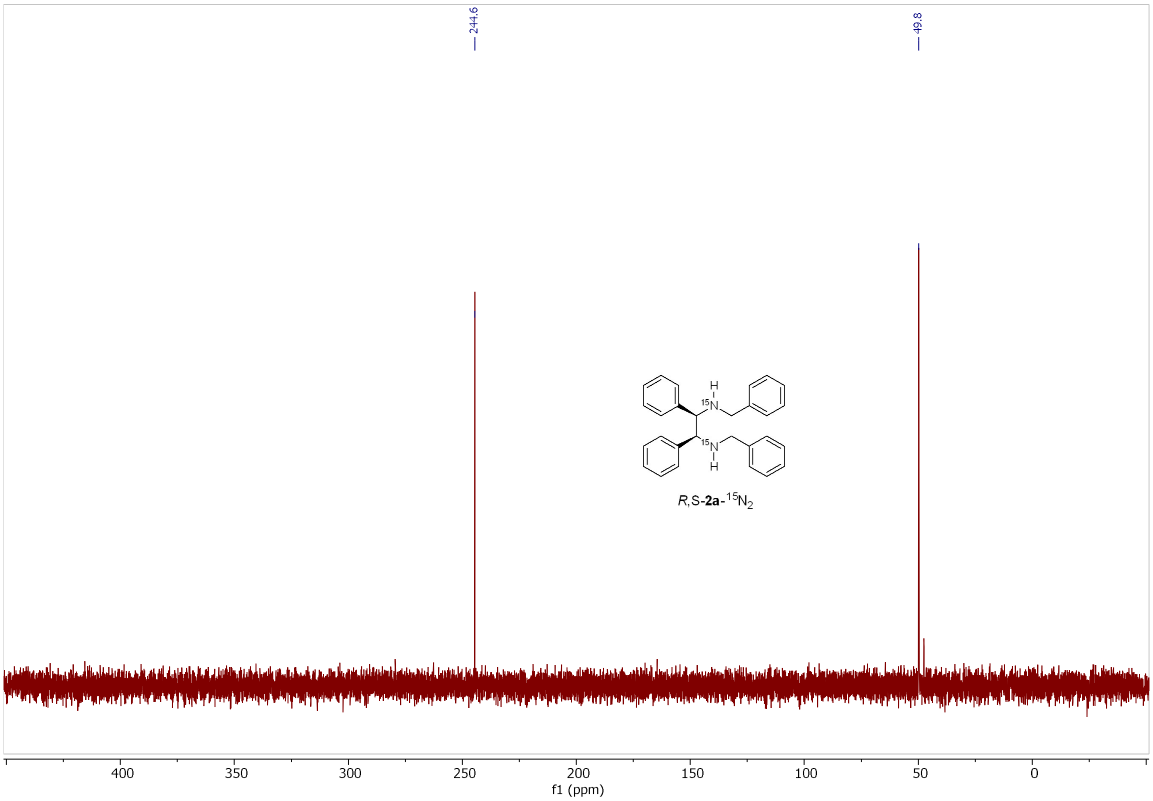

Supplementary Fig. 35. ***R,S*-2a-$^{15}N_2$ ^{15}N NMR spectrum in CD_3CN.**

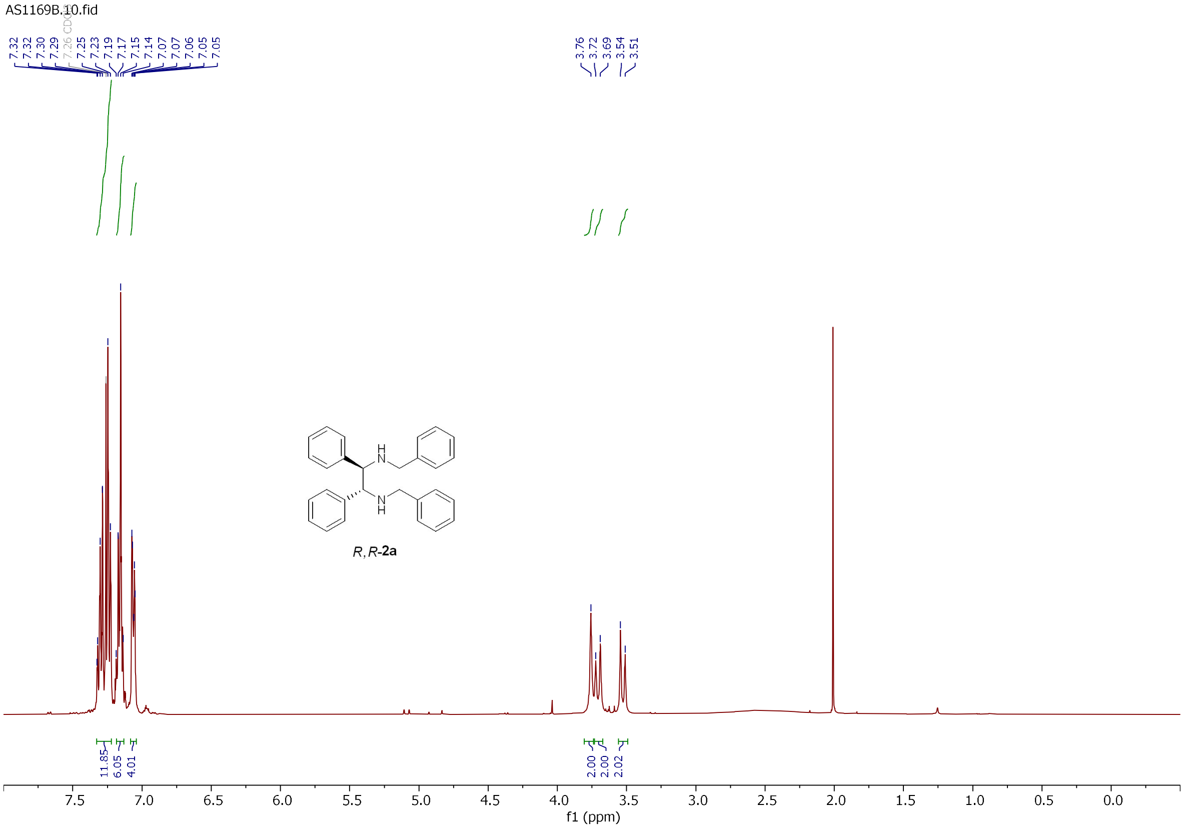

Supplementary Fig. 36. *R,R*-2a ^{1}H NMR spectrum in $CDCl_3$.

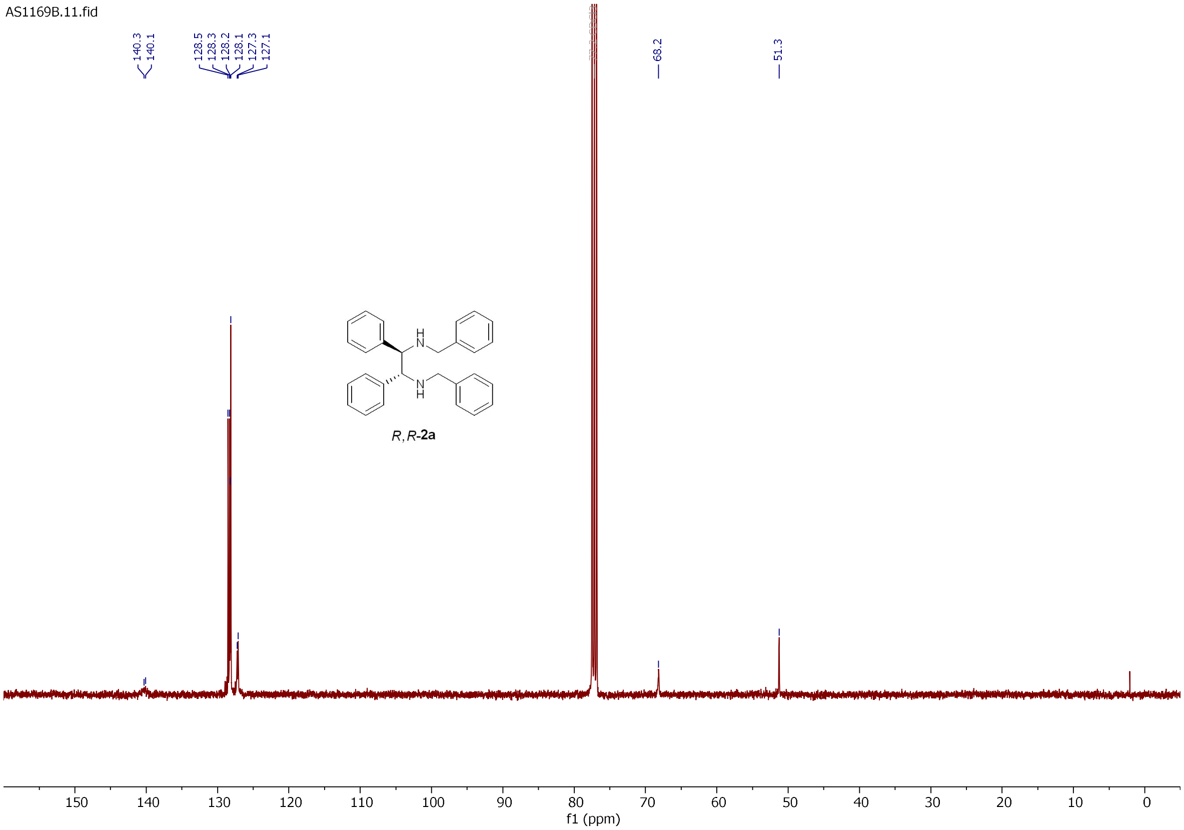

Supplementary Fig. 37. ***R,R*-2a ^{13}C NMR spectrum in $CDCl_3$.**

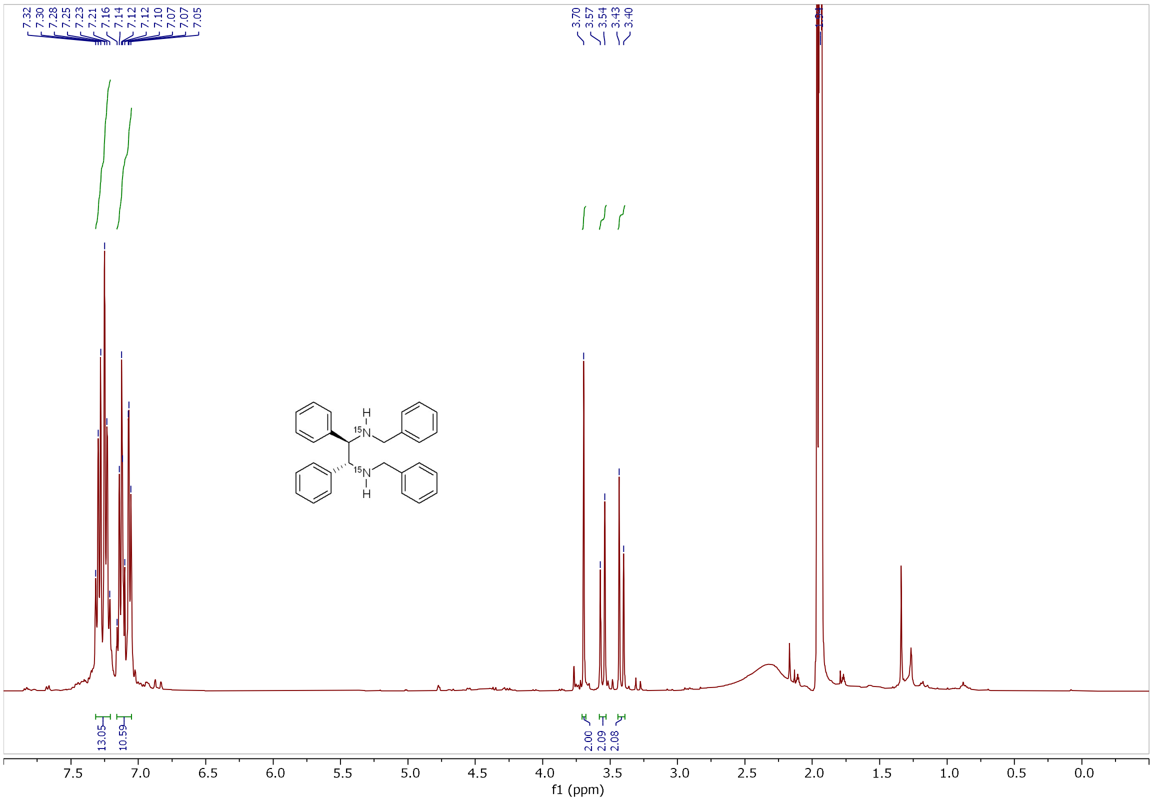

Supplementary Fig. 38. ***R,R*-2a-$^{15}N_2$ ^{1}H NMR spectrum in CD_3CN.**

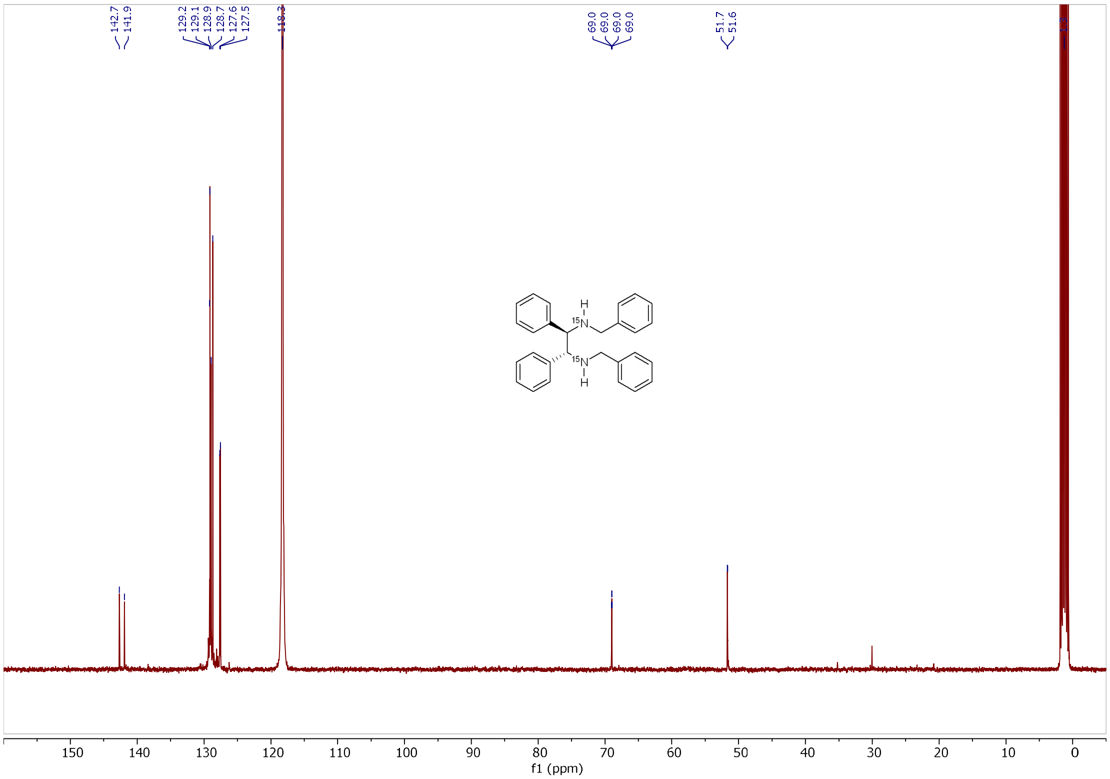

Supplementary Fig. 39. ***R*****,*****R*****-2a-$^{15}N_2$ ^{13}C NMR spectrum in CD_3CN.**

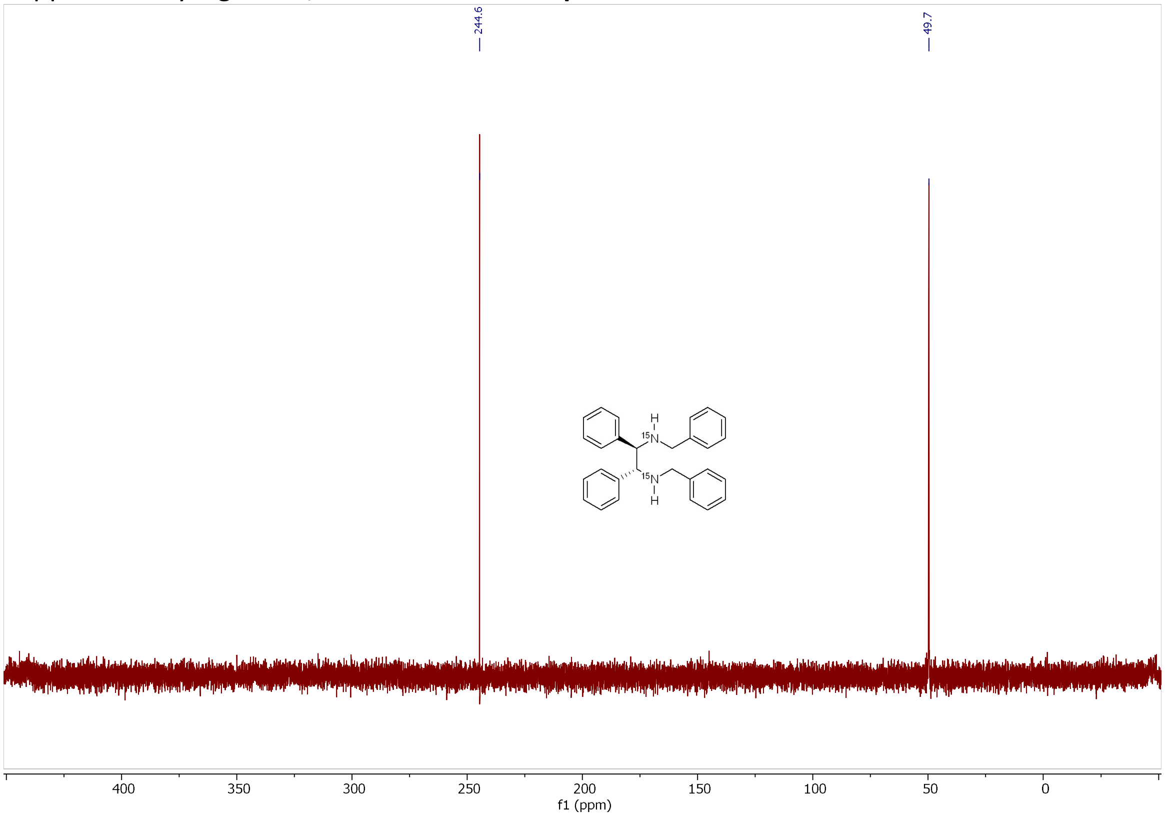

Supplementary Fig. 40. *R*,*R*-2a-$^{15}N_2$ ^{15}N NMR spectrum in CD_3CN.

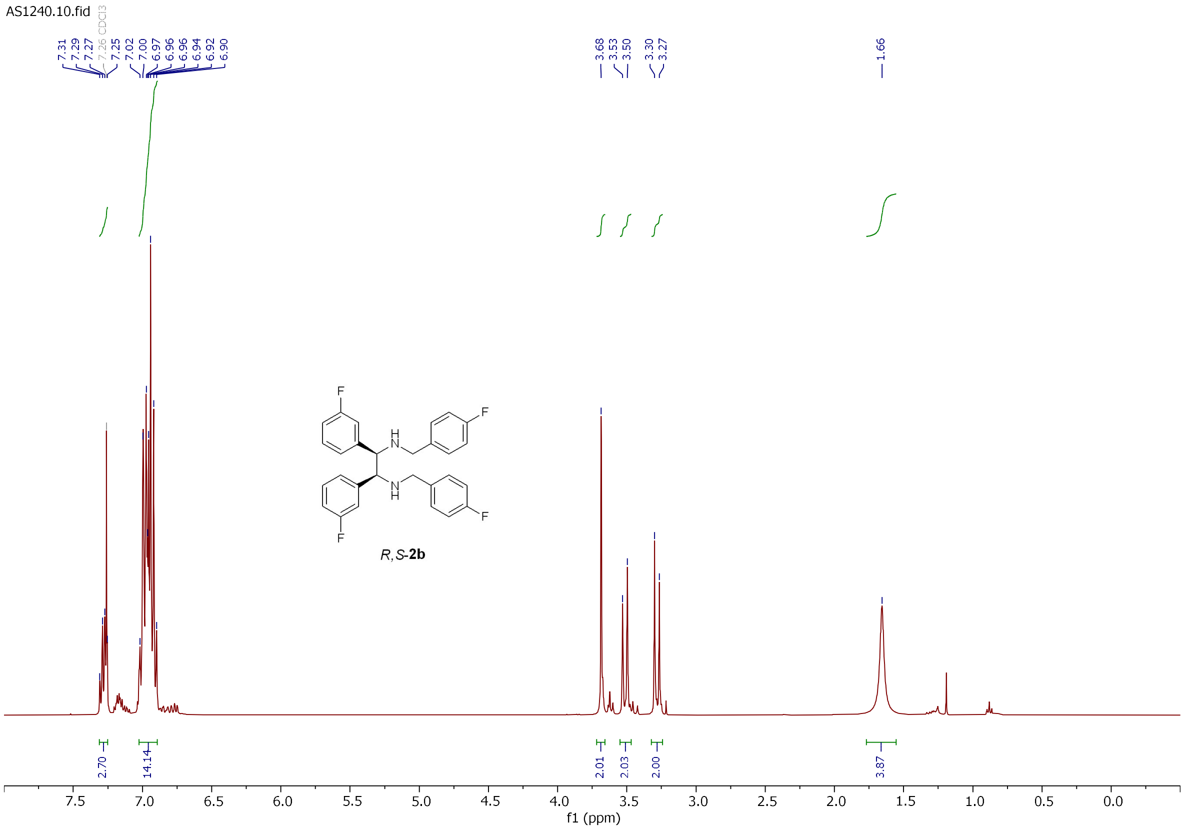

Supplementary Fig. 41. ***R,S*-2b ^{1}H NMR spectrum in $CDCl_3$.**

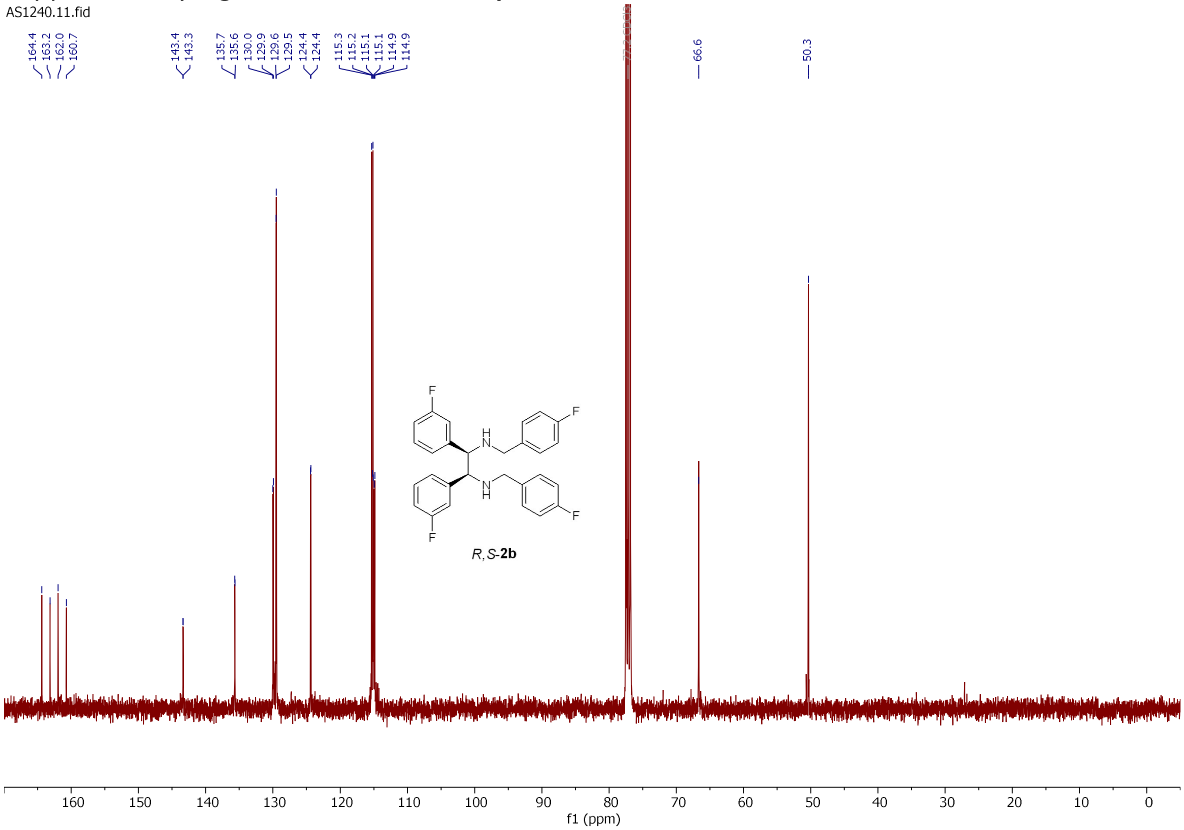

Supplementary Fig. 42. *R,S*-2b ^{13}C NMR spectrum in $CDCl_3$.

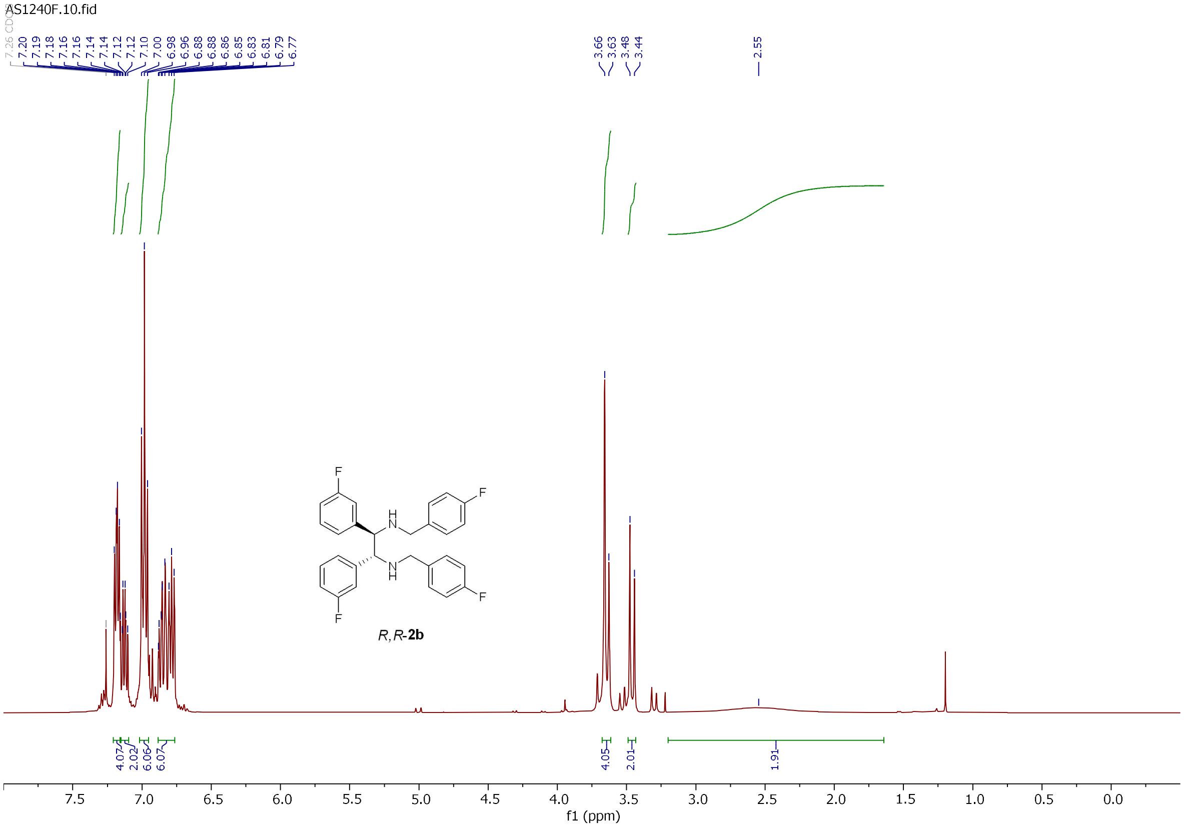

Supplementary Fig. 43. ***R,R*-2b ^{1}H NMR spectrum in $CDCl_3$.**

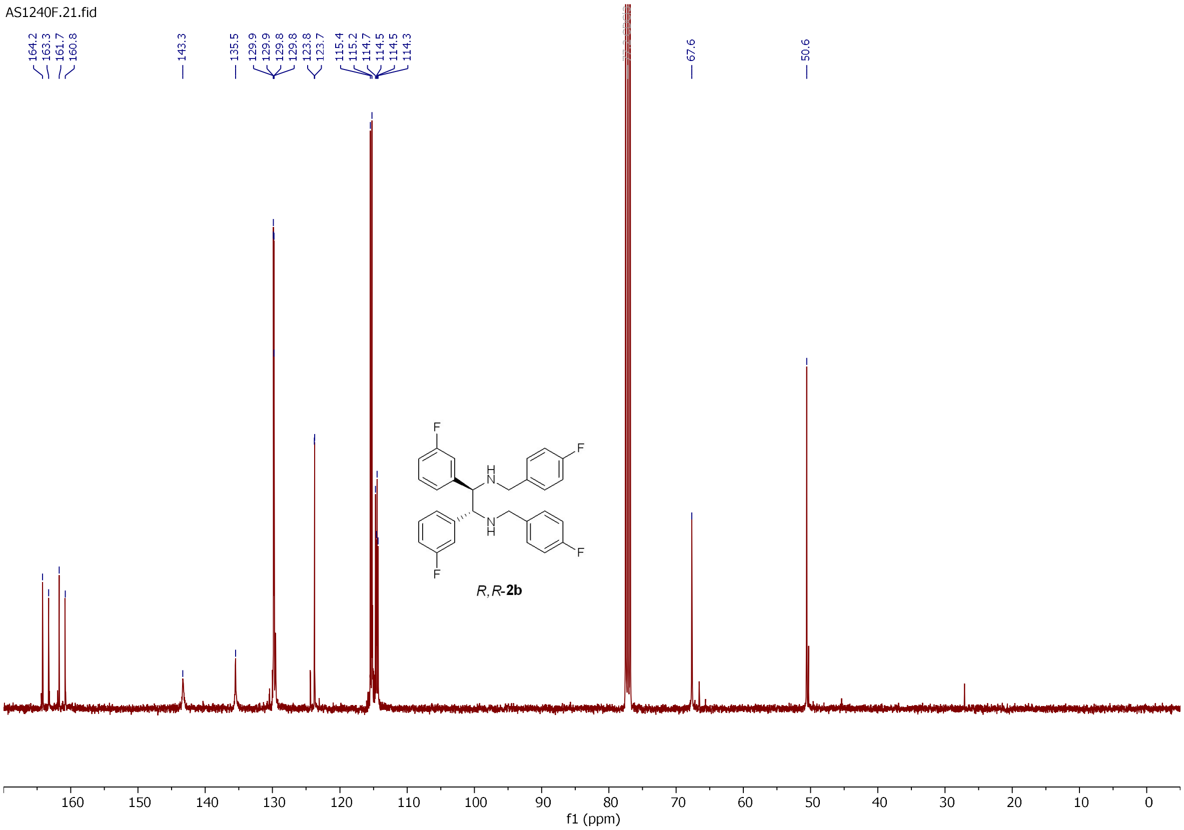

Supplementary Fig. 44. *R*,*R*-2b ^{13}C NMR spectrum in $CDCl_3$.

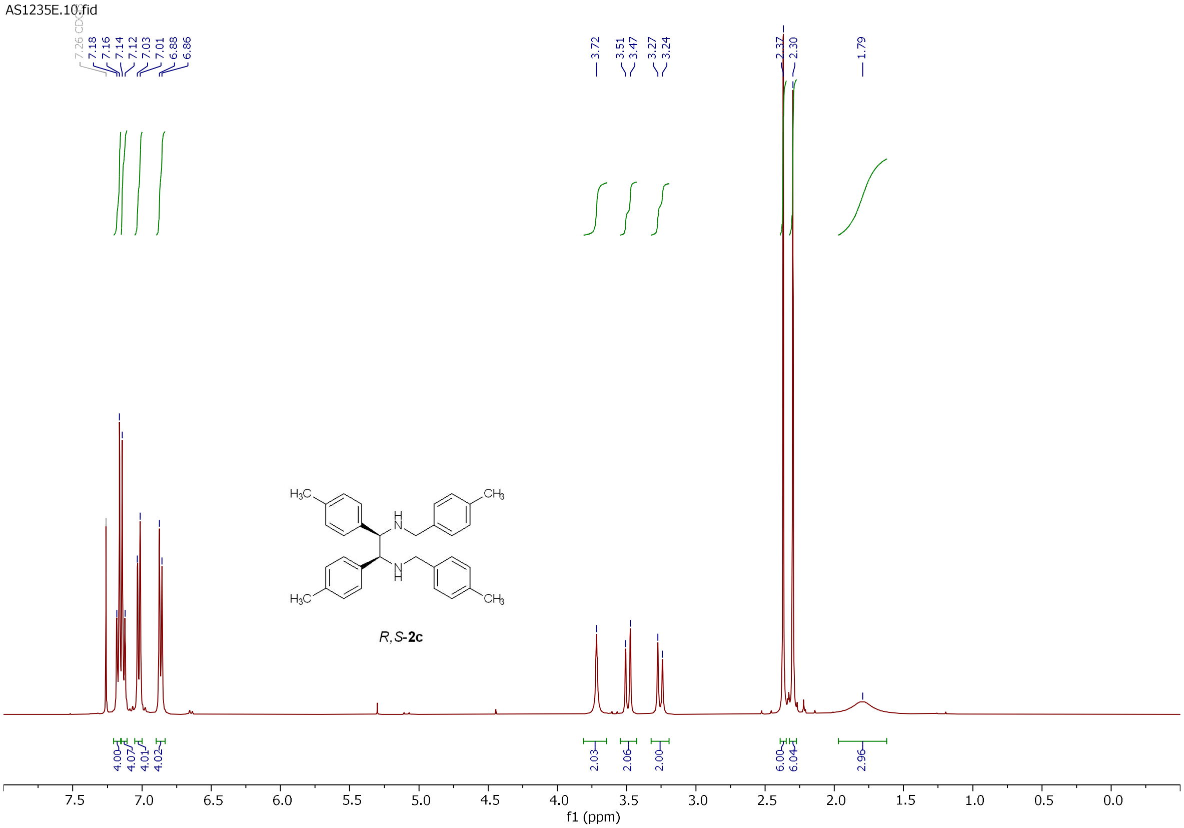

Supplementary Fig. 45. ***R,S*-2c ^{1}H NMR spectrum in $CDCl_3$.**

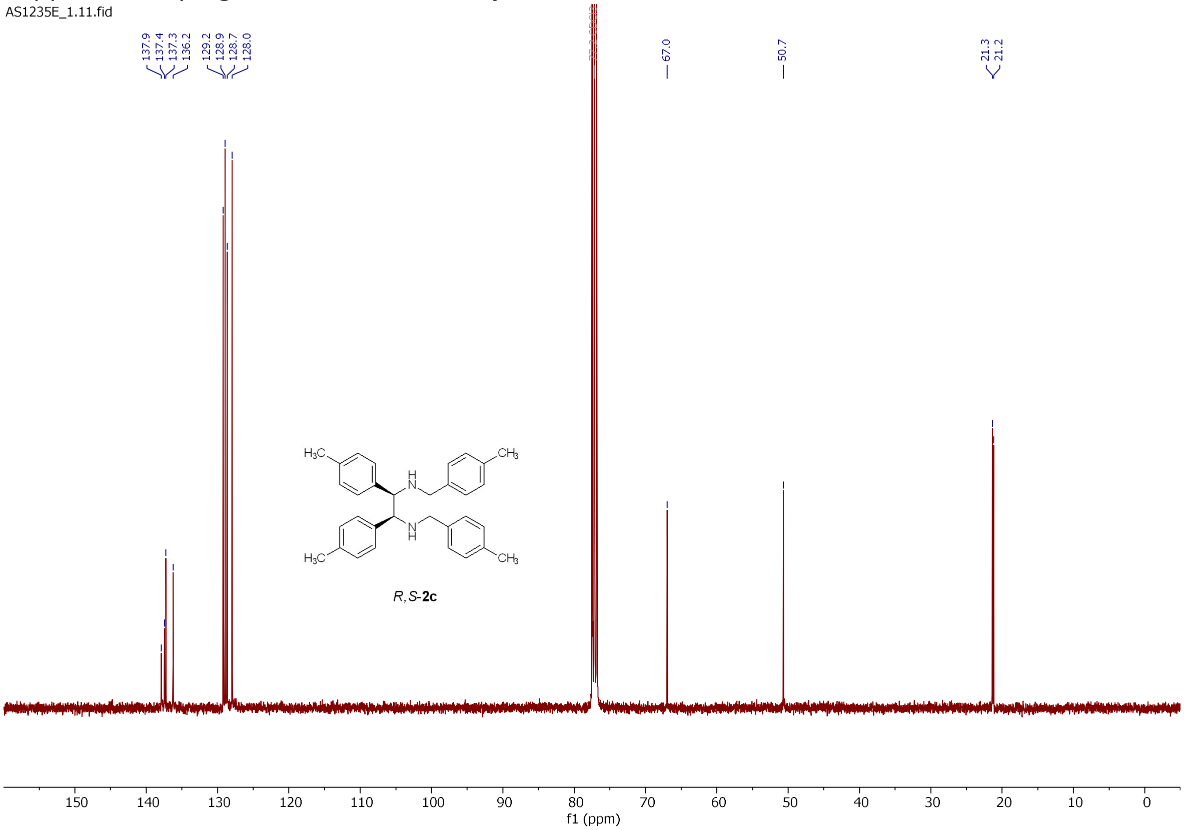

Supplementary Fig. 46. *R,S*-2c ^{13}C NMR spectrum in $CDCl_3$.

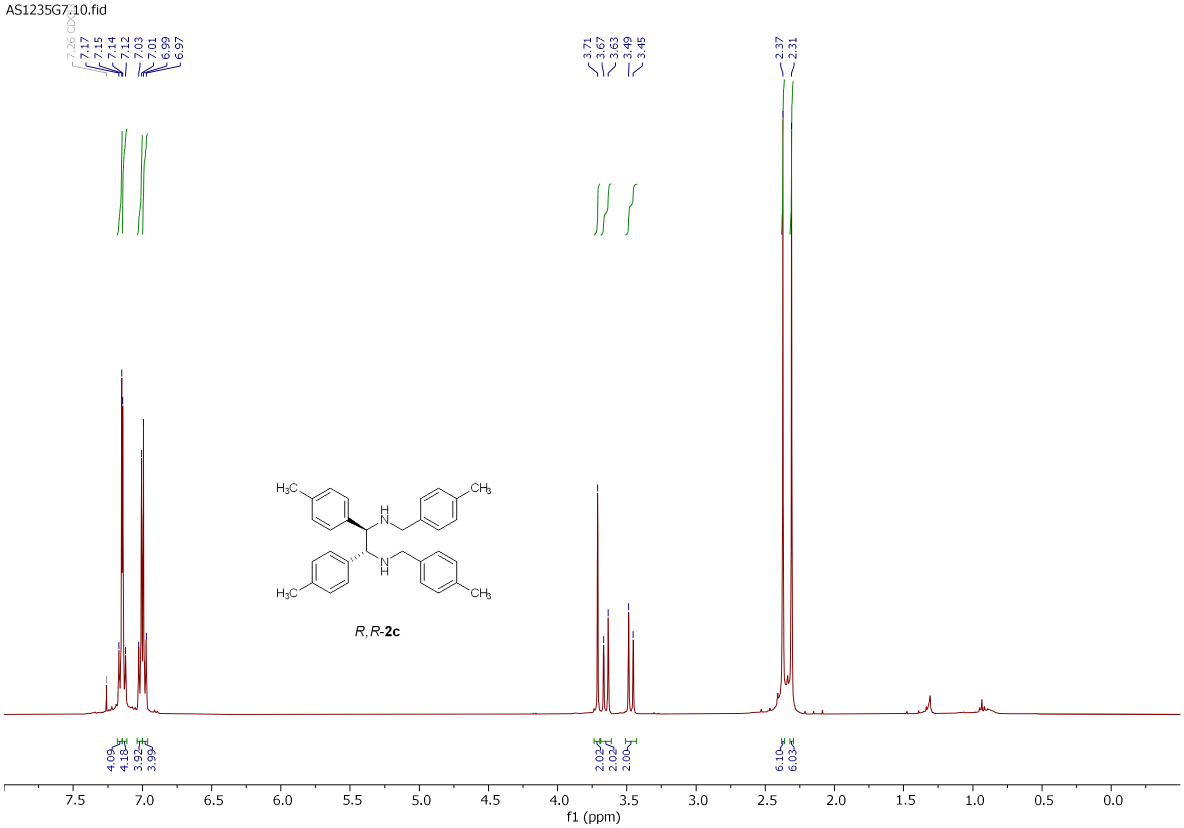

Supplementary Fig. 47. ***R,R*-2c ^{1}H NMR spectrum in $CDCl_3$.**

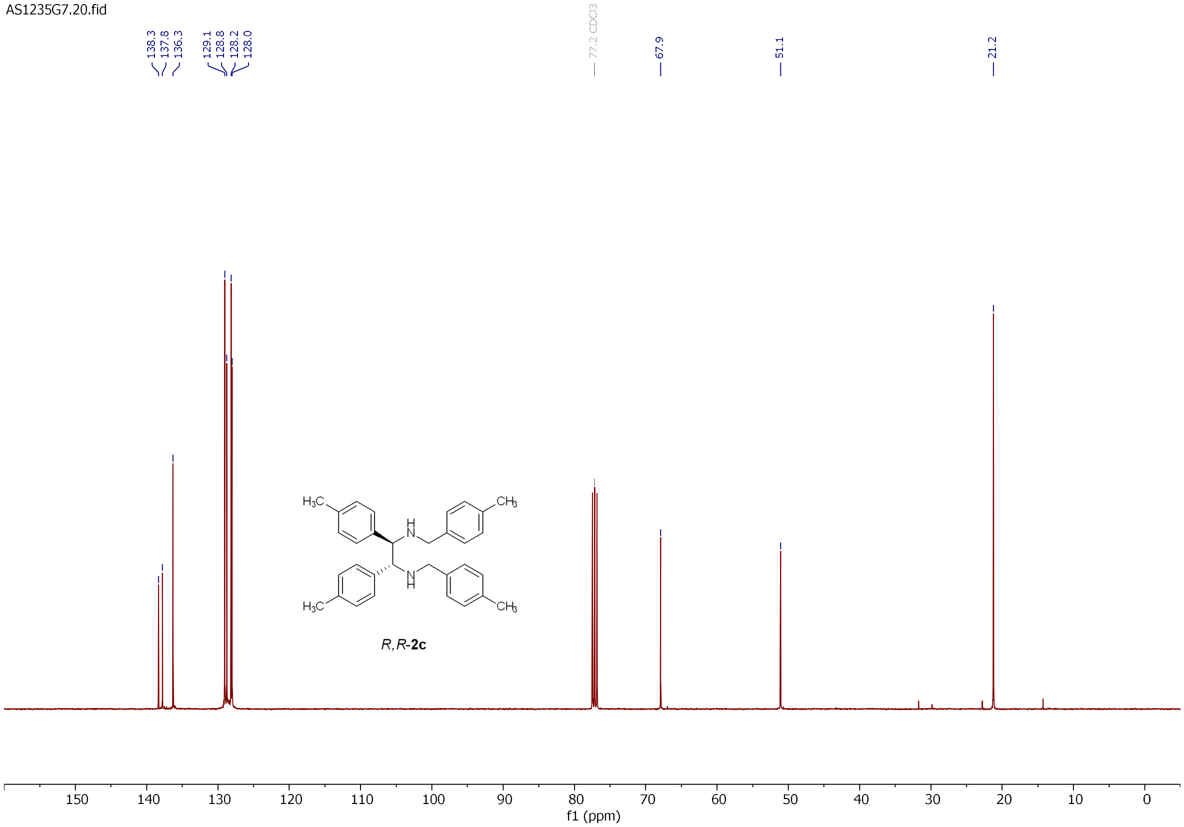

Supplementary Fig. 48. *R*,*R*-2c ^{13}C NMR spectrum in $CDCl_3$.

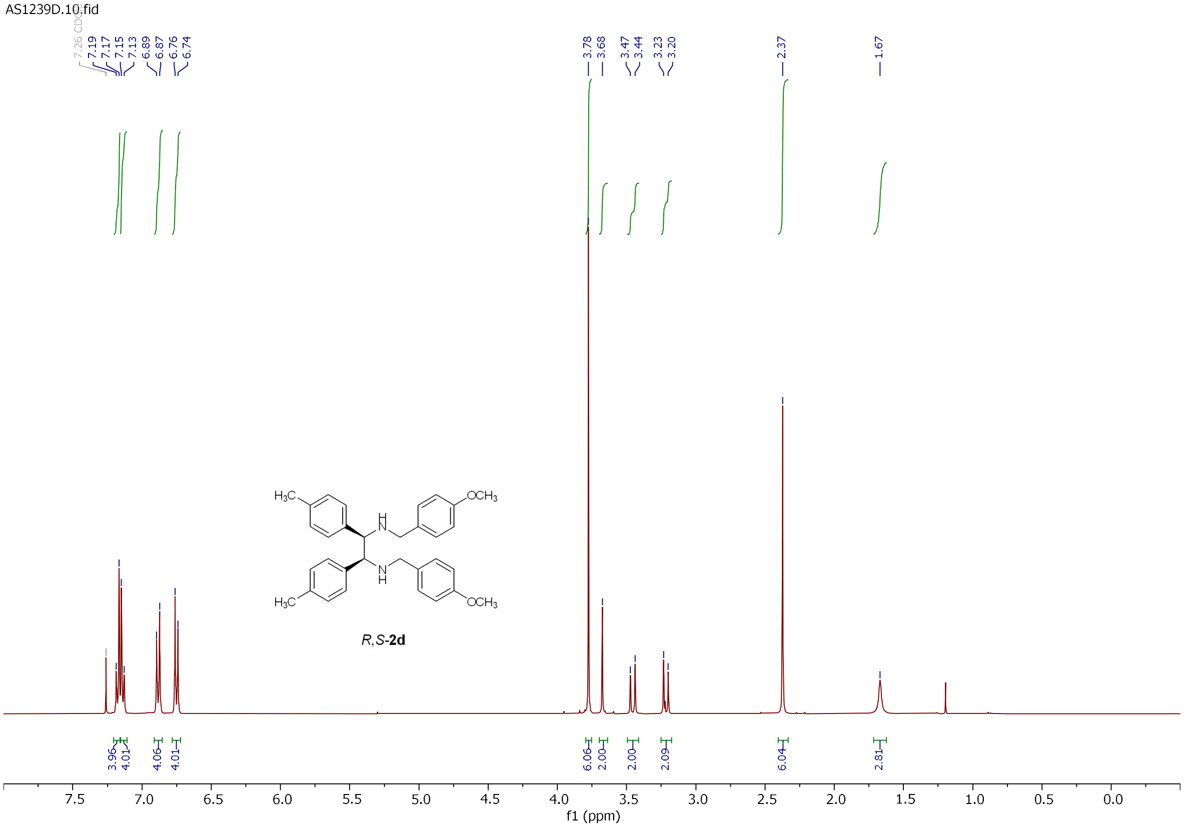

Supplementary Fig. 49. ***R*,*S*-2d ^{1}H NMR spectrum in $CDCl_3$.**

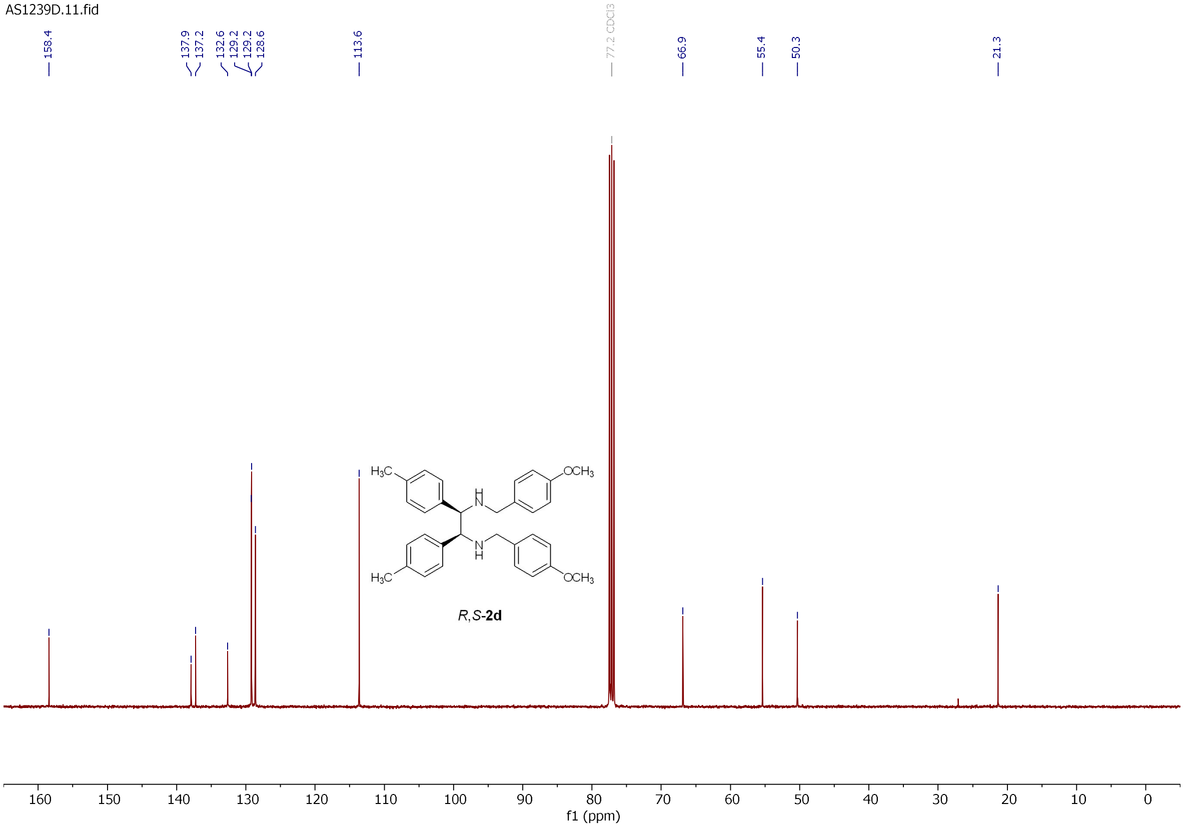

Supplementary Fig. 50. *R*,*S*-2d ^{13}C NMR spectrum in $CDCl_3$.

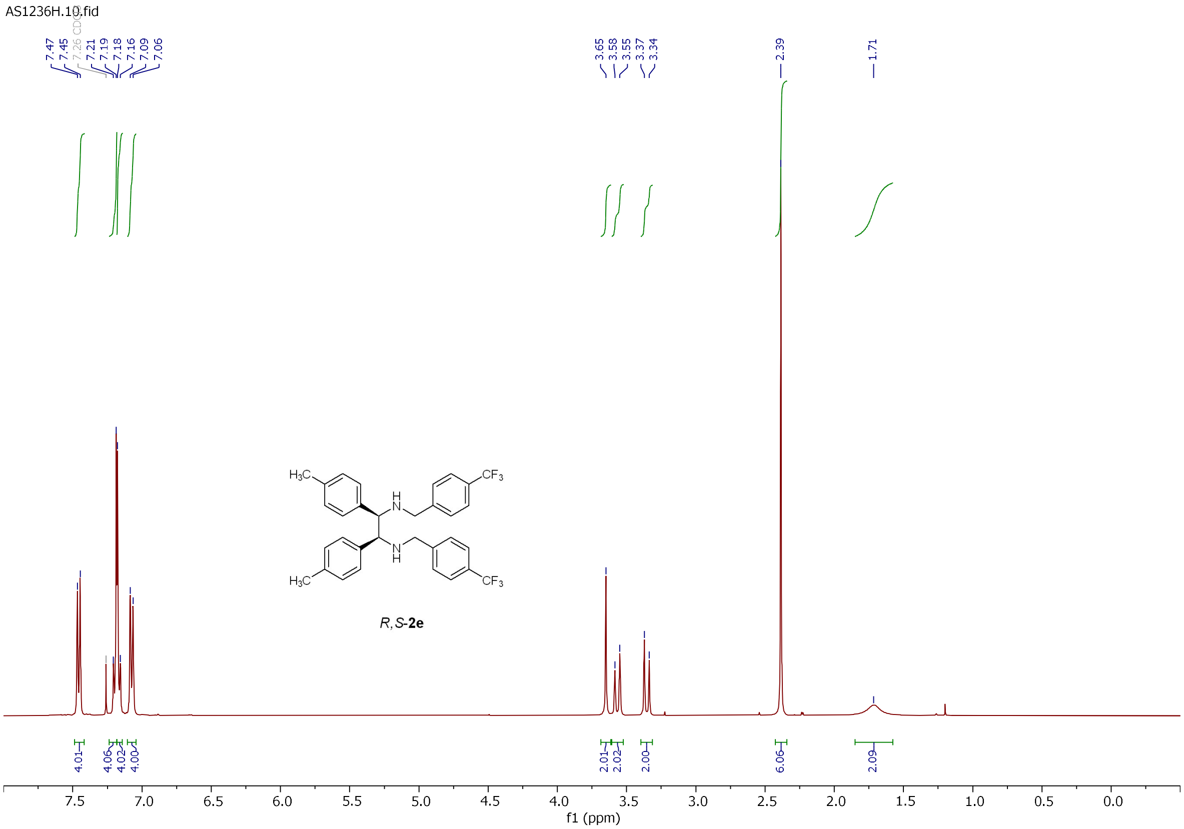

Supplementary Fig. 51. ***R,S*-2e ^{1}H NMR spectrum in $CDCl_3$.**

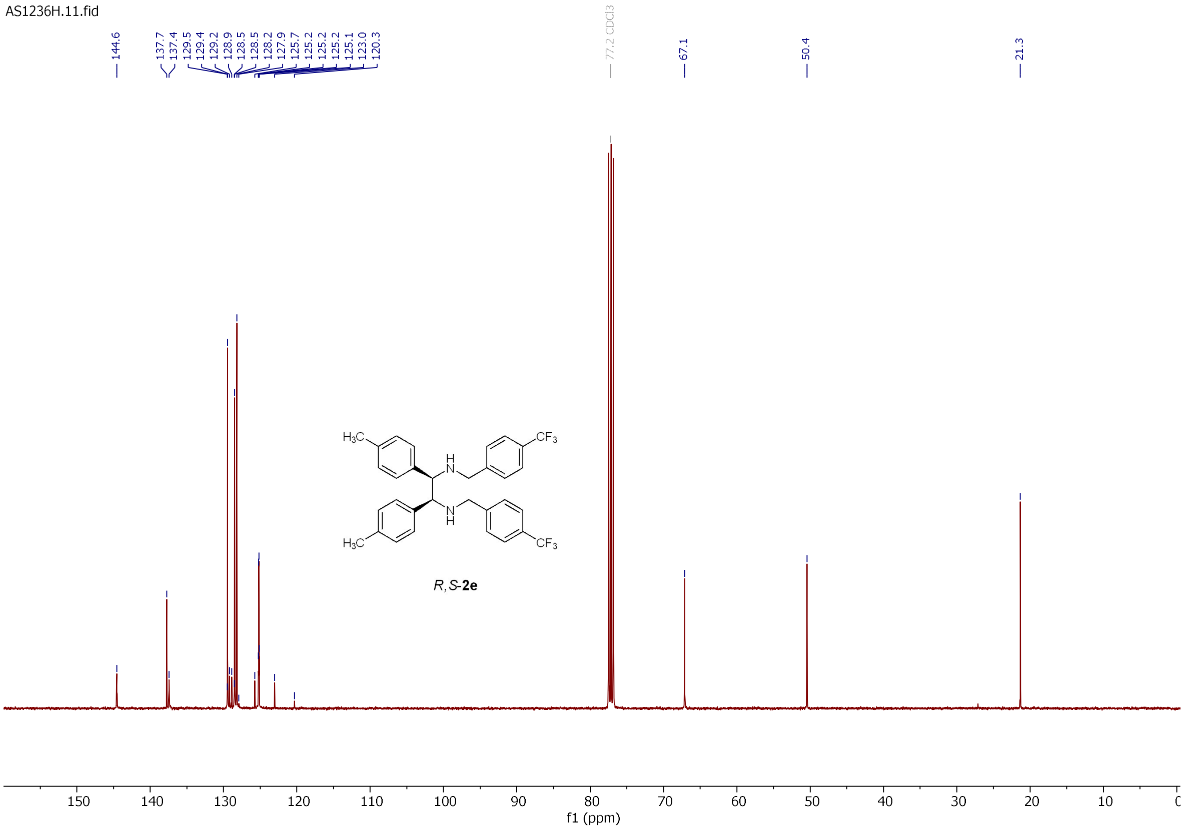

Supplementary Fig. 52. *R,S*-2e ^{13}C NMR spectrum in $CDCl_3$.

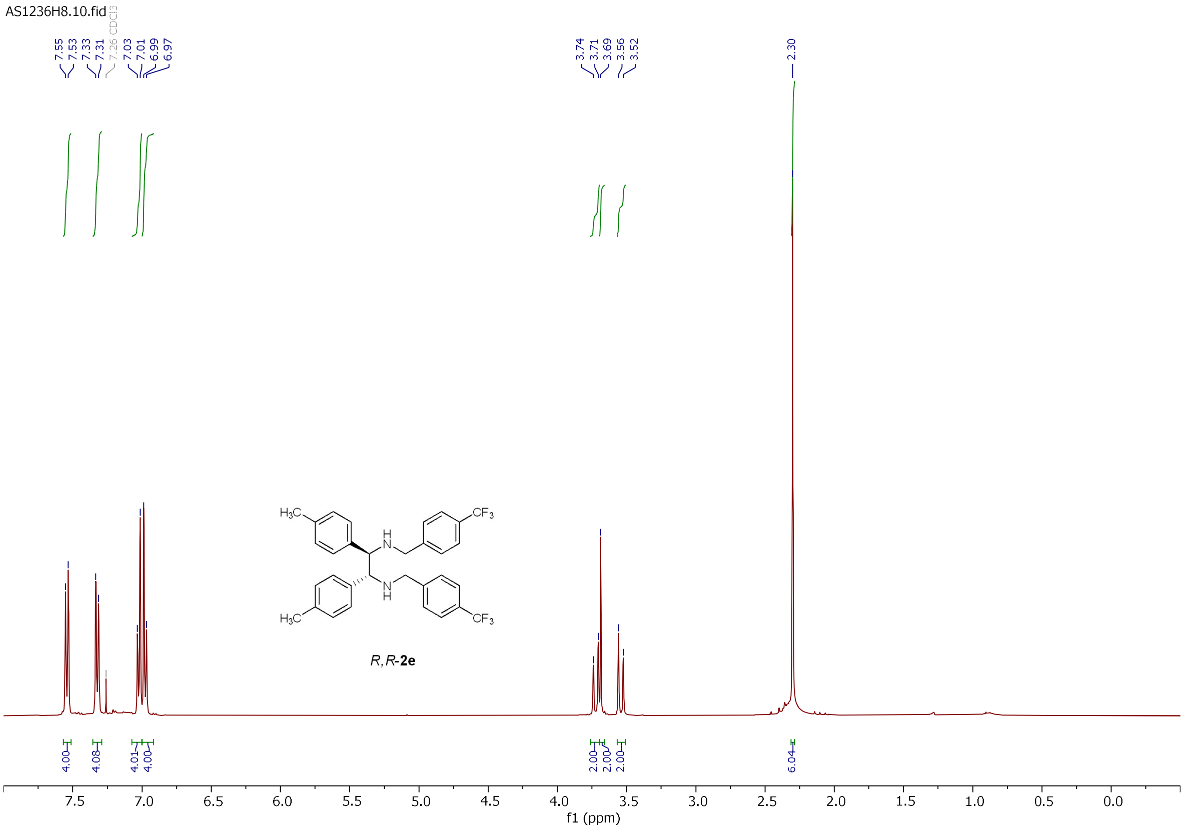

Supplementary Fig. 53. ***R*,*R*-2e ^{1}H NMR spectrum in $CDCl_3$.**

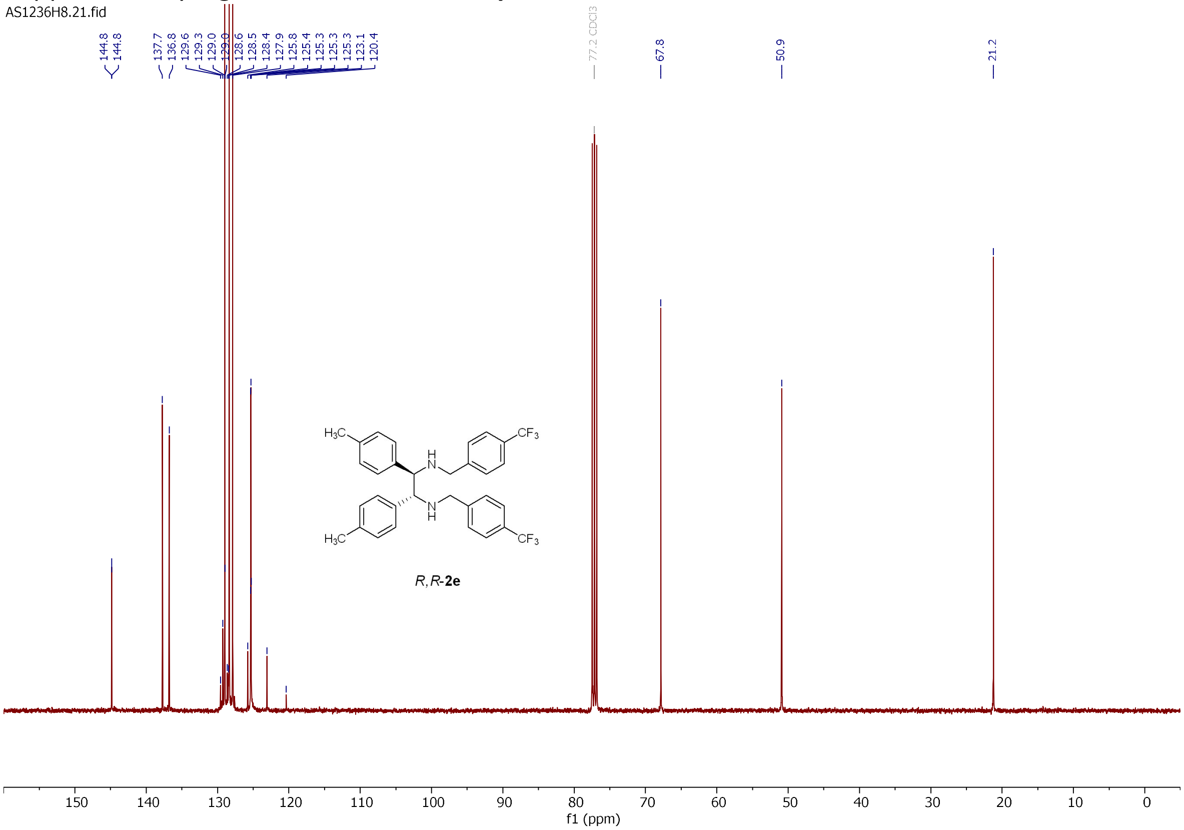

Supplementary Fig. 54. *R*,*R*-2e ^{1}H NMR spectrum in $CDCl_3$.

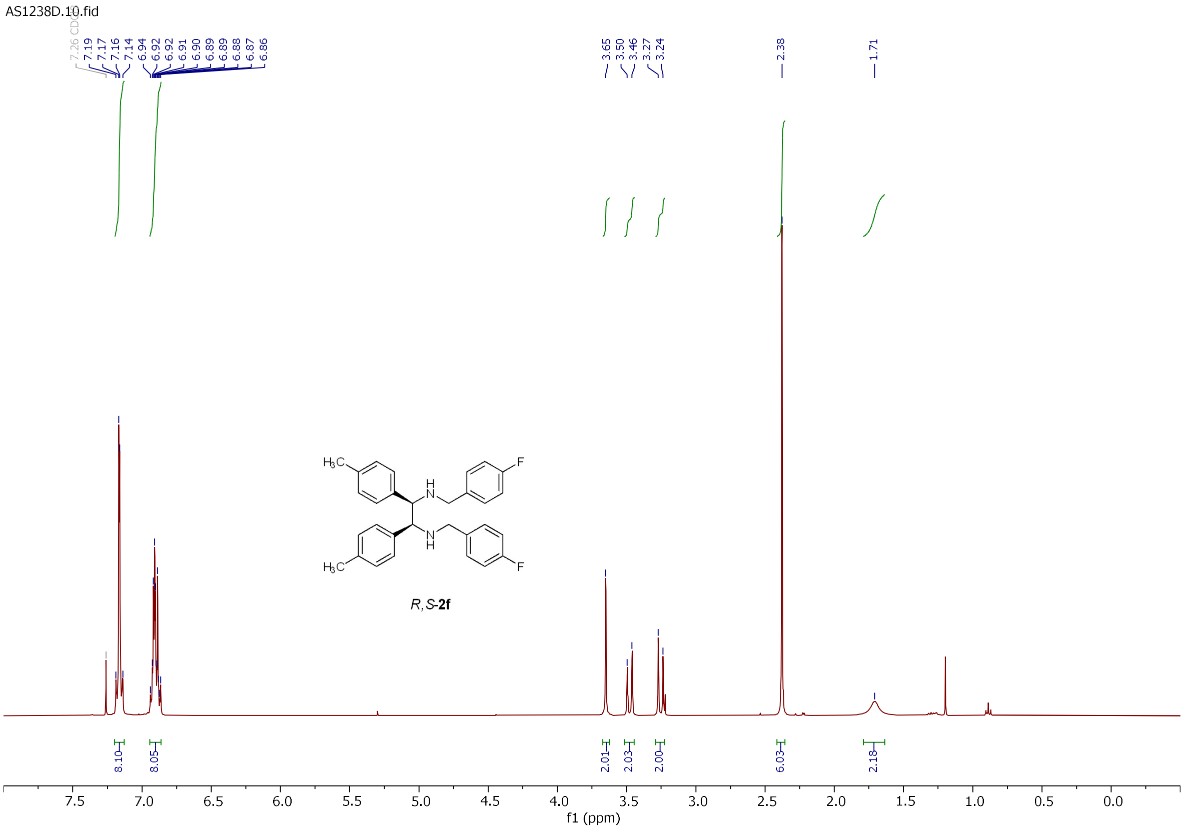

Supplementary Fig. 55. ***R,S*-2f ^{1}H NMR spectrum in $CDCl_3$.**

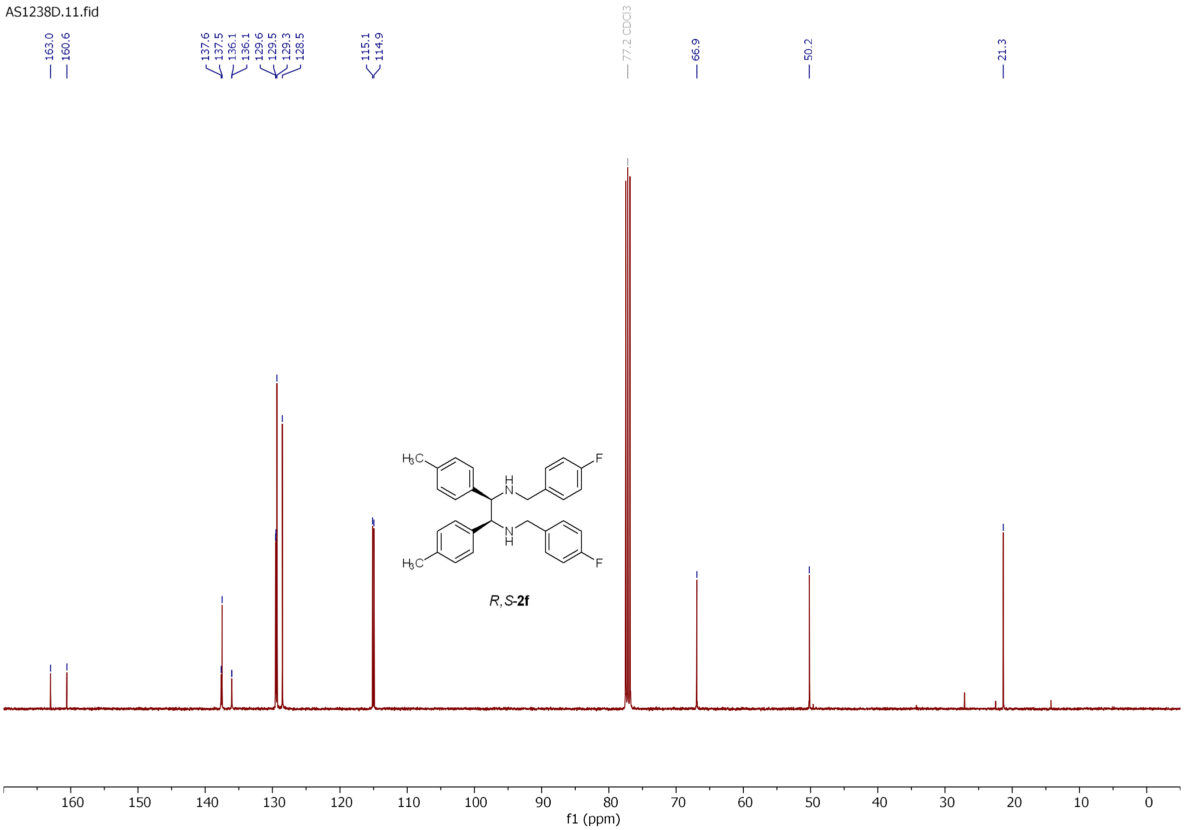

Supplementary Fig. 56. *R,S*-2f ^{13}C NMR spectrum in $CDCl_3$.

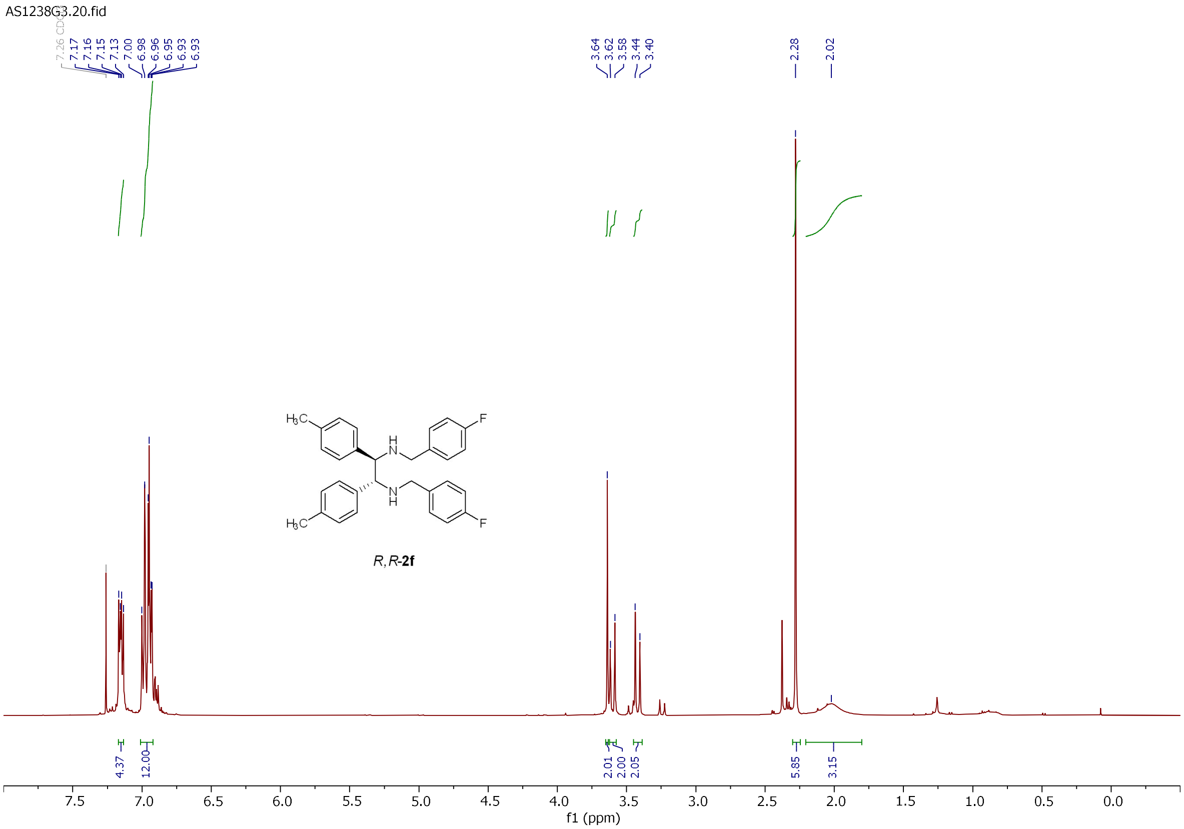

Supplementary Fig. 57. ***R,R*-2f ^{13}C NMR spectrum in $CDCl_3$.**

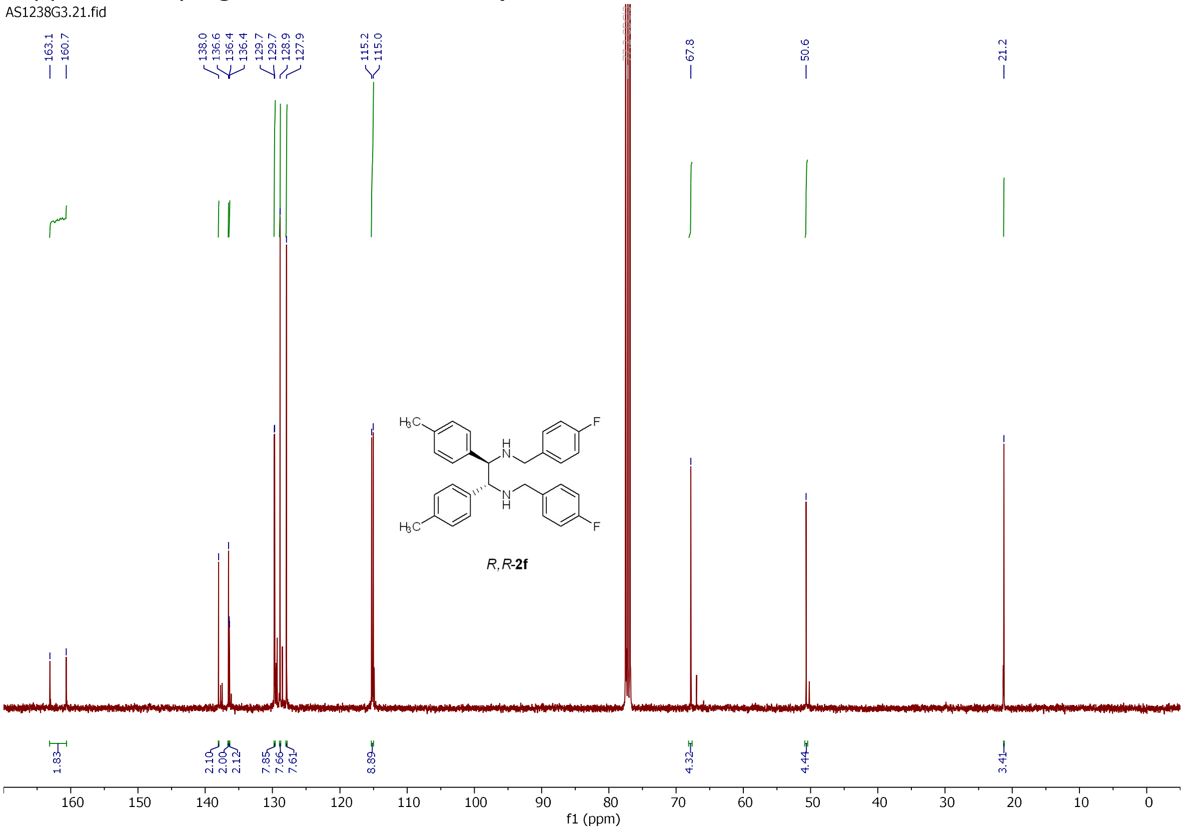

Supplementary Fig. 58. *R*,*R*-2f ^{13}C NMR spectrum in $CDCl_3$.

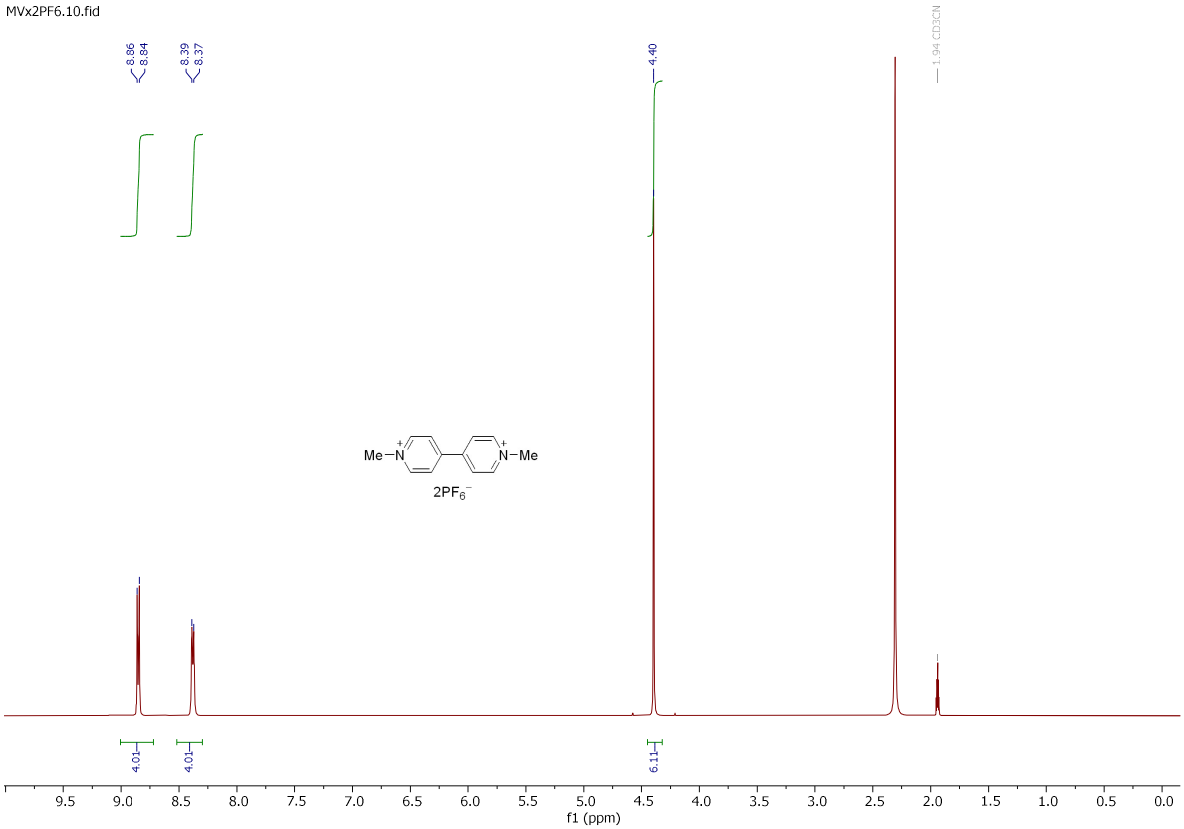

Supplementary Fig. 59. **MV^{2+} $2PF_6^-$ ^1H NMR spectrum in CD_3CN.**

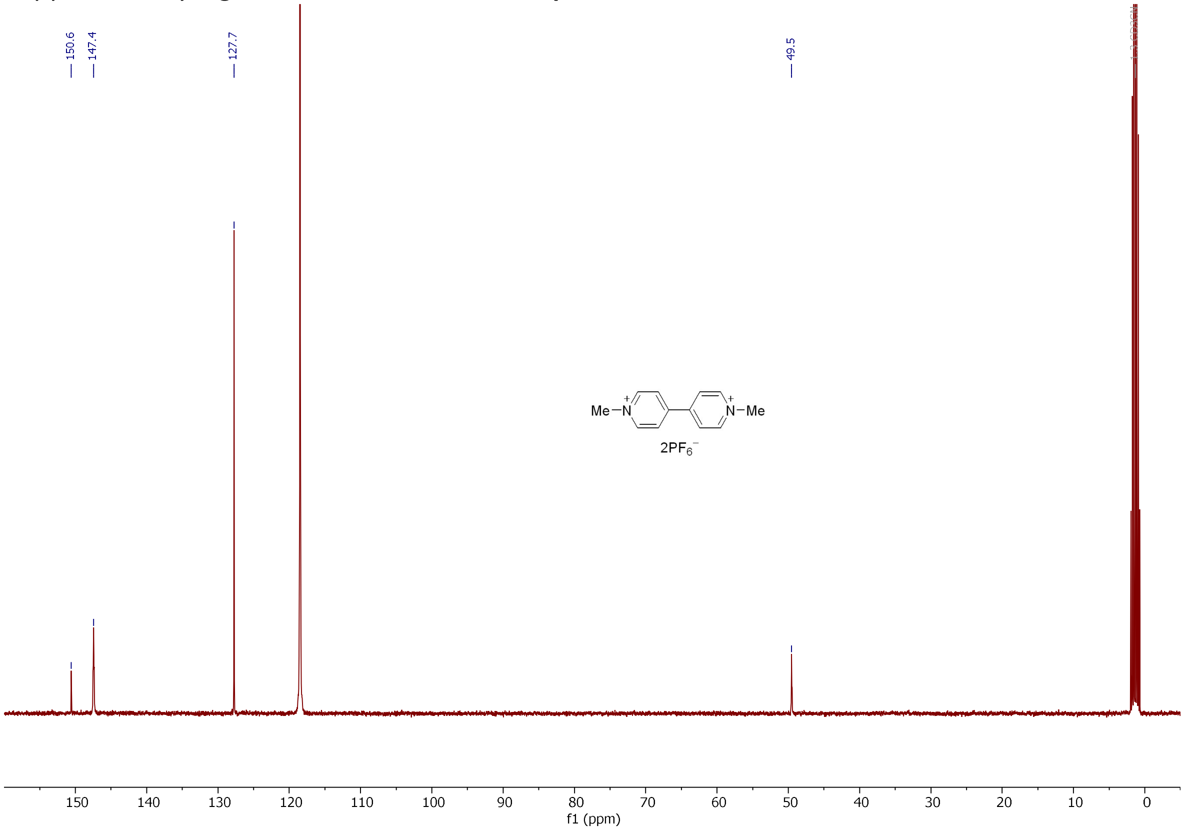

Supplementary Fig. 60. MV^{2+} $2PF_6^-$ ^{13}C NMR spectrum in CD_3CN.

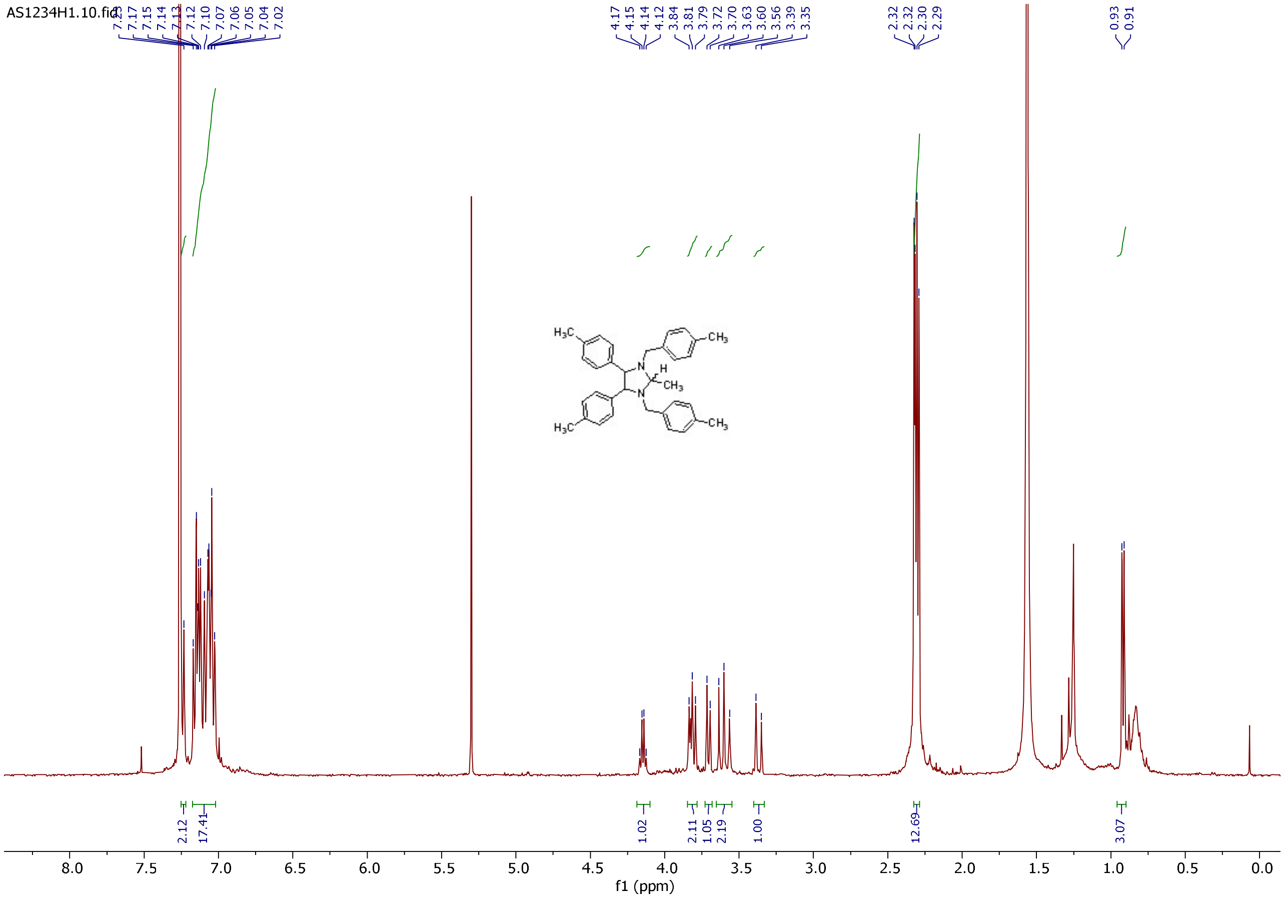

Supplementary Fig. 61. ^{1}**H NMR spectrum of imidazolidine 3.**

Supplementary References

1. Savateev A., Dontsova D., Kurpil B. & Antonietti M. Highly crystalline poly(heptazine imides) by mechanochemical synthesis for photooxidation of various organic substrates using an intriguing electron acceptor – Elemental sulfur. *J. Catal.* **350**, 203-211 (2017).

2. Chen Z.*, et al.* “The Easier the Better” Preparation of Efficient Photocatalysts—Metastable Poly(heptazine imide) Salts. *Adv. Mater.* **29**, 1700555 (2017).

3. Ghosh I., Khamrai J., Savateev A., Shlapakov N., Antonietti M. & König B. Organic semiconductor photocatalyst can bifunctionalize arenes and heteroarenes. *Science* **365**, 360-366 (2019).

4. Markushyna Y.*, et al.* Green radicals of potassium poly(heptazine imide) using light and benzylamine. *J. Mater. Chem. A* **7**, 24771-24775 (2019).

5. Watanabe T. & Honda K. Measurement of the extinction coefficient of the methyl viologen cation radical and the efficiency of its formation by semiconductor photocatalysis. *J. Phys. Chem.* **86**, 2617-2619 (1982).

6. Savateev A.*, et al.* Potassium Poly(Heptazine Imide): Transition Metal-Free Solid-State Triplet Sensitizer in Cascade Energy Transfer and [3+2]-cycloadditions. *Angew. Chem., Int. Ed.* **59**, 15061-15068 (2020).

7. Tsukinoki T., Nagashima S., Mitoma Y. & Tashiro M. Organic reaction in water. Part 4. New synthesis of vicinal diamines using zinc powder-promoted carbon–carbon bond formation. *Green Chem.* **2**, 117-119 (2000).

8. Savateev O., Tarakina N. V., Tyutyunnik A. P., Rivadeneira S. M., Heske J. & Kühne T. D. Assignment of the Crystal Structure to the Aza-Pinacol Coupling Product by X-ray Diffraction and Density Functional Theory Modeling. *ACS Omega*, (2022).

9. Lau V. W.-h.*, et al.* Dark Photocatalysis: Storage of Solar Energy in Carbon Nitride for Time-Delayed Hydrogen Generation. *Angew. Chem., Int. Ed.* **56**, 510-514 (2017).

10. Savateev A., Pronkin S., Willinger M. G., Antonietti M. & Dontsova D. Towards Organic Zeolites and Inclusion Catalysts: Heptazine Imide Salts Can Exchange Metal Cations in the Solid State. *Chem. - Asian J.* **12**, 1517-1522 (2017).

11. Kröger J.*, et al.* Conductivity Mechanism in Ionic 2D Carbon Nitrides: From Hydrated Ion Motion to Enhanced Photocatalysis. *Adv. Mater.* **34**, 2107061 (2022).

12. Savateev O. & Zou Y. Identification of the Structure of Triethanolamine Oxygenation Products in Carbon Nitride Photocatalysis. *ChemistryOpen* **11**, e202200095 (2022).

13. Ghosh T., Das A. & König B. Photocatalytic N-formylation of amines via a reductive quenching cycle in the presence of air. *Org. Biomol. Chem.* **15**, 2536-2540 (2017).

14. Wada Y., Kitamura T. & Yanagida S. CO2-fixation into organic carbonyl compounds in visible-light-induced photocatalysis of linear aromatic compounds. *Res. Chem. Intermed.* **26**, 153-159 (2000).

15. Cook S. K. & Horrocks B. R. Heterogeneous Electron-Transfer Rates for the Reduction of Viologen Derivatives at Platinum and Bismuth Electrodes in Acetonitrile. *ChemElectroChem* **4**, 320-331 (2017).

16. Hu J., Wang J., Nguyen T. H. & Zheng N. The chemistry of amine radical cations produced by visible light photoredox catalysis. *Beilstein J. Org. Chem.* **9**, 1977-2001 (2013).

17. Pavlishchuk V. V. & Addison A. W. Conversion constants for redox potentials measured versus different reference electrodes in acetonitrile solutions at 25°C. *Inorg. Chim. Acta* **298**, 97-102 (2000).

18. Lotsch B. V., *et al.* Unmasking Melon by a Complementary Approach Employing Electron Diffraction, Solid-State NMR Spectroscopy, and Theoretical Calculations—Structural Characterization of a Carbon Nitride Polymer. *Chem. - Eur. J.* **13**, 4969-4980 (2007).

19. Schlomberg H., *et al.* Structural Insights into Poly(Heptazine Imides): A Light-Storing Carbon Nitride Material for Dark Photocatalysis. *Chem. Mater.* **31**, 7478-7486 (2019).

20. James M. J., Schwarz J. L., Strieth-Kalthoff F., Wibbeling B. & Glorius F. Dearomative Cascade Photocatalysis: Divergent Synthesis through Catalyst Selective Energy Transfer. *J. Am. Chem. Soc.* **140**, 8624-8628 (2018).

21. Metternich J. B. & Gilmour R. One Photocatalyst, n Activation Modes Strategy for Cascade Catalysis: Emulating Coumarin Biosynthesis with (−)-Riboflavin. *J. Am. Chem. Soc.* **138**, 1040-1045 (2016).

22. Neveselý T., Daniliuc C. G. & Gilmour R. Sequential Energy Transfer Catalysis: A Cascade Synthesis of Angularly-Fused Dihydrocoumarins. *Org. Lett.* **21**, 9724-9728 (2019).

23. Ma J., *et al.* Facile access to fused 2D/3D rings via intermolecular cascade dearomative [2 + 2] cycloaddition/rearrangement reactions of quinolines with alkenes. *Nat. Catal.* **5**, 405-413 (2022).

24. Yan D.-M., Zhao Q.-Q., Rao L., Chen J.-R. & Xiao W.-J. Eosin Y as a Redox Catalyst and Photosensitizer for Sequential Benzylic C−H Amination and Oxidation. *Chem. - Eur. J.* **24**, 16895-16901 (2018).

25. Wang H., *et al.* Visible light-induced cyclization reactions for the synthesis of 1,2,4-triazolines and 1,2,4-triazoles. *Chem. Commun.* **53**, 9644-9647 (2017).
